# Autophagy Activators Normalize Aberrant Tau Proteostasis and Rescue Synapses in Human Familial Alzheimer's Disease iPSC‐Derived Cortical Organoids

**DOI:** 10.1002/advs.202514783

**Published:** 2026-01-27

**Authors:** Sergio R. Labra, Jadon Compher, Akhil Prabhavalkar, Mireya Almaraz, Claudia Cedeño Kwong, Christine Baal, Maria Talantova, Nima Dolatabadi, Julian Piña‐Sanz, Yubo Wang, Leonard Yoon, Swagata Ghatak, Zi Gao, Yuting Zhang, Dorit Trudler, Lynee Massey, Wei Lin, Anthony Balistreri, Michael Bula, Nicholas J. Schork, Tony S. Mondala, Steven R. Head, Jeffery W. Kelly, Stuart A. Lipton

**Affiliations:** ^1^ Neurodegeneration New Medicines Center The Scripps Research Institute La Jolla California USA; ^2^ Department of Molecular and Cellular Biology The Scripps Research Institute La Jolla California USA; ^3^ Department of Chemistry The Scripps Research Institute La Jolla California USA; ^4^ School of Biological Sciences National Institute of Science Education and Research Bhubaneswar Odisha India; ^5^ Translational Genomics Research Institute Phoenix Arizona USA; ^6^ Genomics Core The Scripps Research Institute La Jolla California USA

**Keywords:** Alzheimer's disease, autophagy, cortical organoids, pTau oligomers, presenilin 1

## Abstract

Alzheimer's disease (AD) is the leading cause of dementia worldwide. Nevertheless, its cellular and molecular mechanisms remain incompletely understood, partially due to inadequate disease models. To illuminate early changes in AD, we developed a cerebrocortical organoid (CO) model with improved methodology. Our COs produce excitatory and inhibitory neurons alongside glia, utilizing established isogenic wild‐type and diseased human induced pluripotent stem cells (hiPSCs) carrying heterozygous familial AD mutations in PSEN1^ΔE9^/WT, PSEN1^M146V^/WT, or APP^Swe^/WT. In addition to amyloid‐beta (Aβ) accumulation, the AD COs display time‐progressive loss of monomeric Tau, and accumulation of aggregated high‐molecular‐weight (HMW) phospho(p)‐Tau species (pT181 and pT217). They also exhibit neuronal hyperexcitability reminiscent of early electroencephalography (EEG) clinical findings and synapse loss in AD patient brains. Single‐cell RNA‐sequencing analyses of AD and WT control COs reveal significant divergent molecular abnormalities in excitatory vs. inhibitory neurons, with several pathways being upregulated in one while downregulated in the other, providing insight into AD phenotypes. Finally, we show that chronic dosing with autophagy activators, including a novel mTOR inhibitor‐independent drug candidate, prevents pathologic Aβ and HMW p‐Tau accumulation, normalizes hyperexcitability, and rescues synaptic loss in AD COs. Collectively, our results demonstrate this CO model as a useful platform for assessing early features of familial AD pathogenesis and for testing small‐molecule candidate therapeutics.

## Graphical abstract

A new cerebrocortical organoid model using isogenic hiPSCs with familial Alzheimer's mutations recapitulates key AD features, including amyloid‐beta and phospho‐Tau aggregation, neuronal hyperexcitability, and synapse loss. Single‐cell RNA‐seq reveals aberrant pathways in excitatory and inhibitory neurons. Known and novel autophagy activators prevent pathological and synaptic deficits, supporting the model's utility for AD research and drug screening in the setting of early disease.

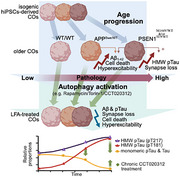

## 1 Introduction

Alzheimer's disease (AD) is a neurodegenerative disorder, currently affecting over 47 million people worldwide, characterized by synapse and neuronal loss leading to progressively declining cognitive function [1, 2]. The consensus view of AD pathology is that Aβ aggregation, triggering hyperphosphorylated Tau aggregation, leads to neuronal dysfunction and ultimately brain cell death [3]. These aggregates compromise membrane integrity and organelle function, while also altering transcriptional programs and engaging signaling pathways, leading to multiple mechanisms of pathobiology, including neuroinflammation [2, 4]. Most AD therapies targeting amyloid beta (Aβ) — including β‐secretase or γ‐secretase inhibitors and aggregation blockers — have failed in clinical trials due to limited efficacy or adverse effects. Recent exceptions are the FDA‐approved anti‐Aβ antibodies lecanemab and donanemab, which modestly slow progression of AD‐associated mild cognitive impairment by 25%–30%, in part via microglia‐mediated lysosomal clearance of Aβ aggregates [5]. In contrast, to date, no clinically approved therapies effectively halt Tau pathology. Although early‐stage active immunotherapy against pathological Tau protein shows limited promise in Aβ/Tau‐positive patients, most have failed [6, 7]. Collectively, these clinical findings underscore Aβ aggregation as a driver of AD progression, with Tau pathology and neuroinflammation as additional contributing factors.

The advent of human induced pluripotent stem cells (hiPSCs) derived from AD patients harboring mutations in amyloid precursor protein (APP) or presenilin 1 (PSEN1; one of four proteins in the γ‐secretase complex) and the generation of isogenic, gene‐corrected controls afforded us the opportunity to capture the earliest changes associated with AD longitudinally in specific differentiated tissues of interest. While traditional two‐dimensional (2D) cultures have numerous practical advantages, they fall short in encompassing the complexity of neural tissue [8, 9]. Advances in 3D culture systems now enable the creation of self‐organizing cerebrocortical organoids (COs). These COs recapitulate essential aspects of human brain development, including the development of multiple cellular identities like astrocytes and diverse neuronal classes, though notably lacking microglial cells [10, 11, 12, 13]. Previous studies using hiPSC‐derived neuronal and astrocytic 3D co‐cultures from AD patients have shown the formation of Aβ aggregates and Tau neurofibrillary tangles (NFTs)—hallmark pathologies not easily observed in most animal or 2D culture models [14, 15, 16, 17, 18]. These pathological molecular phenotypes are attenuated or reversed by β‐secretase and γ‐secretase inhibitor treatment, validating animal model findings and demonstrating the drug‐testing potential of 3D culture models [18, 19, 20]. We reasoned that a CO‐based AD model would allow for a more pathophysiologically faithful cellular function and response system characterization than 2D culture models, due to its intricate and diverse cellular organization, albeit for early events in disease pathogenesis. This strategy has been previously proposed by others, especially with regard to the potential utility for testing novel therapeutic candidates [14, 15, 16, 21, 22, 23, 24, 25, 26]. Nonetheless, concerns still exist regarding the use of COs as models of intrinsically advanced age‐related diseases like AD [27, 28, 29, 30, 31, 32, 33]. It is currently unclear how these relatively young cellular models recapitulate phenotypes like NFTs that would normally take decades to develop in the human brain. Thus, we set out to develop and robustly validate an improved CO‐based model to more faithfully represent the complex pathophysiological changes and cell‐cell interactions in the early stages of familial AD (fAD). This model would facilitate the study of both progressive responses to accumulating pathologic stress by different cell types and the potential causes behind this expedited pathology.

Previous studies have shown that hiPSC‐derived neurons harboring the APP mutation recapitulate several key aspects of AD pathology, including elevated Aβ_1‐42_ or Aβ_1‐42_/Aβ_1‐40_ ratio, increased Tau phosphorylation, and neuronal stress [34, 35]. However, these studies were limited by the use of 2D monocultures that lacked the complexity and cellular diversity of organoid cultures. Meanwhile, most studies on these PSEN1 mutations have been limited to clinical genetic or postmortem correlations with limited mechanistic insight [36, 37, 38, 39, 40, 41], while the few in vitro studies performed to date have been limited in scope [19, 42, 43, 44, 45].

Additionally, several lines of evidence suggest that the autophagy‐lysosomal pathway is impaired in AD, contributing to the accumulation of toxic protein aggregates and neurodegeneration, reflecting aberrant proteostasis [46]. Postmortem brain tissues from AD patients exhibit an accumulation of autophagosomes and lysosomes, indicating a defect in autophagosome‐lysosome fusion and lysosomal degradation [47]. Moreover, familial AD‐linked mutations in PSEN1 and amyloid APP have been shown to disrupt lysosomal acidification and proteolysis, leading to autophagic dysfunction. These findings have led to the hypothesis that enhancing autophagy and lysosomal function could be a promising therapeutic strategy for AD [48, 49]. Indeed, various autophagy activators, such as rapamycin, trehalose, and small molecule enhancers of rapamycin (SMERs), have been reported to reduce Aβ and Tau pathology to some degree in immortalized cell lines, as well as to alleviate cognitive deficits and slow disease progression in transgenic AD mouse models [50, 51, 52]. However, the optimal dosing regimen and long‐term efficacy of these compounds remain to be established. Here, we make initial strides to establish a CO dosing strategy employing mTOR inhibitor‐independent autophagy activators. This is particularly important as the US Food and Drug Administration (FDA) has recently signaled that it may accept such data in lieu of and in preference to animal data for future drug approvals [53, 54].

In this study, we employed previously established gene‐edited hiPSC lines carrying heterozygous fAD mutations in the genes encoding presenilin 1 (PSEN1) or amyloid precursor protein (APP) and compared results to their isogenic, wild‐type (WT)‐gene controls [35, 55]. Specifically, we used a PSEN1^M146V^/WT missense mutation (M146V/WT), which enhances Aβ_1–42_ peptide (Aβ_1‐42_) production, leading to an increased Aβ_1‐42_/Aβ_1‐40_ ratio, a hallmark associated with an aggressive early‐onset AD phenotype. Additionally, the APP^Swe^/WT double mutation (K670N/M671L) residing directly upstream of the β‐secretase cleavage site, makes APP a more efficient substrate, leading to increased total Aβ production. Together, these mutants comprise our isogenic set 1 [56, 57, 58]. To contrast with a different genetic background [59], we also utilized a line with the PSEN1^ΔE9^/WT mutation (ΔE9/WT), an in‐frame deletion of exon 9 (residues 291–319) that leads to impaired endoproteolytic processing of PSEN1, which is also known to result in an increased Aβ_1‐42_/Aβ_1‐40_ ratio [36, 60]. This mutation was introduced into a separate, well‐established hiPSC line (with ΔE9/WT and WT/WT (2) cell lines comprising isogenic set 2) [42, 61].

Herein, we report the development of a thoroughly characterized human AD CO model based on hereditary AD mutants and isogenic controls. We describe a protocol that reproducibly generates COs and show that the COs exhibit the hallmark biochemical, transcriptomic, and functional abnormalities of AD. Furthermore, we demonstrate their use to interrogate, in real time, the effects of using autophagic induction as a therapeutic strategy.

## 2 Results

### 2.1 Development of a Consistent and Robust AD CO Model

**FIGURE 1:**
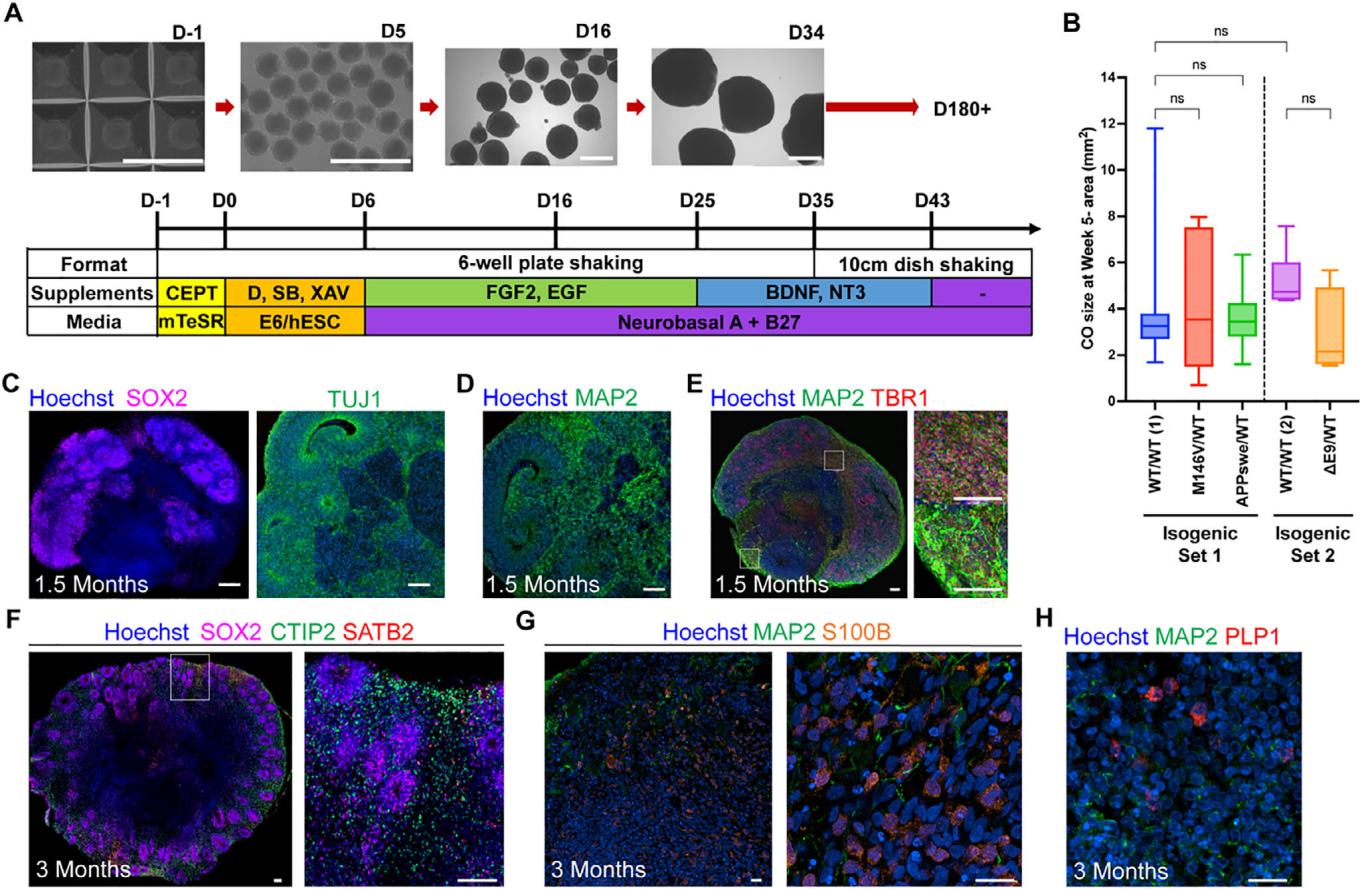
Generation of isogenic WT and AD COs. (A) Schematic describing the differentiation of cerebrocortical organoids (COs) with representative brightfield images. Scale bar, 1 mm. (B) Quantification of the CO maximal cross‐sectional area distribution across genotypes at approximately week 5 in culture, *n* = 13–26 COs per genotype for isogenic Set 1, *n* = 6–9 COs per genotype; each from 4 independent CO induction batch experiments. Data are mean ± SD. Analysis by ANOVA with Dunnett's post‐hoc test. (C) Representative immunofluorescence (IF) stains of neuroepithelial (SOX2^+^) and neuronal progenitor (TUJ1^+^) cells at ∼7‐week timepoint COs. Scale bar, 100 µm. (D) Representative IF stains of mature postmitotic neurons (MAP2^+^) in COs at ∼7‐week timepoint. Scale bar, 100 µm. (E) Representative IF stains for mature cortical layer neurons (TBR1^+^) cells in a 7‐week timepoint CO with two highlighted insets to the right. Scale bar, 100 µm. (F) Representative IF stains for deep layer V (CTIP2^+^) and upper layer II‐III (SATB2^+^) cells in a 3‐month timepoint CO with inset to the right. Scale bars, 100 µm. (G) Representative IF stains of astrocytes (S100β^+^) 3‐month timepoint COs. Scale bars, 25 µm. (H) Representative IF stains of oligodendrocytes (PLP1^+^) 3‐month timepoint COs. Scale bar, 25 µm.

To consistently produce comparable COs across all our cell line genotypes, we started with the elegant method published by Paşca and colleagues, which we designate Protocol 1 [62]. We optimized Protocol 1 to minimize CO fusion, prevent CO attachment to the surface, reduce self‐disassembly, and improve viability in our COs, generating protocol 2 (Figure 1A and Figure S1A). Maintaining the COs under constant mixing in ULA 6‐well plates by employing an in‐incubator rocker and/or tilted orbital shaker (15° incline, 65 rpm) significantly improved CO viability and morphology (Figure S1B). Staining live COs with propidium iodide at around the 4‐month timepoint showed that necrosis was also reduced, suggesting improved nutrient absorption within the COs (S1C). Next, we studied the morphology and size distribution of COs produced by modifying the induction medium. We compared Protocol 1's Gibco's Essential 6 medium (E6) vs. different concentrations of hESC medium, given the latter's use in other CO induction methods [12, 63]. We found that a mixture of 50% E6 and 50% hESC medium (E6/hESC) yielded larger COs with more pronounced neuroectoderm morphology than those grown in E6 alone (Figure S1D–F), designated herein as Protocol 2. Importantly, Protocol 2 with the E6/hESC medium produced COs without significant size differences across all the different genotypes by the 5‐week time point in culture (Figure 1B and Figure S1G). Despite the COs consistently growing larger in size, they showed dramatically lower levels of necrosis compared to those grown in parallel following protocol 1 (Figure S1C).

To scrutinize the differentiation quality of the COs, we performed immunohistochemical labeling of fixed, sliced CO samples. At day (D)50, SOX2 and TUJ1 staining demonstrated the presence of neural progenitors most densely accumulated around ventricular‐like rosette formations (Figure 1C). Meanwhile, MAP2^+^ mature neurons populated the large majority of the COs outside the margin of these rosettes (Figure 1D); NeuN was also used as a mature neuron marker (Figure S1H). Staining for FOXG1, a critical forebrain transcription factor key in cerebrocortical development, showed the organoids to be maturing toward forebrain identity (Figure S1I). Furthermore, TBR1 staining revealed a large number of MAP2^+^/TBR1^+^ cells, indicating the presence of deep cortical layer neurons in the developing CO (Figure 1E). Staining neurons in various cerebrocortical layers using layer‐specific markers, including SATB2 (upper cortical layers 2–4) and CTIP2 (deep cortical layers 5–6), showed non‐overlapping positive signals spatially distributed across the COs around the SOX^+^ rosettes (Figure 1F). Together, these findings strongly suggest that the cells within the developing COs emulate ventricular zone migration, and that the various neuronal layer subtypes are properly differentiating without acquiring overlapping subtype identities. Previously, this has been highlighted as a concern in other CO differentiation protocols [27].

Staining for astrocytes via S100B (Figure 1G) demonstrated that populations of astrocytes are already abundant in the COs at the 3‐month timepoint. Notably, we also found the presence of PLP1+ cells, putatively oligodendrocytes, (Figure 1H) by the 3‐month timepoint. The presence of these cells is particularly striking given that previous CO‐based studies have struggled to generate any or enough of these critical glial cells [64].

**FIGURE 2:**
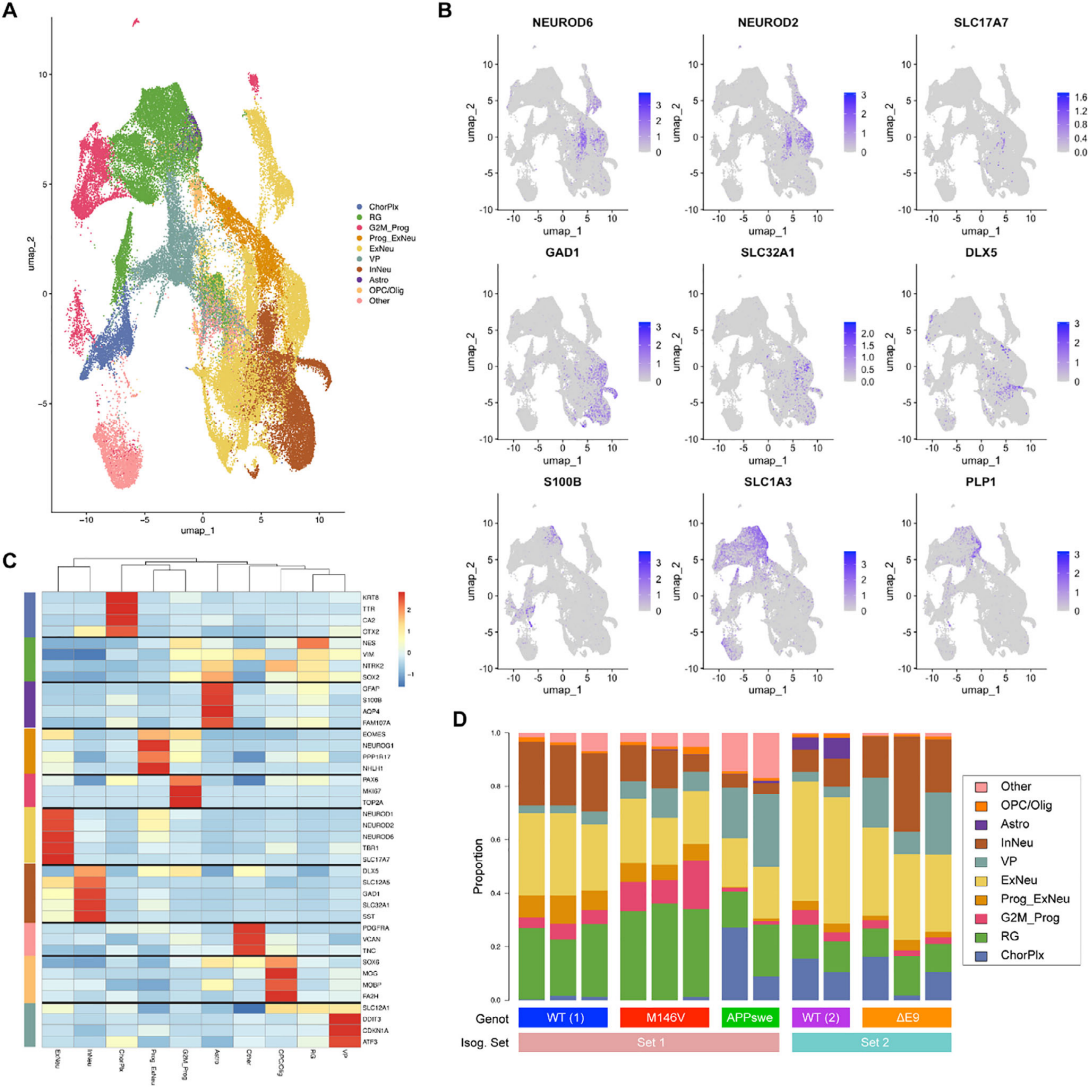
COs recapitulate key cellular diversity. (A) Annotated UMAP of CO's scRNA‐seq at 2‐month and 3‐month timepoints, identifying major cell classes including excitatory neurons (ExNeu) and their progenitors (Prog_ExNeu), inhibitory neurons (InNeu), ventral progenitors (VP), choroid plexus (ChrPlx), radial glia (RG), astrocytes (Astro), Oligodendrocyte progenitor cells and oligodendrocytes (OPC/Olig), and dividing (in G2/M phase) progenitors (G2M_Prog). *n* = 27 COs in total from five independent experiments. (B) Feature plots demonstrating the specific segregation of cell‐types by hallmark gene expression of key cell type markers. Highlighted here are Excitatory neurons (NEUROD6 and SLC17A6, aka VGLUT2); Inhibitory neurons (GAD1 and SLC32A1); Astroglia (S100B), and Oligodendrocytes (PLP1). (C) Heatmap ordered by similarity showing the differential expression of hallmark marker genes in all identified cell subtypes. See also Figure S2F. (D) Bar plot showing the relative composition or abundance of cell types in the COs at 3‐month timepoint across genotypes (Genot) and isogenic sets (Isog. Set).

To further characterize the cellular diversity and reproducibility of our COs, we performed single‐cell RNA sequencing (scRNA‐seq) at two timepoints, after ∼7–8 weeks and 3 months in culture. We multiplexed isogenic WT and fAD COs differentiated in parallel using the 10x Genomics platform (Illumina) at ∼40 K reads per sample across multiple independent experiments. Each sequencing dataset underwent separate, stringent quality control before integrating and reprocessing for annotation and validation (Figure S2A; Experimental Section). The final dataset consisted of 70,854 cells. Overall, there was significant overlap between the two timepoints (Figure S2B), and each AD and WT genotype contributed to all unsupervised clusters (Figure S2C,D), indicating the clustering was not significantly driven by genotype‐specific differences. We identified ten major cell‐type groups (Figure 2A). The localized expression of key hallmark markers showed the specificity of each validated cell‐type (Figure 2B and Figure S2E) [65, 66, 67, 68, 69].

As examples, expression of NEUROD6, NEUROD2, and SLC17A7 reflected excitatory neurons; GAD1, SLC32A1, and DLX5, inhibitory interneurons; SLC1A3, glial cells; S100B, astrocytes; and PLP1, oligodendrocytes, specifically. Cell‐type similarity analysis agreed with the expression and relatively low overlap in key hallmark genes across the different cell types, suggesting proper differentiation and maturation (Figure 2C and Figure S2F). Of particular interest, the presence of oligodendrocytes was also validated by their unique expression of additional markers like MOG, MOBP, and FA2H. We also note that across all the samples, the COs are most similar to COs sharing their own genotype with minimal variation between them, showcasing consistency (Figure 2D and Figure S2G). These results suggest our COs reproducibly developed mature cortex‐relevant cell types, including excitatory and inhibitory neurons and their progenitors, astroglia, oligodendrocyte progenitor cells (OPCs), and oligodendrocytes.

### 2.2 AD Mutation Drives Stress‐ and Senescence‐related Cell Phenotypes in CO and Abnormal Neuronal Development

We note that the VM‐, SOX2‐, and SLC12A1‐positive CO ventral progenitors (VP) found through scRNA‐seq were highly enriched in markers of cellular stress, including DDIT3, CDKN1A (p21), and ATF3 (Figure 2C) [70, 71, 72, 73, 74]. These “stressed” progenitors were significantly more abundant in the PSEN1^M146V^/WT, APP^Swe^/WT, and PSEN1^ΔE9^/WT COs compared to their isogenic WT/WT controls (Figure S2H). Other differences included a significantly reduced proportion of interneurons (InNeu) in the PSEN1^M146V^/WT and APP^Swe^/WT COs and reduced excitatory neurons (ExNeu) in all AD mutant COs compared to their isogenic WT/WT COs (Figure S2H).

We confirmed these scRNA‐seq results through immunolabeling for vesicular glutamate transporter 1 (VGLUT1) and γ‐aminobutyric acid (GABA) on fixed slices from 6‐week and 3‐month‐old COs to estimate the populations of excitatory and inhibitory neurons, respectively (Figure S3A,B). Their relative abundance generally agreed with the scRNA‐seq results; namely, we confirmed an age‐progressing relative reduction of ExNeu in the APP^Swe/^WT and PSEN1^ΔE9^/WT COs (Figure S3C), and a decrease in InNeu in the PSEN1^M146V^/WT and APP^Swe/^WT COs vs. WT (Figure S3D). The relative abundance of these different neuronal cells is critical, given the importance of excitatory‐to‐inhibitory imbalance in AD, a pathological feature we reported previously in a related CO model [44, 75, 76].

Next, we performed pseudotime analysis on the whole dataset using the Monocle3 toolkit [77]. It re‐clustered the cells into several macro‐clusters, where the major cluster with the highest pseudotime values was composed of ramifying sub‐clusters of ExNeu or InNeu populations (Figure S4A,B). Notably, multiple branched neuronal populations stemmed from mostly single AD mutant genotypes. In particular, APP^Swe/^WT was highly enriched in specific ExNeu branches while the PSEN1^ΔE9^/WT was highly enriched at the end of both the ExNeu and InNeu trajectories, with the PSEN1^M146V^/WT mutant just behind it (Figure S4C). This trajectory analysis suggested the AD COs’ neurons were reaching additional neuronal states that their isogenic WT organoids were not, and these states were specific to the type of mutation involved (PSEN1 or APP). Furthermore, when looking at the pseudo‐time expression of hallmark ExNeu and InNeu markers, we find the expected progressive increase and slight decline in the immature ExNeu marker NEUROD2, as it gives way to maturing ExNeu, as marked by SLC17A7 (Figure S4D, ***left***). However, looking at InNeu progression, we noticed a sharp decline in both immature (DLX2) and mature (GAD2) InNeu marker expression (Figure S4E, ***left***). Repeating the pseudotime analysis without the AD mutants (WT/WT genotypes only) revealed not only a very clear trajectory from radial glia progenitors diverging into glial and neuronal cell types (Figure S4F–H), but the WT pseudotime neuron marker expression lacked the sharp decline in DLX2 and GAD2 expression (Figure S4E, ***right***). There was no notable difference between the two ExNeu marker expression pseudotime analyses (Figure S4D, ***right***). This demonstrates the decrease in InNeu marker expression is driven by the AD mutation. Together, this suggests that AD mutants may be developing into abnormal or vulnerable neurons as they age—which has been recently suggested for the APP^Swe^ mutation [78]—and in particular, they are affecting inhibitory neurons.

Next, we performed differential gene ontology (GO) enrichment analyses of the excitatory and inhibitory neurons in each of the AD mutant COs, compared to their isogenic WT CO controls (Figure S5A–F). Interestingly, we found both PSEN1 mutations leading to deficits in numerous synapse‐related pathways in excitatory neurons (Figure S5A,B), while synapse‐related terms were upregulated in the inhibitory neurons (Figure S5D,E). Similarly, the excitatory neurons of the PSEN1^M146V^/WT and PSEN1^ΔE9^/WT had significantly upregulated genes related to cytoplasmic RNA translation and ribosome biogenesis; and axon guidance and development, respectively (Figure S5A,B). The same pathways were depressed in both, PSEN1^ΔE9^/WT and APP^Swe^/WT inhibitory neuron mutants (Figure S5E,F). Finally, the APP^Swe^/WT excitatory neurons manifest different upregulated pathways; In particular, protein folding‐related stress responses were upregulated in excitatory neurons, while RNA processing was upregulated in inhibitory neurons (Figure S5C,F). These findings suggest these AD mutations affect starkly differently excitatory neurons from inhibitory neurons, which may in part explain the InNeu vulnerability and the underlying development of excitatory/inhibitory (E/I) imbalance in AD.

To further assess the similarity and relevance of the CO neurons to in vivo conditions, we compared their transcriptomic signatures to that of recently‐published vulnerable excitatory neurons identified by Gazestani et al. in early and/or pre‐clinical AD stages [79]. Principal component analysis (PCA) showed us the relative similarity of the CO ExNeu population to Gazestani et al.’s pre‐AD patient excitatory neurons and revealed the AD CO genotypes manifest increased similarity to the patient neurons compared to their isogenic WT controls (Figure S5G). Additionally, correlation analysis identified several vulnerable pre‐AD excitatory neuron subtypes to be highly similar to the CO ExNeu population (Figure S5H). Particularly interesting, early AD‐correlated neuronal subtypes based on gene expression, such as THEMIS_RXFP2, THEMIS_BCL11B, LINC00507_COL5A2, and LINC00507_ACVR1C, correlate much more strongly with the AD CO ExNeu than the WT ExNeu cell types. These data suggest our AD CO model may reproduce the transcriptomic signatures of pre‐clinical disease‐associated neurons.

Interestingly, the AD mutant COs were also found to generally have greater expression of senescence markers, including p21 (CDKN1A), p16 (CDKN2A), and the senescence‐associated secretory phenotype (SASP) [73, 74, 80]; this overexpression was weakly higher for the PSEN1^ΔE9^/WT, greater for the PSEN1^M146V^/WT, and strikingly increased in the APP^Swe^/WT mutant compared to their respective isogenic WT controls (Figure S5I). Additionally, comparing across genotypes, we found that markers of ferroptosis [81, 82] are notably elevated in the PSEN1^M146V^/WT (but not PSEN1^ΔE9^/WT) and APP^Swe^/WT COs vs. WT/WT (Figure S5J).

### 2.3 AD COs Recapitulate Amyloid‐β and Phospho‐Tau Signatures of Human AD Brain

**FIGURE 3:**
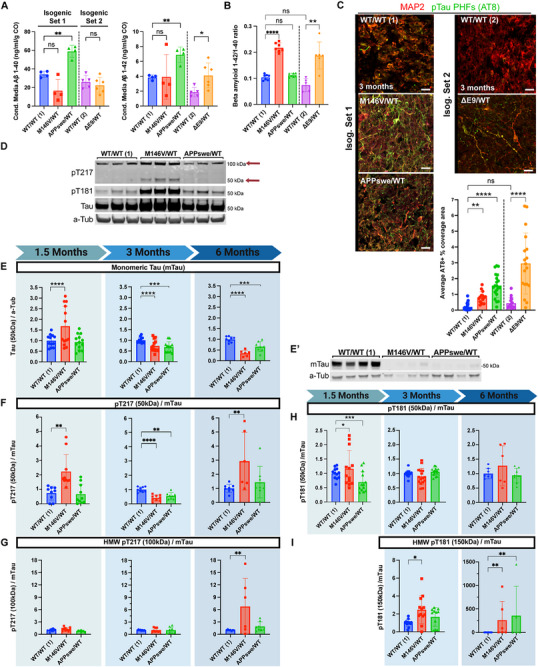
AD COs recapitulate amyloid and pTau pathologic signatures. (A) Aβ_1‐40_ and Aβ_1‐42_ concentrations in conditioned media from 5–6 months‐of‐age COs. *n* = 4–5 independent CO replicates per genotype. (B) Ratio of Aβ_1‐42_ to Aβ_1‐40_ from panel A. (C) Immunostaining of 3 months‐of‐age COs for Paired Helical Filament (PHF)‐Tau (AT8, green) and MAP2 (mature neurons, red). Scale bar, 20 µm; quantification below images. Isog. Set 1: *n* = 10–18 COs per genotype from three independent experiments; Isog. Set 2: *n* = 10–11 COs per genotype from two independent experiments. (D) Representative western blots (WB) of CO lysates at 1.5‐month timepoint for phosphorylated Tau pT217, pT181 at its MS‐validated monomeric molecular weight, pan‐Tau at monomeric molecular weight (mTau hereafter), and ɑ‐Tubulin (a‐Tub). (E) WB quantification of monomeric Tau (mTau) in CO lysates at the 1.5‐ (*left*), 3‐ (*center*), and 6‐month (*right*) timepoints, as normalized to ɑ‐Tubulin (a‐Tub). *n* = 8–14 COs per genotype from three independent experiments. (E’) Representative WB of mTau and a‐Tub in late‐stage (4.5‐month timepoint) CO lysates. (F‐G) WB quantification of phospho‐Tau pT217 at its monomeric (∼50 kDa)(F), and strongest high‐molecular‐weight (HMW)(∼100 kDa)(G) bands in CO lysates at the 1.5, 3, and 6‐month timepoints, as normalized to mTau. *n* = 5–12 COs per genotype from 3 independent experiments per timepoint. (H‐I) WB quantification of the pTau T181 bands at monomeric (∼50 kDa) (K) and strongest HMW (∼150 kDa) (L) bands at 1.5, 3, and 6‐month timepoints normalized to mTau. Data are mean ± SD. Analyses by ANOVA with Dunnett's post‐hoc test.

To characterize the extent to which the AD CO model recapitulates hallmark AD pathology, we measured the level of Aβ_1‐40_ and Aβ_1‐42_ peptides secreted into the conditioned media by the COs (Figure 3A). Using a clinical‐grade Aβ peptide multiplex kit, we found that the APP^Swe/^WT COs produce and secrete significantly more Aβ_1‐40_ and Aβ_1‐42_, while maintaining a similar Aβ_1‐42_/Aβ_1‐40_ ratio as the WT/WT (1) COs—perhaps not surprising since this is a mutation in APP rather than in the processing enzymes (Figure 3B). On the other hand, the PSEN1^M146V^/WT mutant secreted relatively less Aβ_1‐40_ compared to Aβ_1‐42_, thus shifting the peptide products toward a significantly higher Aβ_1‐42_/Aβ_1‐40_ ratio (Figure 3A,B). Notably, following the relative secreted Aβ_1‐42_/Aβ_1‐40_ ratio in COs from 3 to 7 months of age, we found the APP^Swe/^WT maintained a stable Aβ_1‐42_/Aβ_1‐40_ ratio while the elevated PSEN1^M146V^/WT mutant's ratio progressively decreased over time (Figure S6A). This is remarkably the same progression numerous mass‐spectrometry‐validated studies have found in CSF of patients with fAD mutations compared to non‐carrier controls decades before symptom onset [83, 84, 85]. Isogenic set 2 revealed that PSEN1^ΔE9^/WT COs had increased Aβ_1‐42_ levels as well as an increased Aβ_1‐42_/Aβ_1‐40_ ratio, similar to PSEN1^M146V^/WT mutant COs. This ratio was also not significantly different between the two WT genotypes. Finally, Aβ_1‐38_ peptide measurements demonstrated more than a two‐fold reduction in the PSEN^M146V^/WT mutant compared to WT, but a moderate increase in the APP^Swe^/WT mutant (Figure S6B). This finding agrees with previous work on PSEN1 and could suggest an inverse relationship between the production of Aβ_1‐38_ and Aβ_1‐42_ [86, 87, 88]. Aβ_1‐38_ was below the detection limit in Isogenic Set 2. We identified sparse aggregates of Aβ in COs up to ∼4.5 months old by immunostaining, after which fAD COs began to exhibit a stronger amyloid signal (Figure S6C). At advanced stages (9 months), fAD COS display a stronger amyloid signal, as revealed by Amytracker co‐localizing with Aβ_1‐42_ (Figure S6D) [89].

Given the strong link between Aβ and subsequent phospho‐Tau pathologies, we employed the AT8 monoclonal antibody (recognizing Tau phosphorylated at Ser202 and Thr205), and commonly used to label pathologic paired helical filaments (PHF) via IF to probe the COs for pathological phosphorylated Tau (pTau) [90]. While both WT/WT genotypes had insignificantly different PHF‐positive signal, we observed significantly increased PHF‐positive staining in all the AD‐associated mutants compared to their isogenic WT controls as early as 3 months of age (Figure 3C). Given this robust phenotype, we further investigated whether the COs recapitulated a progressive accumulation of clinically‐relevant pTau as found in the human AD brain [91].

We decided to characterize in the COs the two most clinically relevant pTau modifications, phospho‐Thr217 (pT217) and phospho‐Thr181 (pT181), which most closely correlate with disease progression in CSF and plasma (particularly pT217) [92, 93, 94, 95]. Although we attempted these measurements in conditioned medium, their abundance levels were found to be too low for detection via western blotting. However, we found both of these pTau species to be orders of magnitude more abundant in CO lysates. To control for the known limitation of “pan‐Tau” or “total Tau” antibodies lacking sensitivity to many phosphorylated forms of Tau due to phosphorylation partially inhibiting their epitope binding [96, 97], we initially focused our Tau quantification on the major band at the predicted monomeric molecular weight, denoted mTau hereafter (Figure S7A).

Notably, we consistently found the appearance of specific high‐molecular‐weight (HMW) pTau^+^ bands, generally most abundant in the PSEN1^M146V^/WT mutant COs (Figure 3D). We set out to rule out non‐specific antibody binding. Using different antibodies, we found that apart from the expected monomeric bands around 50–55 kDa, the most abundant pT217^+^ band appeared at a MW consistent with dimers, at around 105 kDa (Figure S7A–C); while pT181 bands would often show a band at around 155 kDa, suggestive of trimers (albeit often too weak to quantify) (Figure S7D‐F). A limited number of previous clinical and animal studies have reported “low‐n oligomeric” and “multimeric” pTau species detected via WB at approximately these MWs [98, 99, 100]. Given the stringent reducing and solvating conditions of SDS‐PAGE, the detection of these HMW species would suggest that they may be SDS‐resistant and/or cross‐linked in nature, suggesting these phospho‐sites may be associated with oligomerization. To definitely validate these multimeric HMW bands, we performed immunoprecipitation (IP) enrichment of fresh PSEN1^M146V^/WT CO lysates and analyzed the protein content at these HMW bands via mass spectrometry (MS). Both pT181 and pT217 were identified, and WB highlighted that immunoprecipitated Tau contained significantly more pT181 than pT217 (Figure S7G). Importantly, despite the HMW species not immunostaining with pan‐Tau antibody (Figure S7A,D), MS confirmed that the HMW bands contained pTau species, specificity by identifying microtubule‐associated protein Tau (MAPT) in the IP‐enriched bands at ∼50, ∼105, and ∼155 kDa (Figure S7H, Experimental Section). Thus, we quantified the respective HMW species independently of the monomeric ones.

Accordingly, we characterized Tau in CO lysates at the 1.5, 3, and 6‐month timepoints. Consistent with the immunofluorescence (IF) data, we found mTau to be significantly elevated in the PSEN1^M146V^/WT mutant COs at the 1.5‐month timepoint (Figure 3D,E). PSEN1 mutations have been associated with accelerated neural development [19], which may help explain this finding. However, we found that mTau progressively decreased in these PSEN1^M146V^/WT COs over time, falling significantly lower at the 3‐month timepoint, and further decreasing at later stages and below half of WT levels by 6 months of age (Figure 3E,E’)— possibly indicating the conversion of mTau into oligomers by this time point, thereby reflecting the disease process. Similarly, the APP^Swe^/WT COs also demonstrated significantly decreasing mTau compared to WT COs after 3 months in culture, albeit with a less dramatic decline than the PSEN1^M146V^/WT COs. This loss of mTau is also consistent with the notion that the AD COs exhibit microtubule destabilization, characteristic of the neurodegenerative process [101].

Strikingly, the relative abundance of monomeric and HMW pT217 species manifested nearly opposite trends to one another in the AD COs compared to their isogenic WT controls (Figure 3F,G). Early on, at 1.5 months of age, the PSEN1^M146V^/WT COs displayed significantly increased monomeric pT217, while there were no detectable differences in HMW pT217. As the organoids aged, this pattern shifted: monomeric pT217 levels decreased in this mutant by 3 months, while the HMW pT217 levels trended higher, reaching pronounced fold‐changes by 6 months. Notably, this shift was even clearer in the second isogenic set's PSEN1^ΔE9/^WT COs. While they maintained relatively stable monomeric Tau levels (Figure S7I), these COs changed from only exhibiting significantly higher monomeric pT217 levels at 1.5‐month timepoint, to developing significantly elevated HMW pT217 levels as monomeric pT217 declined (Figure S7J,K). A similar pattern was observed in the pT181 species. At 1.5 months, the PSEN1^M146V^/WT COs had elevated levels of monomeric pT181 without significant HMW bands (Figure 3H). Over time, however, we observed the monomeric pT181 levels losing their elevated levels in the monomeric pool while HMW pT181 progressively increased at the 3‐ and 6‐month timepoints (Figure 3H,I). These results highlight that PSEN1 COs in particular progressively accumulate HMW pTau species. Furthermore, these data suggest the fAD CO model may mechanistically capture the time course of the development of pT217 and pT181 oligomer‐like HMW species.

Finally, to further corroborate the extent to which the COs develop oligomeric pTau species, we ran a NATIVE PAGE under reducing conditions at ∼4‐month timepoint (Figure S7L). Probing for pT181 revealed abundant smeared bands at ∼480–720 kDa apparent MW in both mutant COs, most prominently in PSEN1^M146V^/WT.

### 2.4 AD COs Recapitulate Signatures of Neurodegeneration

To test the hypothesis that the relative progressive loss of mTau and/or monomeric pTau at later time points could be associated with neuronal loss, we characterized cell death in the COs at the 1.5 and 3 month timepoints via TUNEL (terminal deoxynucleotidyl transferase dUTP nick end labeling) staining (Figure S8A,B) [102]. We complemented this approach by counting nuclei with condensed chromatin to assess apoptotic cell death (Figure S8C) [103, 104]. We found the AD CO mutants of both genetic backgrounds to have a significantly elevated number of apoptotic cells compared to their isogenic WT COs by the 3 month‐timepoint. Importantly, both backgrounds of WT isogenic COs showed consistent and stable numbers: they exhibited neither significant increases in cell death between timepoints (Figure S8C, *left*), nor any differences when compared to each other at either timepoint (Figure S8C, *right*). This consistency demonstrates our results are robust and independent of the COs’ genetic background. This finding suggests cells in the mutant AD COs may either have developed increased vulnerability during that time or other emergent mechanisms have led to increased cell death within the mutant COs only.

Prior work has correlated the increase in four‐repeat (4R) tau to coincide with neuronal damage and death [105, 106, 107]. Thus, we investigated the presence of three‐repeat (3R) and 4R Tau isoforms in the COs across time (Figure S8D). Our results indicate that 4R Tau is not detectably expressed before the 3‐month timepoint and progressively increases with organoid age up to a relatively low abundance (< 5%) compared to 3R Tau (Figure S8E). Interestingly, while the isogenic WT COs retain about the same proportion of 3R and 4R Tau from the 3 to the 6‐month timepoint, both, PSEN^M146V^/WT and APP^Swe^/WT AD COs produce significantly more 4R Tau leading to a lower 3R/4R ratio by the 6‐month timepoint (Figure S8F). Nonetheless, the detection of increased cell death in the AD COs at the 3‐month timepoint occurs months before this AD‐specific accumulation of 4R Tau, suggesting that cell death stems from a different mechanistic basis.

Remarkably, the 4R Tau (but not the 3R isoform) antibody bound to the HMW bands by the 6‐month timepoint in only the AD COs (Figure S8G). Interestingly, the samples with the strongest HMW bands corresponded with those with the weakest monomeric Tau signals, further supporting the hypothesis of age‐correlated shifting of mTau into HMW bands.

### 2.5 AD COs Have an Abnormally High Basal Level of Autophagosomes

**FIGURE 4:**
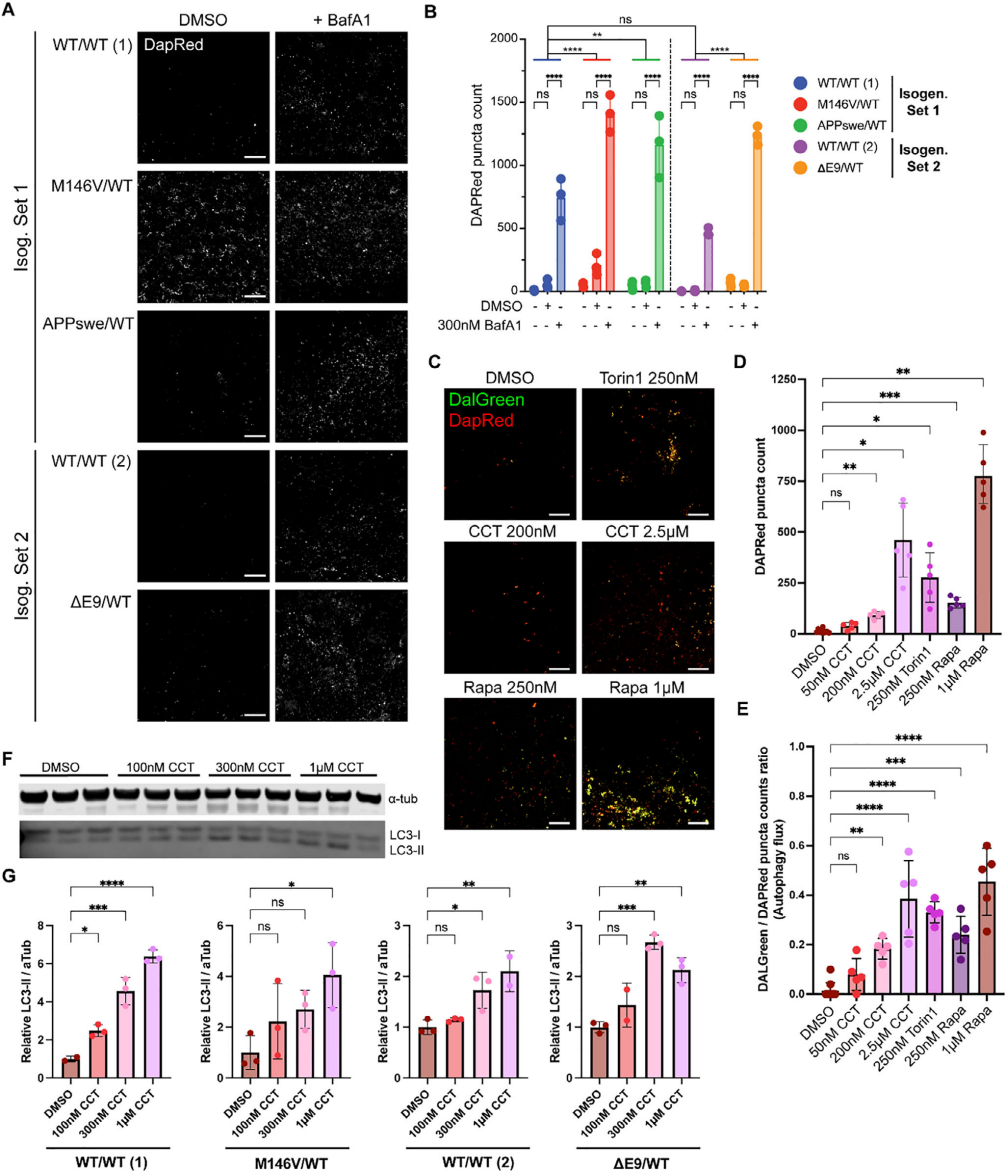
Autophagy can be efficiently activated in the AD COs. (A) Autophagosome staining via DAPRed live staining of COs in Neural Media (N. Media), with an overnight treatment of DMSO or 300 nm of Bafilomycin A1 (BafA1). Scale bar, 50 µm. (B) Quantification of average DAPRed+ puncta count per image. *n* = 3 COs per condition. Analysis by 2‐way ANOVA with Dunnett's and Sidak's post‐hoc tests for inter‐ and intra‐genotype comparisons, respectively. (C) Representative immunofluorescence (IF) staining images of DALGreen and DAPRed signal in COs (pictured here: PSEN1^ΔE9^/WT) after a 24 h treatment with either different concentrations of autophagy activators Rapamycin (Rapa), CCT020312 (CCT), Torin 1 (Torin1), or vehicle. Scale bar, 50 µm. (D and E) Quantification of the average IF DAPRed+ puncta count per image (D) and DALGreen to DAPRed puncta counts ratio (E). *n* = 5–10 images from 3–4 COs per condition from 1–2 independent experiments. (F) Representative WB of the dose‐dependent LC3‐II abundance increase in COs (pictured here: WT/WT (2)) after a 24 h treatment with CCT. (G) WB Quantification of the relative LC3‐II abundance in 24 h treated COs, normalized to their respective DMSO values. Data are mean ± SD. Analyses by ANOVA with Dunnett's post‐hoc test.

A decrease in autophagic flux has been linked to AD pathophysiology, and activation of autophagy has been suggested as a potential therapeutic target for neurodegenerative diseases like AD [46, 48, 49, 50]. We therefore characterized the basal autophagic flux state in the COs. We performed live imaging utilizing commercially available DAPRed (Dojindo) [108]. This amphiphilic dye integrates into the double membrane of autophagosomes. DAPRed fluoresces upon hydrophobic membrane incorporation, labeling autophagosomes and autolysosomes. We compared the number of DAPRed puncta in the vehicle control (DMSO) relative to bafilomycin A1 (BafA1) exposure, which prevents autophagosomes from fusing with lysosomes to create an acidic degradation compartment in the autolysosome. The BafA1 condition not only ensures that endo‐lysosomal flux is not blocked—as otherwise there would be no difference in the number of autophagosomes accumulating after BafA1 exposure—but it can also be used to estimate the number of autophagosomes processed through the lysosomal pathway. We found an increased number of autophagosomes converted to autolysosomes in all the AD mutants (Figure 4A,B; cf. difference between +BafA1 across genotypes). Despite having more autophagosome load, the lysosomal flux system was not clogged or impaired, as demonstrated by the BafA1 exposure significantly increasing the number of autophagosomes (Figure 4A,B; cf. difference between DMSO and BafA1 condition). This suggests an increase in basal macroautophagic flux in fAD, likely compensating for aberrant proteostasis early in the course of disease, as studied in our CO model system.

### 2.6 Increases in AD CO Disease‐Correlated pTau Are Reversed by the Novel Lysosomal Flux Activator, CCT020312

Given the observed elevated autophagosome load in the fAD COs, we posited that pharmacological activation of lysosomal flux could enhance macroautophagy efficiency, thereby reducing the levels of pathogenic protein aggregates associated with the disease. Recently, compound CCT020312 (denoted CCT hereafter) was identified from a cell‐based high‐content screen and validated as an mTOR inhibitor‐independent lysosomal flux activator [109]. This was of particular interest to us, given mTOR inhibition's unwanted side effects, such as immunosuppression. CCT was previously shown to dose‐dependently reduce insoluble pTau levels in a hiPSC‐derived 2D neuronal model of Tauopathy and decrease Aβ peptides in direct‐differentiated neurons [109]. The vulnerability of the 2D neuronal cultures of Tau‐P301L neurons to stressors like rotenone and Aβ also exhibited substantially less cytotoxicity with CCT pre‐treatment. Thus, we hypothesized that autophagy activators like rapamycin—an mTOR inhibitor [48]—and CCT could potentially reverse the clinically relevant phenotypes of pTau accumulation that we identified in our fAD COs.

Next, we assessed the ability of known mTOR‐dependent autophagy activators, like Torin 1 and rapamycin (Rapa), or the mTOR‐independent drug CCT to increase lysosomal flux in the AD COs at comparable concentrations to other in vitro models [110]. To rigorously assess lysosomal flux, we made use of DAPRed and DALGreen (Dojindo) in conjunction. While both DAPRed and DALGreen integrate into autophagosomes, the fluorescence of DALGreen is greatly enhanced in acidic environments, thus labeling autolysosomes where the pH is lower. This enables the quantification of the relative ratio of mature autolysosomes and early autophagosomes; that is, live assessment of flux from autophagosome formation to post‐fusion autolysosome maturation. We observed a significant dose‐dependent increase in both autophagosomes (DAPRed puncta) and autophagic flux (DALGreen/DAPRed puncta ratio) after 24 h treatment with Torin 1, Rapa, or CCT (Figure 4C–E). Our assessments agreed that 250 nm Torin 1, and doses as low as 250 nm Rapa or 200 nm CCT were sufficient to significantly increase autophagic flux in the COs after 24 h.

We further validated the effect of these autophagy activators via LC3‐II quantification through western blotting (Figure 4F). We found that, in general, concentrations as low as 100 nM CCT led to increased LC3‐II accumulation in the WT/WT (1) COs, but there was some variability depending on the CO genotype. (Figure 4G). Overall, the live dye and LC3‐II assessments suggest concentrations of ∼300 to 1 µm CCT are required to robustly activate autophagy in the COs regardless of the genotype.

Next, we set out to study the therapeutic effects of chronically treating the COs with autophagy activators, employing various dosing regimens. We began with mTOR‐dependent Rapa using two different treatment regimens of 2 to 4 weeks based on prior similar treatments on a different hiPSC‐derived 2D neuronal model [109]. Specifically, we tested dosing twice with 250 nm Rapa at day 0 and day 13 over the span of 2 weeks, as well as a more frequent lower dose regimen of 125 nm Rapa every 4 days over the span of 4 weeks, after which we lysed the COs to assess the effect on pTau (Figure S9A, S9B). We found that both regimens yielded nearly identical results and thus were combined for analysis. The Rapa treatment increased levels of mTau in the PSEN1^M146V^/WT COs (Figure S9C), while significantly decreasing levels of HMW pT217 in all COs (Figure S9D). Rapa also decreased pT181 in the APP^Swe/^WT COs (Figure S9E). Overall, we found that rapamycin was effective at reducing pTau compared to vehicle.

**FIGURE 5:**
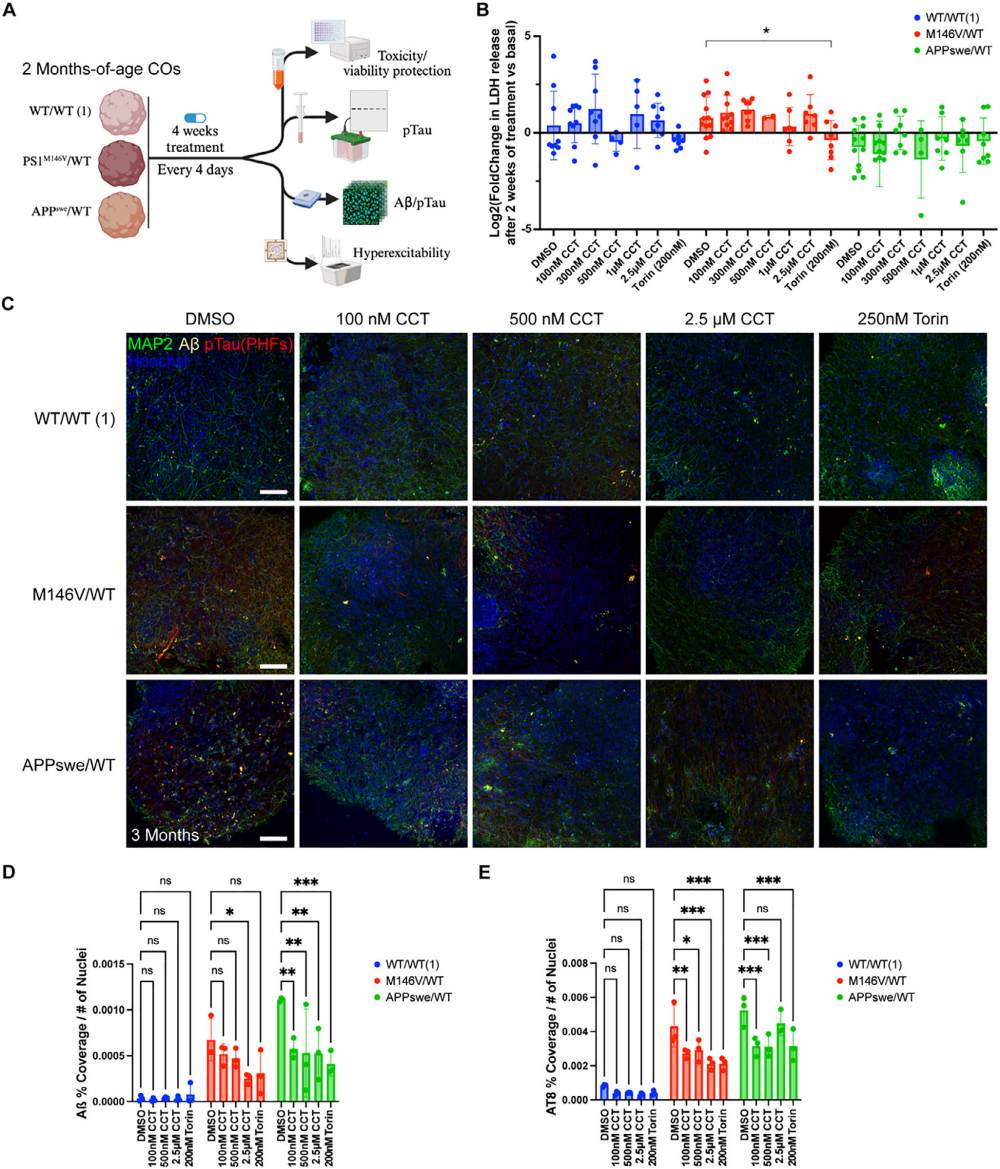
Chronic mTOR‐independent autophagy activation reduces AD‐associated Aβ and pTau aggregates, and hyperexcitability. (A) Diagram describing the experimental set‐up of the 4‐week‐long treatment of 2 months‐of‐age COs (3‐month timepoint by the end of treatment). (B) Measured change in the conditioned media levels of LDH (U mL^−1^) at the end of treatment (4 weeks), compared to their basal levels at treatment day 0 (T0) (LDH at end of treatment minus LDH at T0). Data were log‐transformed to achieve normality. (C) Representative immunofluorescence (IF) images of the COs at the end of the treatment period. Scale bar, 100 µm. (D) Quantification of the Aβ percent coverage normalized to nuclei number of the COs. *n* = 3 COs per condition. (E) Quantification of the Paired Helical Filament (PHF) aggregated pTau (AT8^+^) percent coverage normalized to nuclei number of the COs. *n* = 3 COs per condition. Data are mean ± SD. Analyses by ANOVA with Dunnett's post‐hoc test.

Similarly, we aimed to determine whether chronic treatment with the mTOR inhibition‐independent autophagy activator, CCT, could replicate the decrease in tau pathology observed with rapamycin treatment. This comparison was particularly important because inhibition of mTOR has significant side effects that may not be tolerated well clinically [48, 111], motivating our development of an mTOR‐independent mechanism to activate autophagy. We therefore conducted a series of experiments to comprehensively evaluate the effects of CCT on the COs (Figure 5A). For a 4‐week‐long treatment period, multiple batches of COs starting at 2‐month timepoint were dosed every 4 days with CCT or vehicle prepared in fresh neural medium at every other medium change. As a positive control to complement the rapamycin findings, we used a second mTOR‐dependent activator of autophagy, Torin 1, at a concentration of 200 nm.

Potential toxicity due to the long‐term dosing of the drugs was tracked by measuring LDH release in conditioned media from pre‐treatment day zero (T0) to the mid‐, and endpoint every two weeks. We did not find any dose‐dependent toxicity in any of the CO genotypes, although, notably, we observed relatively lower levels of LDH released in PSEN1^M146V^/WT COs treated with 200 nm Torin 1 (Figure 5B). Next, we assessed immunohistologically the COs for Aβ and PHF pTau pathology (Figure 5C). We not only confirmed once again elevated levels of Aβ and AT8+ signal in both PSEN1^M146V^/WT and APP^Swe^/WT COs compared to WT, but we also found a clear, dose‐dependent decrease in both Aβ (Figure 5D) and AT8+ PHF signal with CCT treatment (Figure 5E). This demonstrates the ability of both CCT and Torin 1 (used here as a positive control) to directly reduce AD pathology.

Previously, CCT has been regarded as an activator of the PERK arm of the Unfolded Protein Response (UPR) [112, 113, 114, 115, 116, 117]. Recent work, however, from our group has shown that CCT enhances lysosomal flux at lower concentrations than those required to activate PERK [109]. To verify this was the case here in our human CO model, we treated for both WT/WT and PSEN1^M146V^/WT mutant COs for one week with CCT at concentrations ranging from 100 nm to 25 µm. COs were then lysed and probed for Activating Transcription Factor 4 (ATF4), a canonical downstream target of PERK/UPR activation [118, 119]. We found that CCT did not have any effect on ATF4 translation below 10 µm (Figure S10A). As a positive control, 500 nm Thapsigargin, a well‐validated UPR activator [120, 121], induced a consistent increase in ATF4 signal in both CO genotypes. Critically, pretreatment or co‐treatment of CCT with integrated stress response inhibitor (ISRIB), a small molecule acting downstream of PERK activation and a potent counteractor of its effects [122], had no effect on the ability of CCT to reduce PHF load in AD COs (Figure S10B). Thus, we conclude the therapeutic effects of CCT observed here at concentrations ≤2.5 µm are independent of its ability to activate PERK, which is only seen at higher concentrations.

Following up on our aforementioned finding of pTau accumulation in the PSEN1^M146V^/WT mutant, 4‐week CCT‐treated CO lysates were also biochemically assessed for pTau changes (Figure S11A). The results showed that the mTau levels were not significantly affected by lysosomal flux activator treatment in either genotype at this timepoint (Figure S11B). In contrast, PSEN1^M146V^/WT COs manifest a significant, dose‐dependent reduction in pT181 after treatment with 1–2.5 µm CCT (Figure S11C). Torin 1 (250 nm) appeared to have a similar, albeit somewhat less effect, but, unlike CCT, is also a mTOR inhibitor at this or higher concentrations, which would produce substantial clinical side effects. Finally, CCT had no statistically significant effect on pT217 levels (Figure S11D; 100 kDa band). In conjunction with our findings that pT217 at this MW only accumulates in the PSEN1^M146V^/WT COs at later timepoints, these results are consistent with the notion that the mTOR‐independent lysosomal flux activation by CCT may preferentially target abnormally elevated pTau species.

### 2.7 Chronic Treatment With Lysosomal Flux Activator, CCT, Rescues mTau, Monomeric pTau, and Synapses in PSEN1^M146V^/WT COs

**FIGURE 6:**
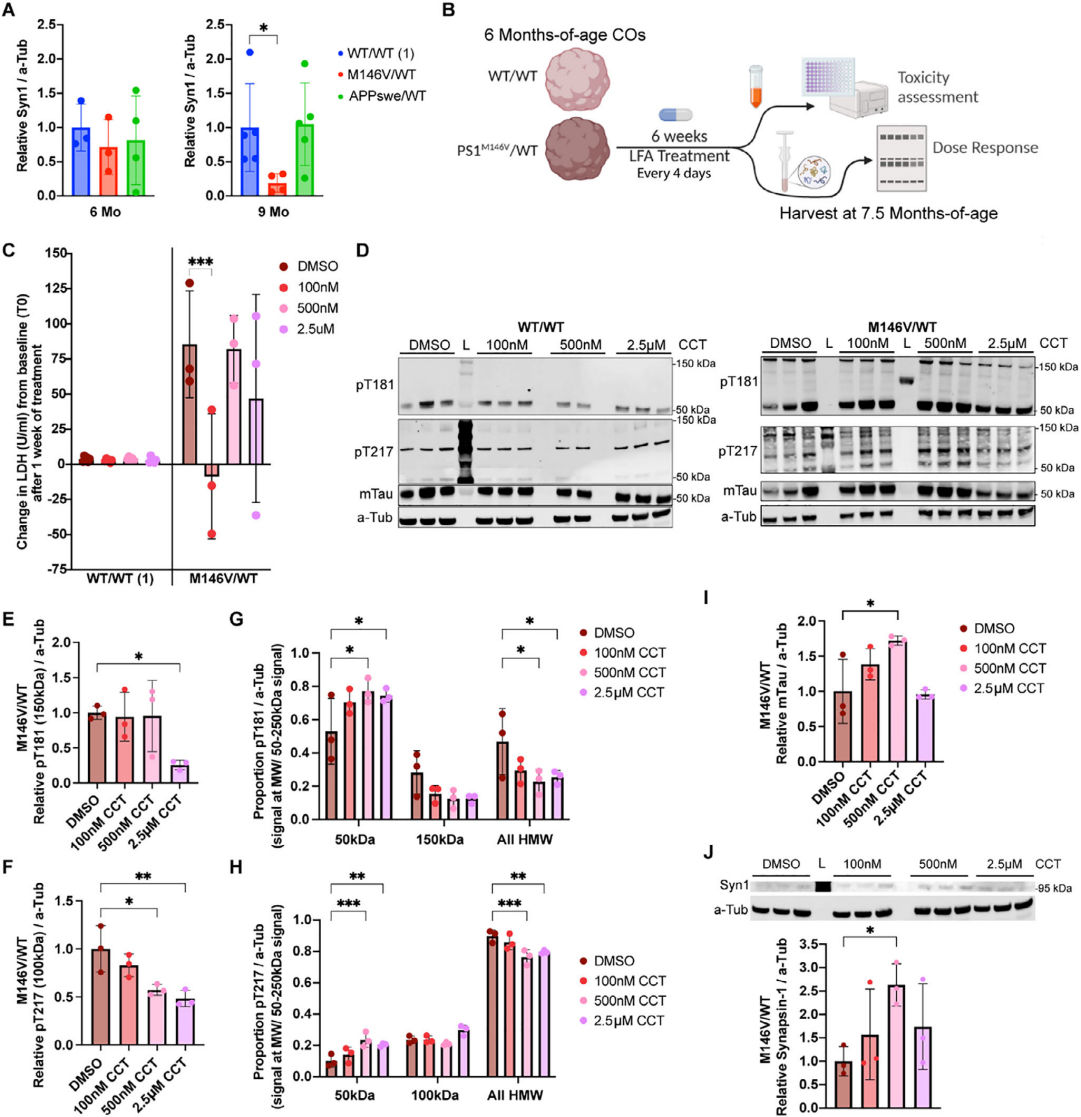
Chronic mTOR‐independent autophagy activation with CCT rescues mTau (monomeric pTau) and synapses in the M146V mutant. (A) Immunoblot quantification of Synapsin‐I (SynI normalized to a‐Tub) in CO lysates at 6 and 9 months‐of‐age. *n* = 3–4 COs per genotype. For each timepoint, SynI levels are shown relative to WT/WT (isogenic set 1) levels. Unpaired t test with Welch's correction with post‐hoc FDR‐adjusted *P*‐values: *p* = 0.0461 (M146V/WT vs. WT/WT(1)), *p* = 0.9082 (APP^Swe^/WT vs. WT/WT(1)). (B) Schematic describing the treatment of 6 months‐of‐age COs with CCT and its assessment at the end of a 6‐week treatment regimen (harvested at 7.5 month‐timepoint). (C) Quantification of LDH in conditioned media after 1 week of treatment with DMSO vehicle vs. 100 nm, 500 nm, or 2.5 µm CCT for WT/WT (1) and PSEN1^M146V^/WT COs. *n* = 5–6 COs per condition for WT/WT (1); *n* = 3 COs per condition for PSEN1^M146V^/WT. (D) Representative immunoblots of WT/WT and PSEN1^M146V^/WT CO lysates for pT217, pT181, mTau after 6‐week treatment. L = molecular weight ladder. (E, F) Quantification of phosphorylated Tau T181 (pT181) (E) and phosphorylated Tau T217 (pT217) (F) normalized to ɑ‐tubulin (a‐Tub) in PSEN1^M146V^/WT CO lysates after 6‐week treatment relative to DMSO. (G, H) Quantification of the proportion of pT181 (G) and pT217 (H) bands (normalized to a‐Tub) localized at various molecular weights (MW); specifically, at the monomeric band (50 kDa), major validated high‐molecular‐weight (HMW) bands (150 kDa), or all HMW signals (75–250 kDa); in PSEN1^M146V^/WT CO lysates after 6‐week treatment relative to DMSO. (I) Quantification of monomeric Tau (mTau normalized to a‐Tub) in PSEN1^M146V^/WT CO lysates after 6‐week treatment relative to DMSO. (J) Representative immunoblot of DMSO vehicle‐ vs. CCT‐treated PSEN1^M146V^/WT CO lysates for Synapsin‐I (SynI) and a‐Tub after 6‐week treatment (*left*) with quantification (*right*). L = molecular weight ladder. *n* = 3 COs per condition. Data are mean ± SD. Analyses by ANOVA with Dunnett's post‐hoc test.

We identified between the 6‐month and 9‐month timepoints a period during which the PSEN1^M146V^/WT AD COs appear to lose synaptic density, as evidenced by our biochemical assessment of presynaptic marker Synapsin‐I (Figure 6A) and consistent with our prior electrophysiological and histological assessments on similar AD COs [44, 45]. This observation, combined with assessments of cell death and mTau reduction, suggested the PSEN1^M146V^/WT mutant COs underwent progressive synaptic loss typical of neurodegeneration compared to isogenic WT and to a greater degree than the APP^Swe^/WT COs. To assess whether CCT could affect this pathological progression, we conducted a 6‐week treatment regimen initiated in COs at 6 months of age and ending at the 7.5‐month timepoint (Figure 6B). To assess viability, we measured LDH levels in conditioned media at the pre‐treatment, 1‐week, 2‐week, and 4‐week timepoints (Figure 6C and Figure S12A). Although all conditions showed some increase in LDH release after the first week, the PSEN1^M146V^/WT COs manifested a significantly higher increase in LDH released, suggesting their heightened vulnerability. Notably, CCT treatment did not exhibit toxicity compared to DMSO controls at any timepoint. On the contrary, we observed a significant reduction in LDH release relative to pre‐treatment levels in the PSEN1^M146V^/WT COs treated with 100 nm CCT as soon as one week after treatment (Figure 6C).

When monitoring pTau, we identified a significant decrease in pTau after 3 weeks into the treatment (Figure S12B). After the 6 weeks of treatment, the HMW bands of pTau became significantly more complex and developed smearing in the PSEN1^M146V^/WT CO lysates (Figure 6D, *right*), resembling more closely the signature of oligomeric pTau pathology in human AD patient brains [98]. We found that CCT dose‐dependently decreased the major HMW pT181 and pT217 bands (Figure 6E–H). Consistent with our hypothesis of CCT‐activated autophagy targeting abnormally elevated pTau, 500 nm and 2.5 µm CCT treatment not only decreased the abundance of the major HMW pT181 and pT217 bands (Figure 6E,F), but also simultaneously shifted the relative proportion of HMW‐to‐monomeric pTau species, as demonstrated by the increase in monomeric pT217 and pT181 bands (Figure 6G,H). These results suggest CCT‐activated autophagy may facilitate the digestion of SDS‐ insoluble oligomers into SDS‐soluble monomers.

CCT was also found to dose‐dependently increase the abundance of mTau, reaching significance at the 500 nm dose (Figure 6I). This is an important recovery from our finding that mTau dramatically decreases in the PSEN1^M146V^/WT COs beyond the 4.5‐month timepoint in the absence of treatment. Finally, in PSEN1^M146V^/WT COs we found that 500 nm CCT significantly increased the levels of Synapsin‐I compared to DMSO vehicle (Figure 6J), suggesting a beneficial effect on synapse number. Taken together, these results nominate CCT as a potential therapeutic worthy of further testing in a human disease context, at least for PSEN1^M146V^/WT mutations.

### 2.8 Pathologic Neuronal Hyperexcitability in the PSEN1^M146V^/WT AD COs Is Corrected by the Lysosomal Flux Activator, CCT

**FIGURE 7:**
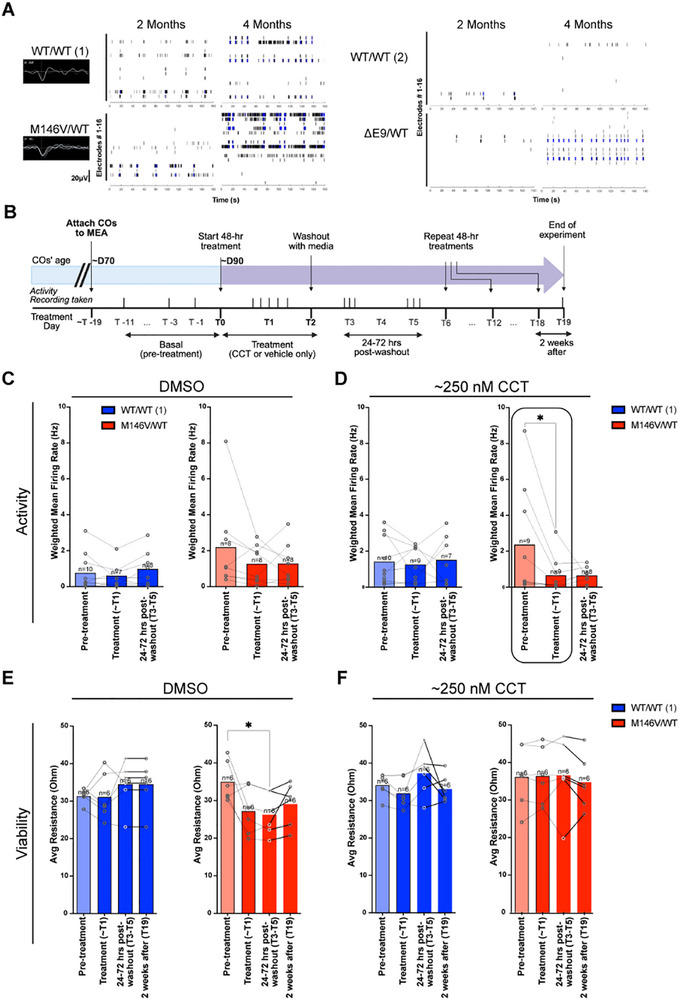
AD COs display neuronal hyperexcitability that is dose‐dependently reduced by CCT. (A) Representative raster plots and mean firing rate (Hz) quantification from MEA recordings over the span of 180 s of COs from isogenic sets 1 and 2 at the 2 and 4‐month timepoints. (B) Schematic describing treatment with CCT and activity recording of isogenic set 1 COs. (C, D) Quantification of WT/WT and PSEN1^M146V^/WT CO neuronal activity, as calculated by the weighted mean firing rate (Hz) during basal, treatment, and recovery periods (the latter obtained 1–3 days after washout) for DMSO and ∼250 nm CCT. The number of COs recorded indicated above bars in the figure. Significant difference highlighted by oval. (E, F) Quantification of WT/WT and PSEN1^M146V^/WT CO viability, as reflected by mean electrode impedance (measured as resistance in kΩ) during basal, treatment, and washout periods for DMSO and ∼250 nm CCT. Data are mean ± SD. The number of individual COs recorded (from two independent experiments) is indicated above bars in the figure. Analysis by Mixed‐Effect Model (RELM) for matched repeated measures.

Hyperexcitability has been identified as an early and important contributor to the pathogenesis of AD, affecting Tau aggregation and synapse loss [44, 76, 123, 124, 125]. Using a microelectrode array (MEA) recording system (Axion Biosystems), we attached individual COs to single MEA wells to enable real‐time basal electrical activity readings. By impedance measurements, we verified that CO attachment to the MEA plate was not different between mutants and WT. By the ∼3–4 month timepoint, the PSEN1 CO mutants produced abnormally high levels of action potential firing and bursting, as evidence of hyperactivity (Figure 7A). This finding was in line with results we had previously quantified and reported (viz. Figure 7H in Ghatak et al. [44] and Figure 5c,e,f in Ghatak et al. [45]) showing hyperelectrical activity in PSEN1 mutant COs compared to isogenic WT COs. Here, we assessed whether CCT could correct hyperexcitability in the PSEN1^M146V^/WT COs.

After treating once every 6 days for a period of 3 weeks (Figure 7B, T0‐T19), we assessed the weighted mean firing rate and viability of the COs (see Experimental Section for definitions). We compared the effect of 200 and 300 nm CCT (hereafter ∼250 nm) to DMSO vehicle on PSEN1^M146V^/WT COs vs. their isogenic WT COs (Figure 7C,D). We found a decrease in the mean firing rate in PSEN1^M146V^/WT COs (circled data) but not WT/WT COs after CCT treatment. The decrease in mean firing rate persisted 24–72 h post‐washout (labeled T3–T5).

The impedance measurement of the MEA electrodes directly reflects the electrical resistance associated with organoid attachment and cell membrane integrity. Thus, these parameters are used as an index of organoid viability. Vehicle (DMSO)‐treated PSEN1^M146V^/WT COs manifested a significant drop in viability (impedance) compared to their basal levels by the washout period (T3‐T5); however, this drop in viability did not occur in the DMSO‐treated WT COs (Figure 7E). These data further support the conclusion that the AD COs manifest increased vulnerability compared to WT. Moreover, treatment with ∼250 nm CCT rescued the loss in impedance (reflecting viability) of the AD COs (Figure 7F). This added fact supports our interpretation that the decrease in hyperexcitability observed with CCT treatment in the PSEN1^M146V^/WT COs was not due to a decrease in cell viability. Taken together, the results are consistent with the notion that CCT decreased AD‐related hyperexcitability and loss of viability. Moreover, our findings suggest that the CO platform is a viable model for assessing the effects of autophagy activators like CCT in a human context in real time using relevant electrophysiological as well as biochemical readouts.

## 3 Discussion

In this study, we have leveraged hiPSC‐derived COs to create a scalable and consistent in vitro human model for fAD vs. isogenic controls, using it to focus on the earliest AD‐related pathology. We demonstrate this model succeeds in capturing both the hallmark increase in Aβ and phosphorylated Tau proteins with synaptic marker loss and cellular death, as well as the recapitulation of pathophysiologic neuronal hyperactivity observed in pre‐symptomatic PSEN1 mutant and APOE4 variant carriers via hippocampal fMRI, and early in human AD brains by electroencephalogram (EEG) [126, 127].

The AD COs developed here not only replicate the early molecular, transcriptomic, and synaptic abnormalities previously reported in clinical human AD patients [2, 75, 91, 98, 128, 129], but also reveal distinct molecular signatures depending on the familial AD mutations present. Specifically, we show the PSEN1 mutant COs developing the strongest phenotypes in most of our characterization, including elevated early levels and progressive accumulation of HMW pT217 and pT181, synaptic loss, and neuronal hyperexcitability. This suggests that mutations in the PSEN1 and APP genes drive unique aspects of AD pathology, which may help explain their differential associated clinical prognosis. Indeed, the PSEN1 mutants were the only ones that had an elevated Aβ_1‐42_/Aβ_1‐40_ ratio, which is considered a stronger diagnostic than just elevated Aβ_1‐42_ alone, which APP^Swe^/WT COs had [130]. PSEN1 mutations are also associated with significantly more aggressive early onset dementia with an age of onset of 24–55 years of age [40, 128], while the APP^Swe^/WT mutation is associated with a slower age of onset of 40–65 years of age [128]. The fact that the Aβ_1‐42_/Aβ_1‐40_ ratio also followed over time the same mutant‐specific progressive decline as reported in CSF clinical studies decades before symptom onset [83, 84, 85], suggests the CO model to be reproducing real progressive processes at an accelerated pace. Surprisingly, no significant accumulation of Aβ aggregates was necessary for the appearance of pTau pathology. This highlights the notion that these amyloid aggregates may be secondary consequences, with the Tau hyperphosphorylation‐drivers likely being earlier amyloid species‐dependent processes (e.g. soluble Aβ oligomers) [131, 132, 133].

The relative decrease in monomeric phosphorylated Tau was an intriguing finding in our study. Clinical studies on AD patients have found a significant reduction in the abundance of APP and Tau protein in CSF microvesicles [130]. We hypothesize the COs may be reproducing a process early in the disease etiology, by which specific pTau may be irreversibly oligomerizing, leading to its decrease in SDS‐soluble extraction. The formation of phosphorylated Tau dimer and higher‐order intermediate oligomers as they form Tau fibrils has been previously explored [134, 135, 136]. New evidence suggests these low‐order oligomers may be the most hazardous form of Tau oligomers [133, 137]. The field in general agreed that hiPSC‐derived neurons do not exhibit Tau multimerization [136, 138]. To our knowledge, we are the first to report validated low‐order oligomer reduction‐ and SDS‐resistant pTau oligomers in any in vitro model.

Interestingly, although the COs expressed relatively low levels of the 4R Tau isoform comparable to other published hiPSC‐based studies [105, 139], HMW species were detectable only by the 4R‐isoform Tau antibody. This suggests 4R Tau may be enriched in these pTau oligomers compared to 3R Tau, indicating the 4R isoform being more prone to HMW oligomerization, potentially explaining its differential pathology in disease [140, 141, 142]. Recently, AD patient‐derived soluble HMW Tau was found to drive abnormal neuronal bursting in mouse hippocampal neurons [143]. The fact that the COs exhibit both of these characteristics suggests they may be an accessible human model to study electrophysiologically‐relevant pathology lacking in other models. Additionally, our findings reveal different effects in excitatory neurons compared to inhibitory ones, which may explain why patients develop excitatory‐inhibitory imbalance during the disease and highlight potential new therapeutic targets in the transcriptomic signatures of these neuronal subtypes. Recent studies support this notion [79, 144, 145, 146].

A key limitation of this study is the inherent immaturity of hiPSC‐derived cerebral organoids (COs), which, despite displaying senescent signatures, cannot fully recapitulate the epigenetic age of adult brain cells. Organoid models have, however, previously been suggested to display aging‐related changes due to the stress of being in culture [27], and the accelerated development of AD pathology in COs compared to the human brain that we observed supports this concept. Such cellular stressors identified here, including the ferroptosis and senescence‐related factors (elevated p16, p21, and SASP genes) [73, 74] may effectively mimic age‐related processes that drive AD progression and pro‐inflammatory conditions [27, 147, 148, 149]. Nonetheless, microglia being absent in this model restricts its ability to reproduce inflammatory processes critical for protein aggregate clearance and inflammatory disease progression, so our findings may be limited to pre‐inflammatory, early stages of AD. In this regard, several of the abnormal neuronal cellular pathways we identified in AD COs, such as dysregulated RNA processing, have also been linked to persistent neuroinflammatory conditions [150, 151, 152]. Thus, the features identified by our study reveal disease‐relevant neuronal states and selective vulnerabilities that inflammation might subsequently affect. The limitation of our being able to look only at early disease features in the AD COs may, in fact, be of significant benefit in screening for potential therapeutic agents in early AD [153, 154].

We posit that the presence of cellular stressors and senescence signatures contributes to accelerating AD‐relevant processes in the COs, despite the potential presence of unrelated non‐physiologic stressors [27]. Furthermore, the 3D structure of organoids provides a more physiologically relevant microenvironment in a human context that supports the development of AD‐related pathologies, such as the Tau hyperphosphorylation and aggregation we observe, resembling the high‐molecular‐weight oligomers observed in human postmortem AD brain. This may explain the emergence of more complex functional abnormalities, such as the differential effect on interneurons, hyperexcitability, and cell death [154, 155, 156].

Our study demonstrates that chronic treatment with lysosomal flux/autophagy activators—including mTOR‐dependent agents like rapamycin and Torin 1, but also mTOR‐independent drugs like CCT020312—can reduce AD‐like phenotypes, including the accumulation of Aβ and pTau over a two to six‐week treatment period. We show that CCT activates autophagy without activating PERK or the UPR, and that this activity reduces aberrant HMW pTau species. Critically, autophagy activators not only reduced pathogenic protein burden but also preserved cellular viability and electrophysiological properties of the AD COs. Despite chronic, weeks‐long exposure to these therapeutic agents, COs did not show toxicity; in fact, treatment rescued synapses and prevented hyperexcitability and cell death. Correlated with this neuroprotective effect of CCT, we observed an increase in Tau monomers and a concomitant decrease in HMW pTau species, suggesting that the oligomers may drive pathologic toxicity rather than total pTau load. Given the growing evidence that autophagy activation can also reduce neuronal senescence and its potential involvement in neurodegeneration [157], these findings strengthen the evidence for autophagy modulation as a promising therapeutic avenue for AD and provide insights for dosing strategies in future clinical applications [46, 48, 49, 51, 111, 158, 159].

Finally, we showcase the use of real‐time MEA recordings in COs as a useful platform for preclinical drug screening and investigation of the potential therapeutic effectiveness of drugs. The generation of hiPSC‐derived COs from various additional AD‐related mutations or sporadic cases should also facilitate personalized medicine approaches to AD treatment.

## 4 Experimental Section

### 4.1 Resource Availability

#### 4.1.1 Lead Contact

Any additional information and/or requests should be directed to the lead contacts, slipton@scripps.edu, or jwk@scripps.edu.

#### 4.1.2 Materials Availability

Commercially available materials are described in the Key Resources Table (Table 1). The unique materials generated in this study are available from the lead contact(s) upon completing a Materials Transfer Agreement.

#### 4.1.3 Data and Code Availability

Single‐cell RNA‐seq data are deposited at GEO, accession number GSE301700.

No significant custom code was generated in this study, though the specific basic *R* scripts used are available upon request. Mass spectrometry data will be available upon request. Any additional information required to use or reanalyze the data reported in this study is available from the lead contact(s) upon request.

### 4.2 Experimental Model and Subject Details

#### 4.2.1 Human iPSC Lines

The use of human cells had the approval of the institutional review board associated with The Scripps Research Institute. We used the previously established and characterized [35, 55] isogenic heterozygous PSEN1^M146V^/WT and APP^Swe^/WT mutant hiPSCs, along with their WT 7889SA parental (WT/WT) line (termed isogenic set 1), obtained from the laboratory of Marc Tessier‐Lavigne (for mutant lines) and the New York Stem Cell Foundation Research Institute (for the WT/WT line). For an additional genetic background, we used the previously established [42, 61] PSEN1^ΔE9^/WT heterozygous hiPSC and its WT (WT/WT) parental line (termed isogenic set 2), originally obtained from the laboratory of Lawrence S.B. Goldstein at the University of California, San Diego. Both genetic backgrounds were of male sex. Pluripotency was also confirmed via immunolabeling with pluripotent cell markers NANOG, OCT4, and TRA (data not shown). Euploidy was confirmed via both G‐banding (WiCell) and qPCR (StemCell Technologies Kit), which showed no significant gain of karyotypic mosaicism in the hiPSCs used in this study.

### 4.3 Method Details

#### 4.3.1 hiPSC Maintenance and Quality Controls

The hiPSC sets were maintained in parallel on vitronectin‐coated (Thermo Fisher) plates with the culture medium mTeSR plus (StemCell Technologies) being replaced every 24 h. The hiPSCs were carefully cultivated, morphologically abnormal cells were manually removed when needed, and cells were passaged via non‐enzymatic colony dissociation with ReleaSR (Thermo Fisher) every 5–6 days. The cells were routinely checked for mycoplasma, confirmed to remain contamination‐free, and maintained for no more than 13 passages at a time.

#### 4.3.2 Cerebrocortical Organoid (CO) Culture

To make the COs, an optimized protocol based on Sergiu Paşca's method was developed [110]. hiPSCs between 75% and 90% confluence were first dissociated with Accutase into a single‐cell suspension and seeded into an Aggrewell plate at a 4 million cells per well density in mTeSR plus media supplemented with a *CEPT* cocktail [160, 161, 162] (50 nm Chroman I, 5 µm Emricasan, 1x Polyamine supplement, and 0.7 µm Trans‐ISRIB) to form embryoid bodies (EBs) for 24 h in an 5% CO_2_ incubator at 37°C. EBs were then transferred to ultra‐low attachment 6‐well plates (Corning), shaking in an orbital shaker at 60 RPM. They were maintained in a 50/50 mixture of Gibco's Essential 6 medium and hESC medium (DMEM‐F12, 20% Knockout Serum Replacement, 1% Glutamax, 1% MEM‐Non‐essential amino acids, and 0.1 mm 2‐mercaptoethanol) [12, 63], supplemented with 2.5 µm Dorsomorphin, 10 µm SB‐431542, and 2.5 µm XAV‐939 for 6 days. The medium was then replaced with Neural Media‐A supplemented with 20 ng mL^−1^ of EGF_2_ and 20 ng mL^−1^ FGF_2_. From day 25 to 43, the organoids underwent maturation through replacing the supplements with 20 ng mL^−1^ BDNF and 20 ng mL^−1^ NT‐3; after which they were maintained in Neural media‐A until harvest. Importantly, around day 35, the organoids were transferred to ultra‐low attachment 10 cm dishes and kept shaking at an orbital shaker tilted at a 3.5° angle. The COs were monitored and split as needed to maintain consistent media consumption/acidification. CO cultures were tested for mycoplasma every 1–2 months.

#### 4.3.3 CO Brightfield Imaging

Transmitted light images of live representative COs were taken via an EVOS M5000 Microscope (Invitrogen) with a 2x or 4x objective and phase ring on the brightfield setting. Light intensity/exposure was adjusted to maximize the images’ contrast each imaging day independently, but maintained constant across conditions within each day. CO size (area) quantification was done via Fiji with the Cell Magic Wand plugin.

#### 4.3.4 Immunohistology of COs

Cerebrocortical organoids were washed 2x in PBS and fixed in a cold solution of 4% PFA in PBS overnight at 4°C. Organoids were then washed in a 300 mm glycine solution to neutralize the remaining PFA and washed 2x in PBS for 15 min each before being dehydrated through progressive equilibration in 15% and 30% sucrose in PBS overnight. Organoids were then embedded in tissue‐freezing medium (General Data) in a cryo‐mold and flash frozen in liquid nitrogen–cooled isopentane and stored at −80°C until processing. Fluorescence immunohistochemistry was performed on 20 µm cryostat‐sliced samples using standard protocols. Briefly, blocking and permeabilization were done with 3% BSA + 0.3% Tx‐100 for 10 min at RT, after which samples were incubated overnight at 4°C with primary antibodies diluted in the same blocking buffer (details in Table 1). Then, samples were washed with PBS and incubated with Alexa Fluor‐labeled secondary antibodies and Hoechst nuclei stain. Finally, samples were washed three times in PBS, coverslipped with Dako Fluorescence Mounting Medium, and sealed. Secondary antibody‐only controls were carried out as negative controls for each experiment. A Nikon C2 confocal microscope was used to perform the imaging, acquiring at least 3 Z‐stack captures in randomly selected fields for each replicate condition.

#### 4.3.5 Western Blot Characterization

Cerebral organoids at 1.5‐, 3‐, and 6‐month timepoints were washed 3x in PBS and then homogenized in protease and phosphatase inhibitor cocktail‐supplemented RIPA or 2% SDS lysis buffer (2% SDS in 150 mm Tris‐HCl, 7.6 pH). Results did not vary significantly based on the choice of lysis buffer. To ensure a complete protein extraction, after homogenizing the COs, the samples were directly pulse ultrasonicated (∼24 s) before centrifuging at 20 600 *g* for 20–30 min. Supernatant was saved into a new tube, and protein was quantified and normalized using Pierce BCA assay (Thermo Fisher). Next, SDS‐PAGE was conducted –using SDS‐free Laemmli sample buffer– with Bolt Bis‐Tris 4%–12% gradient polyacrylamide gels (Thermo Fisher). Gel was washed in 20% EtOH for 20 min, washed in 10% MeOH Transfer Buffer for 10 min, and then dry transferred onto PVDF membranes using an iBlot 3 Western Blot Transfer Device (Thermo Fisher). The membrane was then washed with water and stained with Ponceau S stain as a protein loading and transfer control. Then, the membrane proceeded with washing with TBST, blocking with Li‐Cor TBS blocking buffer, and incubating overnight at 4°C with primary antibodies diluted in the blocking buffer with 0.02% TWEEN‐20 (details in Table 1). Membranes were then washed 3x with TBST before incubating with secondary antibodies (1:10 K) for 1–2 h at RT. After washing 3x with TBST, membranes were scanned in an Odyssey CLx scanner (LI‐COR). After washes and before scanning, membranes were stored in TBS (BioPioneer Inc.). Densitometry of protein bands was performed in ImageStudio.

#### 4.3.6 High‐Molecular‐Weight Phospho‐Tau Bands Preparation for Mass Spectrometry (MS) Validation

Fresh ∼3 months‐of‐age CO lysates were prepared as above, homogenized in 1% NP‐40 buffer with protease and phosphatase inhibitors. Enrichment was done via incubating the lysate supernatant overnight with protein G Dynabeads (Invitrogen) pre‐loaded with fresh antibody, following manufacturer recommendations. After determining protein concentrations on the clear supernatant, 2 µg of protein were loaded for SDS‐PAGE in technical duplicates. Duplicate lanes were cut and stained with Coomassie blue. Using a scalpel, the bands were cut at the relative molecular weights where phospho‐Tau pT181 and pT217 had been previously observed (∼105 and ∼155 kDa). These samples were then in‐gel digested and prepared for MS analysis. Gel with remaining uncut samples was processed through normal WB for pT217 and pT181 to ensure pTau+ bands were successfully cut out.

#### 4.3.7 In‐Gel Digestion Sample Preparation

In‐gel digestion was performed following a previously published protocol with minor modifications. Briefly, gel bands corresponding to approximately 55, 105, and 150 kDa, as identified by western blot, were excised and cut into 1 mm^3^ cubes. Cysteine reduction and alkylation were performed by incubating the gel pieces in 5 mm Tris(2‐carboxyethyl)phosphine (TCEP) at room temperature for 30 min and 10 mm iodoacetamide in the dark for 30 min, respectively. The proteins were digested with MS‐grade trypsin/Lys‐C protease mix (Pierce) at 37°C for 18 h with shaking. Peptides were eluted by shaking in buffer containing 50% acetonitrile in 0.1% trifluoroacetic acid (TFA). Peptides were then desalted using C18 spin columns (Pierce) and dried using SpeedVac.

#### 4.3.8 Liquid Chromatography and Mass Spectrometry (MS) Analysis

Peptides were resuspended in 20 µL of 0.1% formic acid. Samples were analyzed using an Orbitrap Exploris 480 mass spectrometer coupled to a Vanquish Neo UHPLC system (Thermo Scientific). Peptides were first injected onto an PepMap Neo Trap Cartridge (300 µm X 5 mm, C18, 5 µm, 100 Å) and further separated on an EASY‐Spray PepMap Neo column (75 µm X 500 mm, C18, 2 µm, 100 Å) at a flow rate of 350 mL min^−1^ with an initial solvent composition of 98% buffer A (0.1% formic acid in water) and 2% buffer B (0.1% formic acid in 80% acetonitrile). Peptide eluted with a linear gradient with increasing buffer B from 2% to 20% over 82 min, followed by an increase from 20% to 35% buffer B for 20 min. The column was then washed with 99% buffer B for 8 min. The column was kept at 45°C at all times.

Mass spectrometry analysis was performed with Data‐independent acquisition (DIA) mode. Full MS1 scans were acquired with mass‐to‐charge ratios ranging from 380 to 980 m z^−1^ at a resolution of 1 20 000 and an automatic gain control (AGC) target of 3 × 10^6^. Precursor ions were isolated using 7 m z^−1^ window with 0.5 m z^−1^ overlap, generating 86 MS2 scans within the 380–980 m z^−1^ range. MS2 scans were performed at resolution of 30 000, a maximum ion injection time of 54 ms, AGC target of 2 × 10^6^, and a normalized collision energy of 28. MS1 data were acquired in profile mode, while MS2 data were acquired in centroid mode.

The DIA‐based mass spectrometry data were processed in Spectronaut 19 using library‐free (DirectDIA) analyses with the default search parameters.

#### 4.3.9 Native PAGE

Fresh CO lysates were homogenized in 1% NP‐40 buffer (0.5x Tris Buffer solution, 20 mm HEPES, 0.01% digitonin, 1% NP‐40) supplemented with protease and phosphatase inhibitor cocktails. To ensure a complete protein extraction, after homogenizing the COs, the samples were directly pulse ultrasonicated (∼24 s) before centrifuging at 20 600 *g* for 30 min. Supernatant was saved into a new tube, and protein was quantified and normalized using Pierce BCA assay (Thermo Fisher). Next, NATIVE‐PAGE was conducted –using NATIVE sample buffer (Invitrogen)– with NativePAGE 4%–16% Bis‐Tris gradient polyacrylamide gels (Thermo Fisher). Gel was then wet transferred onto PVDF membranes using a Mini Blot Module transfer system (Thermo Fisher) in NuPAGE transfer buffer (Life Technologies). The membrane was then washed with water and stained with Ponceau S stain as a protein loading and transfer control. Then, the membrane proceeded with washing with TBST, blocking with Li‐Cor TBS blocking buffer, and incubating overnight at 4°C with primary antibodies diluted in the blocking buffer with 0.02% TWEEN‐20 (antibody dilutions in Table 1). Membranes were then washed 3x with TBST before incubating with secondary antibodies (1:10 K) for 1–2 h at RT. After washing 3x with TBST, membranes were scanned in an Odyssey CLx scanner (LI‐COR). After washes and before scanning, membranes were stored in TBS (BioPioneer Inc.).

#### 4.3.10 Autophagy Flux Microscopy Assessment

DALGreen and DAPRed (Dojindo) were used following the manufacturer's protocol. In brief, DAPRed and DALGreen were diluted to working concentrations of 0.1 and 0.5 µm in Neural Media, respectively. Immediately before the start of a treatment with the compound, working solutions of DAPRed and DALGreen were incubated with COs for 90 min. Bafilomycin A1 (BafA1) was used as a control at a 300 nm concentration and was incubated with COs for a 24 h treatment. Similarly, autophagy modulating compounds (CCT, Rapa, Torin1) were incubated with COs and imaged 24 h following the start of the treatment. Live imaging was performed using a Zeiss Cell Discover 7 (CD7) after COs were transferred to clear, glass‐bottom 24 well plates. As temperature and CO2 levels were maintained in the CD7 during imaging, at least 3 z‐stacks per CO were captured in randomly selected fields per sample.

#### 4.3.11 Amyloid‐β ELISA

Fresh media (1 mL) per individual CO was conditioned over 24 h. Then, the levels of Aβ_1‐38_, Aβ_1‐40_, and Aβ_1‐42_ in the resulting conditioned media were measured using a V‐Plex Aβ Peptide Panel multiplex kit (Meso Scale Discovery) following manufacturer's instructions and recorded using an MESO QuickPlex Instrument (Meso Scale Discovery).

#### 4.3.12 CO Dissociation for Single Cell RNA‐Sequencing (scRNA‐Seq)

Individual COs of ∼7 weeks and 3 months of age were washed 4x in PBS and then dissociated with a Worthington Papain Dissociation kit (Worthington Biochemical Corp.) following a modified manufacturer's protocol. In brief, live organoids were placed in an Ultra‐Low Attachment 6‐well plate, manually minced in a Papain+DNase solution in Earle's medium, and incubated at 37°C in an orbital shaker at 100 rpm for 30 min. Then, the pieces were gently broken up with a 1 mL pipette before returning to shaking in an incubator for an additional 20–30 min. Afterwards, using a 10 mL pipette, the solution with the minced‐up pieces was gently mixed up and down 10 times and transferred to an empty 15 mL conical tube to allow undissociated debris to settle for ∼2 min. Subsequently, the cell suspension was transferred to a 3:8‐diluted ovomucoid protease inhibitor solution in Earle's medium, mixed, and then centrifuged at 300g for 7 min. The supernatant was then discarded, and the cells were gently resuspended in 1% BSA‐supplemented basal medium before filtering through a 40 µm mesh filter. Cells were then counted and confirmed to have viabilities above 85% using a Countess II Cell Counter (Thermo‐Fisher). Finally, samples were normalized to a concentration of ∼1000 cells µL^−1^ and transferred to 5 mL polypropylene tubes for processing at The Scripps Research Institute Genome Sequencing Core facility.

#### 4.3.13 scRNA‐Seq

Using the Chromium Next GEM single cell 3’ Cellplex v3.1 and Chip G kits (10x Genomics), the single cell partitioning, cDNA preparation, and sequencing libraries were prepared according to the manufacturer's protocol, using one 10x reaction for each experiment. After labeling the samples and amplifying the transcriptomic libraries, they were sequenced using on an Illumina Novaseq 6000 (SP or S1, depending on the run size). An average depth of ∼30 000 reads per cell was targeted. With a total number of 35 000–50 000 cells sequenced per run, adding up to 1.6 billion reads. See Table S1 for details, presented in Sheet 1 in the supplemental EXCEL file.

#### 4.3.14 scRNA‐Seq Data Preprocessing

The raw sequencer results were aligned to the GRCh38 genome assembly and de‐multiplexed using Cell Ranger (10x Genomics) with default parameters. The aligned gene expression matrices were then processed on R via Seurat (ver.4.3.0.1).

Initially, potentially dead cells and doublets were removed by removing any data points with mitochondrial gene load above 10% or with a number of detected genes under 500 or above 7500. The data was then log‐normalized, after which 2500 highly variable genes were identified, and the data was scaled using the default LogNormalize, vst, and negbinom methods; respectively. PCA and Uniform Manifold Approximation and Projection (UMAP) embedding were then performed with default parameters using the top 35 principal components (PC). For a thorough removal of doublets, remaining doublets were identified using the DoubletFinder package. The proportion of doublet formation was estimated from the number of inter‐sample doublets filtered by CellRanger for each independent experiment. After doublet removal, data were reprocessed with the same parameters. RNA counts, and the number of features across samples and unbiased clusters were assessed to ensure even sequencing quality and depth was maintained. Then, to correct for putative batch effects, dataset integration was performed [163] (Figure S2A). Specifically, the data sets from each independent experiment were integrated based on their similar top 2000 highly variable features (HVFs) cluster profiles using the FindIntegrationAnchors and IntegrateData Seurat functions using default parameters. Dimensional reduction, clustering, and UMAP embedding were finally recalculated with the top 30 PC using the integrated data. The resulting integrated data set ensured neither experimental batch nor genotype contributed to more than 10% of any of the final unbiased clustering's principal components.

#### 4.3.15 scRNA‐Seq Data Annotation, and Validation

Following recommended single‐cell annotation guidelines [164], cell type annotation was done via the aggregate use of multiple unbiased annotation tools along with the gene enrichment within each unsupervised cluster (UC) to inform and validate their cell type identity based on their relative predicted cell type transcriptomic signatures. These tools included, SuperCT [165, 166], suggesting single‐cell‐level annotation against reference cerebral organoid scRNA‐seq databases of Yoon et al. [110] and Camp et al. [13]; and the scType tool [65], providing unbiased cluster‐level annotation tool based on an expanded set of canonical markers of all human developmental brain and central nervous system‐related cell type markers assembled from the scType [65], PanglaoDB [66], and CellMarker [67, 68] databases (See Table S2, presented in Sheet 2 in the supplemental EXCEL file). All clusters with agreement between these unbiased annotation tools were further validated by confirming they exhibited high and specific expression of a curated set of canonical markers, while also demonstrating minimal or absent expression of markers associated with other cell types‐based on the aforementioned databases and the recently published CellxGene database (See Table S3, presented in Sheet 3 in the supplemental EXCEL file) [69]. Clusters with differing suggested identities were further sub‐clustered and checked for their unbiased enriched genes (via Seurat's FindMarkers function) until clear agreement and validation were achieved for each subset. Any subcluster with still conflicting or low‐confidence identities was annotated as unknown (Unk) under the “Other” general annotation. Finally, to facilitate data visualization, the negligibly small population of neuroepithelial cells (NEC) was merged into the transcriptionally similar choroid plexus‐annotated “ChorPlx” label; similarly, given their small populations, oligodendrocyte progenitor cells (OPC) and oligodendrocyte (Olig)‐annotated cells were merged into the “OPC/Olig” group. All automated and original annotations are available in the metadata. Visualization, cell type population, and gene expression plots were produced with standard Seurat package tools.

#### 4.3.16 Gene Set Enrichment Analysis and Comparisons

We focused on the most relevant cell types in the COs; namely, excitatory neurons, inhibitory neurons, and astrocytes. Starting with the master fully annotated data set, each cell type was extracted at a time, and Seurat's FindMarkers function was used to calculate the differentially expressed genes (DEGs) between each genotype and their isogenic control with a minimum detection rate of 15% of the cells in either group and with a log‐fold change cut‐off of 0.25.

To evaluate the transcriptional similarity between neurons derived from our cerebrocortical organoids and those reported in published studies [79], we performed a two‐step comparative analysis combining correlation‐based clustering and PCA. Initially, normalized gene expression matrices from both datasets were aligned by shared gene symbols. For each dataset, mean expression values were calculated for every neuronal subtype or cluster. Missing genes were assigned “zero” expression levels to maintain consistent feature dimensions. Pairwise Pearson correlation coefficients were computed to quantify transcriptomic similarity among the neuronal clusters, and the resulting correlation matrix was visualized as a hierarchical clustered heatmap. PCA was performed on the combined expression matrix to visualize global transcriptional relationships in reduced‐dimensional space.

#### 4.3.17 Pseudotime Analysis

Trajectory inference and pseudotime analysis were conducted using Monocle3 (v1.3.7) [77]. Briefly, raw count matrices and metadata were extracted from the Seurat object to create a cell_data_set (CDS). Batch effect correction was applied using the align_cds() function. A principal graph representing the developmental trajectory was constructed using learn_graph() function. To ensure biological relevance and avoid artificial branching, clusters disconnected from the main developmental trajectory were manually inspected and excluded. The trajectory graph was recalculated following cluster removal. Pseudotime ordering was performed using order_cells() function, with root cells assigned based on early progenitor populations or specific clusters informed by validated annotation. The expression dynamics of the key marker gene were visualized along pseudotime using plot_genes_in_pseudotime(), with additional faceting by cell types to reveal cell state‐specific gene expression patterns. To investigate genotype‐specific effects on trajectory, wild‐type cells were subset based on genotype annotations and processed independently following the same analytical pipeline.

#### 4.3.18 Microelectrode Array (MEA) Recordings of COs

COs of 6–8 weeks of age were attached to PEA/laminin–coated Axion Biosystems MEA plates and read on an Axion Biosystems Maestro Pro instrument. Specifically, 48‐well Cytoview MEA plates (M768‐tMEA‐48B) were coated with a 0.05% Poly(ethyleneimine) (Sigma‐Aldrich, P3143) in TC‐grade water solution overnight at 37°C. The wells were then thoroughly washed 3x with water, dried, and coated with a laminin I (R&D Systems, 3400‐010‐02) in PBS solution overnight at 37°C. Just before use, the laminin solution was aspirated, and COs were transferred one per well to the plate. COs were left to attach at the center of each well for about 15 min at 37°C before carefully filling the well with BrainPhys media (BrainPhys neuronal medium, 1x NeuroCult SM1, 1x N2 Max supplement). Two thirds of the media was changed every two days thereafter. The COs were allowed to adapt to this medium for at least 1 week before any data was recorded. To minimize metabolic and circadian rhythm activity variability, all CO electrophysiological activity data were collected for 5 min epochs ∼23–24 h after their last media change around the same time of day. CCT treatment period included the stable activity period 12–24 h after media change with drug and/or vehicle.

Analysis was performed using the Axion Biosystems software under the standard recommended settings for detection of action potentials on the Axion Navigator app and parameter analysis exported through the Neurometric Tool. Data from multiple timepoints were QC‐cleaned to exclude electrode recordings without a minimum resistance value of 18 Ω and/or without at least one voltage spike event per minute. Electrodes fulfilling these requisites were defined as “active electrodes”. A bursting event was defined by the default values of at least 5 spikes firing within a 100 ms time interval. To prevent bias caused by a varying number of active electrodes, weighted parameters were used, utilizing only the number of active electrodes. Specifically, we defined the Weighted Mean Firing Rate as the average spike frequency per active electrode. All timepoint recordings were aggregated using simple custom‐made *R* scripts (available upon request) to calculate each CO's average bursting rate and imported into GraphPad Prism for plotting.

#### 4.3.19 CO Treatments

To carefully control conditions, individual COs were placed into separate Ultra‐Low Attachment 24‐well plate wells with a pre‐defined media volume (e.g., 1.5 mL) across all conditions. DMSO vehicle was normalized to be equal across all conditions, independent of their compound concentration. COs were treated on every media change feed every two days in, shaking in orbital shakers at 100 rpm for experiments meant for immunoblotting or immunofluorescence analysis. Cellular viability was complementarily assessed with high‐sensitivity lactate dehydrogenase (LDH)‐Glo kits following manufacturers’ recommendations. For large‐scale experiments, samples were diluted 1:25 in LDH Storage buffer (200 mm Tris‐HCl, 10% Glycerol, 1% BSA, 7.3 pH) and kept at −80°C until ready to assay all timepoints together. For electrophysiology experiments in MEA, basal measurements were taken for at least 1 week before treatment. Then, at the time of treatment, the compounds were diluted in fresh BrainPhys media at concentrations accounting for the dilution due to partial media change. MEA viability was followed in real time throughout the whole experiment through the viability module (via the electrode resistances) collected at each activity recording. During the treatment period, more frequent recordings were performed during the previously established stable activity period of 12–28 h post‐media change. The Recovery period was defined as that after the compound washout through 5x consecutive fresh BrainPhys media replacements. To assess drug effects, mean activity values were calculated for each individual CO from the multiple recordings during 1–1.5 weeks before treatment (Basal), 12–28 h under drug exposure (Treatment), and 24–72 h after drug washout (still changing media to record only during the aforementioned stable activity periods).

#### 4.3.20 Image Analysis

Images were processed using ImageJ (Fiji v.2.9.0/1.53t). For CO size measurements, Cell Magic Wand tool was used to select each CO contour before measuring area. Regarding surface area coverage calculations, after which the area function measurement was used to quantify the percent surface area from individual images. Regarding puncta count, a mask identifying particles by size was used to filter and count distinct puncta from images. The measured signal from each image used for analysis was normalized to the total number of nuclei. Individual cells were identified by segmenting Hoechst‐stained images on the DAPI channel using a nuclei mask. This mask filtered nuclei by size and separated conjoined nuclei with the watershed function. Any artifacts or images with unreliable morphological staining, arising from technical issues or tissue anomalies such as folds or holes, were omitted from analyses.

#### 4.3.21 Quantification and Statistical Analysis

Data are reported as mean ± standard deviation. Each dot in the figures represents biological replicate samples of individual CO(s), unless otherwise stated in the figure legend. Experimental replicates were defined as biological replicates with samples deriving from both different CO induction dates and the date when the assay was performed. The number of samples per group (n) represents biological replicates and is indicated in legends. In Aβ ELISA, LDH‐Glo, and MEA recordings, duplicate to triplicate measurements of individual COs, considered technical replicates, were averaged to use their mean value as single biological sample data points.

All data comparisons and analyses were done in GraphPad Prism for Mac (version 9 through 10.0.3) using analysis of variance (ANOVA) analysis with the Prism‐recommended post‐hoc multiple comparisons test correction, as indicated in figure legends. Welch's ANOVA for multiple comparisons or t test for single comparisons was used where indicated in figure legends when data were statistically shown to be heteroscedastic.

Western blot (WB) data with experimental replicates were analyzed by 2‐way ANOVA with genotype/condition and experimental batch as the fixed factors and with Dunnett's multiple comparisons correction post‐hoc test. When possible, WB data were normalized by inter‐blot controls, as indicated in figure legends [167]. In cases where data were found not to be normally distributed, data were log‐transformed and re‐tested for normality; any such data are plotted in their log‐transformed form in the figures and indicated in the legends. Data with any overt outliers were tested via Grubbs’ test with a conservative alpha of 0.01. If confirmed by test, only up to a single outlier per data set was removed. When analyzing changes in CO functional activity due to treatments, pair‐wise comparisons across timepoints were assessed using a linear mixed‐effect model with restricted maximum likelihood (REML) estimation to account for repeated measures and within‐organoid correlations. Subjects (organoid replicates) were treated as random effects, enabling unbiased variance estimation and robust inference for changes between matched timepoints without sphericity assumptions. *p*‐value significance was illustrated according to: ^*^ *p *< 0.05, ^**^ *p* < 0.01, ^***^ *p* < 0.001, ^****^ *p* < 0.0001.

**TABLE 1:** Key resources table.

Reagent or Resource	Source	Identifier
Antibodies		
Anti‐alpha Tubulin (mouse, 1:1000)	Millipore	CP06
Anti‐SOX2 (rabbit, 1:250)	Cell Signaling Tech	3579
Anti‐MAP2 (chicken, 1:1000)	Invitrogen	PA1‐10005
Anti‐NeuN (mouse, 1:250)	Abcam	ab104224
Anti‐FOXG1 (rabbit, 1:250)	Abcam	ab18259
Anti‐CTIP2 (rat, 1:500)	Abcam	ab18465
Anti‐TBR1 (rabbit, 1:500)	Abcam	ab31940
Anti‐TUJ1/beta III‐tubulin (1:1000)	Abcam	ab41489
Anti‐SATB2 (mouse, 1:250)	Abcam	ab51502/ab92446
Anti‐S100 beta (mouse, 1:500)	Proteintech	66616‐1
Anti‐PLP1 (rabbit, 1:500)	Abcam	ab28486
Anti‐VGLUT1 (rabbit, 1:500)	SY‐SY	135–302
Anti‐VGLUT2 (rabbit, 1:500)	SY‐SY	135–403
Anti‐GABA (rabbit, 1:1000)	Invitrogen	PA5‐32241
Anti‐Tau (rabbit, 1:1000)	Santa Cruz biotech	sc‐166060
Anti‐PHF‐1 (mouse, 1:500)	Invitrogen	MN1020
Anti‐β‐Amyloid (D54D2) XP (rabbit, 1:150)	Cell Signaling Tech	8243
Anti‐pTau181 (mouse, 1:1000)	Invitrogen	MN1050
Anti‐pTau181 (rabbit, 1:1000)	Invitrogen	701530
Anti‐pTau217 (rabbit, 1:750)	Thermo	44‐744
Anti‐pTau217 (rabbit, 1:750)	Abcam	ab192665
Anti‐Synapsin I (rabbit, 1:100)	Cell Signaling Tech	5297
Anti‐LC3 (rabbit, 1:1000)	Cell Signaling Tech	2775S
LiCor Rabbit‐700	Li‐Cor	926–68021
LiCor Rabbit‐800	Li‐Cor	926–32210
Alexa Fluor 488 – anti‐Mouse IgG (1:2000)	Invitrogen	A32766
Alexa Fluor 555 – anti‐Mouse IgG (1:2000)	Invitrogen	A32773
Alexa Fluor 488 – anti‐Chicken IgG (1:2000)	Invitrogen	A32931
Alexa Fluor 555 – anti‐Chicken IgG (1:2000)	Invitrogen	A32932
Alexa Fluor 647 – anti‐Chicken IgG (1:2000)	Invitrogen	A32933
Alexa Fluor 488 – anti‐Rabbit IgG (1:2000)	Invitrogen	A32731
Alexa Fluor 647 – anti‐Rabbit IgG (1:2000)	Invitrogen	A32795
**Chemicals, peptides, and recombinant proteins**		
mTeSR1 Medium	StemCell Technologies	85852
Vitronectin (VTN‐N) Recombinant Human	Life Technologies	A14700
ReLeSR	Stem Cell Technologies	05873
Accutase	Innovative Cell Tec.	AT‐104
Chroman 1	MedChemExpress	HY‐15392
Emricasan	Selleckchem	S7775
Polyamine	Sigma Aldrich	P8483
Antioxidant supplement	Sigma Aldrich	A1345
Trans‐ISRIB	R&D Systems	5284
Essential 6 Medium	Life Technologies	A1516401
DMEM‐F12	Thermo	10565018
GlutaMAX	Thermo	35050061
Knockout serum replacement	Thermo	10828028
MEM Non‐essential amino acids	Gibco	11140‐050
β‐Mercaptoethanol	Thermo	21985023
Neurobasal‐A Medium	Life Technologies	10888‐022
B‐27 Supplement, minus vitamin A	Life Technologies	12587010
GlutaMAX Supplement	Life Technologies	35050‐061
Dorsomorphin	Sigma Aldrich	P5499‐5MG
SB‐431542	Tocris	1614
XAV‐939	Tocris	3748
EGF	R&D systems	236‐EG
FGF2	R&D systems	233‐FB
BDNF	Peprotech	450‐02
NT‐3	Peprotech	450‐03
Cultrex Mouse Laminin I	R&D systems	3400‐010‐02
Polyethylenimine	Sigma Aldrich	P3143
Brain Phys Neuronal medium	StemCell Technologies	057901
N‐2 max media supplement	R&D Systems	AR009
NeuroCult SM1 neuronal supplement	StemCell Technologies	05711
Hoechst 33342	Invitrogen	H3570
DAPRed—Autophagy Detection	Dojindo	D677
DALGreen—Autophagy Detection	Dojindo	D675
Dynabeads—Protein G	Invitrogen	3083363
Pierce RIPA Buffer	Thermo Scientific	89901
Halt Protease & Phosphatase Inhibitor Cocktail	Thermo Scientific	1861281
TBS 20X Solution	BioPioneer	MB1033
Tween 20	ThermoFisher	BP337‐500
Bolt Bis‐Tris 4%–12% WedgeWell 12‐well Gel	Invitrogen	NW04122BOX
Bolt Bis‐Tris 4%–12% WedgeWell 15‐well Gel	Invitrogen	NW04125BOX
TBST (10X)	Cell Signaling Technologies	9997S
Bolt Transfer Buffer (10X)	Invitrogen	BT00061
Bolt MES SDS Running Buffer (20X)	Invitrogen	B0002
IBlot3 Transfer Stacks Midi PVDF	Invitrogen	IB34001
**Critical commercial assays**		
Papain Dissociation System	Worthington Biochemical Corp.	LK003150
LDH‐Glo Cytotoxicity Assay	Promega	J2381
Chromium Next GEM Single Cell 3’ Kit v3.1	10x Genomics	1000269
Chromium Next GEM Chip G Single Cell Kit	10x Genomics	1000120
BCA Assay Kit	Pierce	23225
**Deposited data**		
scRNA‐seq	GEO	GSE301700
**Experimental models: Cell lines**		
7889B/7889O (WT/WT 1)	NYSCF Paquet, D., et al. (2016) [55].	CO0002‐01‐SV‐003 RRID:CVCL_B5IZ
7889B/7889O APP^Swe^/WT	NYSCF Paquet, D., et al. (2016) [55].	CO0002‐01‐CS‐003 (CVCL_B5IZ derivative mutant)
7889B/7889O PSEN1	NYSCF Paquet, D., et al. (2016) [55]	CO0002‐01‐CS‐002 (CVCL_B5IZ derivative mutant)
IID4 (WT/WT 2)	Gore A. et al. (2011) [168] Woodruff G. et al. (2013) [42].	CV‐hiPS‐B RRID:CVCL_1N86
ΔE9/WT	Woodruff G. et al. (2013) [42].	(CVCL_1N86 derivative mutant)
**Software and algorithms**		
Prism v10.0.3	GraphPad	GraphPad Prism. RRID:SCR_002798
Adobe Illustrator v29.2.1	Adobe	Adobe Illustrator. RRID:SCR_010279
ImageJ (Fiji v.2.9.0/1.53t)	Wayne Rasband, NIH, USA	Fiji. RRID:SCR_002285
CellRanger v7.1.0	10x Genomics	RRID:SCR_017344
sc‐type	Lanevski Aleksandr, FIMM, Finland	https://github.com/IanevskiAleksandr/sc‐type
Monocle3 (v1.3.7)	Monocle3	RRID:SCR_018685
**Other**		
AggreWell 800	StemCell Technologies	34815
Cell strainer, 40 µm	Corning	352340

## Author Contributions

Project conceptualization, supervision, and administration, S.R.L., J.W.K., and S.A.L. Cerebral organoid protocol optimization, S.R.L. and N.D. Cell and organoid culture, S.R.L., N.D., J.P.S., C.C.K., C.B., J.C., M.A., and A.B. Biochemistry, S.R.L., C.C.K., M.A., and A.P. Immunohistochemistry, S.R.L., A.P., J.P.S., C.C.K., and M.B. Microscopy, A.P., S.R.L., J.P.S., and C.C.K. Organoid electrophysiology and analysis, S.R.L., S.G., M.T., S.A.L. Single‐cell RNA‐seq design and preparation, S.R.L., D.T., T.S.M., S.R.H. scRNA‐seq analysis, S.R.L., Y.W., W.L., N.J.S., L.M. Mass spectrometry, Z.G. Writing of original draft, S.R.L. Manuscript proof‐reading and revisions, S.R.L., A.B., M.B., J.W.K, and S.A.L.

## Disclosure

J.W.K. discloses that he receives royalties for Tafamidis sales as an inventor and has received additional payments from Pfizer. J.W.K is a founder and major shareholder of Protego, which is developing immunoglobulin light chain kinetic stabilizers and other stabilizers for misfolding diseases; he serves on its Board of Directors and Scientific Advisory Board and acts as a consultant. He serves as a consultant for the Dominantly Inherited Alzheimer Network Trial Unit in reviewing drug candidates. S.A.L discloses that he is an inventor on worldwide patents for the use of memantine and NitroSynapsin (aka NitroMemantine, YQW‐036, or EM‐036) for neurodegenerative and neurodevelopmental disorders. Per Harvard University guidelines, S.A.L. participates in a royalty‐sharing agreement with his former institution Boston Children's Hospital/Harvard Medical School, which licensed the drug memantine (Namenda) to Forest Laboratories, Inc./Actavis/Allergan/AbbVie. S.A.L. was scientific founder of Adamas Pharmaceuticals, Inc. (now owned by Supernus Pharmaceuticals, Inc.), which developed or comarketed FDA‐approved forms of memantine‐ or amantadine‐containing drugs (NamendaXR, Namzaric, and GoCovri). NitroSynapsin is licensed to the biotechnology company EuMentis Therapeutics, Inc., for which SAL is scientific founder and chair of the Scientific Advisory Board (SAB). SAL is also a member of the SAB of Point 6 Bio. Ltd., and has recently served as a consultant to Circumvent Pharmaceuticals, Inc. Further, SAL discloses that he is a named inventor on patent(s) filed by his current institution, The Scripps Research Institute, for novel MEF2 and NRF2 transcriptional activators in the treatment of systemic and nervous system diseases via neuroprotective, anti‐inflammatory, and antioxidant actions

## Conflicts of Interest

J.W.K. receives royalties from Pfizer for Tafamidis, is a founder and shareholder of Protego, serves on its Board and SAB, consults for Protego and DIAN‐TU, and has received additional payments from Pfizer. S.A.L. is an inventor on patents for memantine and NitroSynapsin, receives royalties via Harvard/Boston Children's, is a founder of EuMentis and former founder of Adamas, serves on SABs of EuMentis and Point 6 Bio, and consults for biotech firms.

## Supporting information




**Supporting File 1**: advs73794‐sup‐0001‐SuppMat.docx.

**Supporting File 2**: advs73794‐sup‐0002‐Tables.xlsx.

## Data Availability

The data that support the findings of this study are openly available in GEO at https://doi.org/10.1101/2025.06.25.661453, reference number 301700.

## References

[1] R. Brookmeyer , N. Abdalla , C. H. Kawas , and M. M. Corrada , “Forecasting the Prevalence of Preclinical and Clinical Alzheimer's Disease in the United States,” Alzheimer's & Dementia 14, no. 2 (2018): 121–129, 10.1016/j.jalz.2017.10.009. PMC5803316 29233480

[2] B. Dubois , H. Hampel , H. H. Feldman , et al., “Preclinical Alzheimer's Disease: Definition, Natural History, and Diagnostic Criteria,” Alzheimer's & Dementia 12 (2016): 292–323, 10.1016/j.jalz.2016.02.002. PMC6417794 27012484

[3] C. R. Jack , D. S. Knopman , W. J. Jagust , et al., “Hypothetical Model of Dynamic Biomarkers of the Alzheimer's Pathological Cascade,” The Lancet Neurology 9, no. 1 (2010): 119–128, 10.1016/S1474-4422(09)70299-6. 20083042 PMC2819840

[4] C. Duyckaerts , M.‐C. Potier , and B. Delatour , “Alzheimer Disease Models and Human Neuropathology: Similarities and Differences,” Acta Neuropathologica 115, no. 1 (2008): 5–38, 10.1007/s00401-007-0312-8. 18038275 PMC2100431

[5] V. K. Ramanan and G. S. Day , “Anti‐amyloid Therapies for Alzheimer Disease: Finally, Good News for Patients,” Molecular Neurodegeneration 18, no. 1 (2023): 42, 10.1186/s13024-023-00637-0. 37381015 PMC10304213

[6] N. C. Cullen , P. Novak , D. Tosun , et al., “Efficacy Assessment of an Active Tau Immunotherapy in Alzheimer's Disease Patients with Amyloid and Tau Pathology: A Post Hoc Analysis of the “ADAMANT” Randomised, Placebo‐controlled, Double‐Blind, Multi‐Centre, Phase 2 Clinical Trial,” EBioMedicine 99 (2024): 104923. 38101301 10.1016/j.ebiom.2023.104923 PMC10733085

[7] B. P. Imbimbo , C. Balducci , S. Ippati , and M. Watling , “Initial Failures of Anti‐tau Antibodies in Alzheimer's Disease are Reminiscent of the Amyloid‐β Story,” Neural Regeneration Research 18, no. 1 (2023): 117, 10.4103/1673-5374.340409. 35799522 PMC9241406

[8] J. A. Miller , S. Horvath , and D. H. Geschwind , “Divergence of Human and Mouse Brain Transcriptome Highlights Alzheimer Disease Pathways,” Proceedings of the National Academy of Sciences 107, no. 28 (2010): 12698–12703, 10.1073/pnas.0914257107. PMC2906579 20616000

[9] T. C. Burns , M. D. Li , S. Mehta , A. J. Awad , and A. A. Morgan , “Mouse Models Rarely Mimic the Transcriptome of human Neurodegenerative Diseases: A Systematic Bioinformatics‐Based Critique of Preclinical Models,” European Journal of Pharmacology 759 (2015): 101–117, 10.1016/j.ejphar.2015.03.021. 25814260

[10] M. J. Kratochvil , A. J. Seymour , T. L. Li , S. P. Paşca , C. J. Kuo , and S. C. Heilshorn , “Engineered Materials for Organoid Systems,” Nature Reviews Materials 4, no. 9 (2019): 606–622, 10.1038/s41578-019-0129-9. PMC7864216 33552558

[11] S. A. Sloan , S. Darmanis , N. Huber , et al., “Human Astrocyte Maturation Captured in 3D Cerebral Cortical Spheroids Derived from Pluripotent Stem Cells,” Neuron 95, no. 4 (2017): 779–790.e6, 10.1016/j.neuron.2017.07.035. 28817799 PMC5890820

[12] M. A. Lancaster and J. A. Knoblich , “Generation of Cerebral Organoids From Human Pluripotent Stem Cells,” Nature Protocols 9, no. 10 (2014): 2329–2340, 10.1038/nprot.2014.158. 25188634 PMC4160653

[13] J. G. Camp , F. Badsha , M. Florio , et al., “Human Cerebral Organoids Recapitulate Gene Expression Programs of Fetal Neocortex Development,” Proceedings of the National Academy of Sciences 112, no. 51 (2015): 15672–15677, 10.1073/pnas.1520760112. PMC4697386 26644564

[14] C. Gonzalez , E. Armijo , J. Bravo‐Alegria , A. Becerra‐Calixto , C. E. Mays , and C. Soto , “Modeling Amyloid Beta and Tau Pathology in human Cerebral Organoids,” Molecular Psychiatry 23, no. 12 (2018): 2363–2374, 10.1038/s41380-018-0229-8. 30171212 PMC6594704

[15] L. Pellegrini and M. A. Lancaster , “Modeling Neurodegeneration with Mutant‐tau Organoids,” Cell 184, no. 17 (2021): 4377–4379, 10.1016/j.cell.2021.07.031. 34416145

[16] Y. H. Koh , L. Y. Tan , and S.‐Y. Ng , “Patient‐Derived Induced Pluripotent Stem Cells and Organoids for Modeling Alpha Synuclein Propagation in Parkinson's Disease,” Frontiers in Cellular Neuroscience 12 (2018): 413, 10.3389/fncel.2018.00413. 30483063 PMC6240766

[17] X. Qian , Y. Su , C. D. Adam , et al., “Sliced Human Cortical Organoids for Modeling Distinct Cortical Layer Formation,” Cell Stem Cell 26, no. 5 (2020): 766–781.e9, 10.1016/j.stem.2020.02.002. 32142682 PMC7366517

[18] H.‐K. Lee , C. Velazquez Sanchez , M. Chen , et al., “Three Dimensional Human Neuro‐Spheroid Model of Alzheimer's Disease Based on Differentiated Induced Pluripotent Stem Cells,” PLoS ONE 11, no. 9 (2016): 0163072, 10.1371/journal.pone.0163072. PMC5042502 27684569

[19] T. Vanova , J. Sedmik , J. Raska , et al., “Cerebral Organoids Derived from Patients with Alzheimer's Disease with PSEN1/2 Mutations Have Defective Tissue Patterning and Altered Development,” Cell Reports 42, no. 11 (2023): 113310. 37864790 10.1016/j.celrep.2023.113310

[20] M. Jorfi , C. D'Avanzo , R. E. Tanzi , D. Y. Kim , and D. Irimia , “Human Neurospheroid Arrays for in Vitro Studies of Alzheimer's Disease,” Scientific Reports 8, no. 1 (2018): 2450, 10.1038/s41598-018-20436-8. 29402979 PMC5799361

[21] H. Shimada , Y. Sato , T. Sasaki , et al., “A next‐generation iPSC‐derived Forebrain Organoid Model of Tauopathy with Tau Fibrils by AAV‐mediated Gene Transfer,” Cell Reports Methods 2, no. 9 (2022): 100289, 10.1016/j.crmeth.2022.100289. 36160042 PMC9499998

[22] Y.‐T. Lin , J. Seo , F. Gao , et al., “APOE4 Causes Widespread Molecular and Cellular Alterations Associated with Alzheimer's Disease Phenotypes in Human iPSC‐Derived Brain Cell Types,” Neuron 98, no. 6 (2018): 1141–1154.e7, 10.1016/j.neuron.2018.05.008. 29861287 PMC6023751

[23] Y. Gerakis and C. Hetz , “Brain Organoids: A Next Step for Humanized Alzheimer's Disease Models?,” Molecular Psychiatry 24, no. 4 (2019): 474–478, 10.1038/s41380-018-0343-7. 30617271

[24] J. Jo , L. Yang , H. D. Tran , et al., “Lewy Body–Like Inclusions in Human Midbrain Organoids Carrying Glucocerebrosidase and α‐Synuclein Mutations,” Annals of Neurology 90, no. 3 (2021): 490–505, 10.1002/ana.26166. 34288055 PMC9543721

[25] K. Grenier , J. Kao , and P. Diamandis , “Three‐Dimensional Modeling of human Neurodegeneration: Brain Organoids Coming of Age,” Molecular Psychiatry 25 (2019): 254–274. 31444473 10.1038/s41380-019-0500-7

[26] W. K. Raja , A. E. Mungenast , Y.‐T. Lin , et al., “Self‐Organizing 3D Human Neural Tissue Derived from Induced Pluripotent Stem Cells Recapitulate Alzheimer's Disease Phenotypes,” PLoS ONE 11, no. 9 (2016): 0161969, 10.1371/journal.pone.0161969. PMC5021368 27622770

[27] A. Bhaduri , M. G. Andrews , W. Mancia Leon , et al., “Cell Stress in Cortical Organoids Impairs Molecular Subtype Specification,” Nature 578, no. 7793 (2020): 142–148, 10.1038/s41586-020-1962-0. 31996853 PMC7433012

[28] P. S. Hou and H. C. Kuo , “Central Nervous System Organoids for Modeling Neurodegenerative Diseases,” Iubmb Life 74, no. 8 (2022): 812–825, 10.1002/iub.2595. 35102668

[29] I. Pereira , M. J. Lopez‐Martinez , and J. Samitier , “Advances in Current in Vitro Models on Neurodegenerative Diseases,” Frontiers in Bioengineering and Biotechnology 11 (2023): 1260397, 10.3389/fbioe.2023.1260397. 38026882 PMC10658011

[30] Y. Chang , J. Kim , H. Park , H. Choi , and J. Kim , “Modelling Neurodegenerative Diseases with 3D Brain Organoids,” Biological Reviews 95, no. 5 (2020): 1497–1509, 10.1111/brv.12626. 32568450

[31] N. Urrestizala‐Arenaza , S. Cerchio , F. Cavaliere , and C. Magliaro , “Limitations of human Brain Organoids to Study Neurodegenerative Diseases: A Manual to Survive,” Frontiers in Cellular Neuroscience 18 (2024): 1419526, 10.3389/fncel.2024.1419526. 39049825 PMC11267621

[32] J. Mertens , D. Reid , S. Lau , Y. Kim , and F. H. Gage , “Aging in a Dish: IPSC‐Derived and Directly Induced Neurons for Studying Brain Aging and Age‐Related Neurodegenerative Diseases,” Annual Review of Genetics 52 (2018): 271–293, 10.1146/annurev-genet-120417. PMC6415910 30208291

[33] J. Mertens , D. Reid , S. Lau , Y. Kim , and F. H. Gage , “Aging in a Dish: IPSC‐Derived and Directly Induced Neurons for Studying Brain Aging and Age‐Related Neurodegenerative Diseases,” Annual Review of Genetics 52 (2018): 271–293. 10.1146/annurev-genet-120417-031534 PMC6415910 30208291

[34] S. Schilling , A. Pradhan , A. Heesch , et al., “Differential Effects of Familial Alzheimer's Disease‐Causing Mutations on Amyloid Precursor Protein (APP) Trafficking, Proteolytic Conversion, and Synaptogenic Activity,” Acta Neuropathologica Communications 11, no. 1 (2023): 87, 10.1186/s40478-023-01577-y. 37259128 PMC10234039

[35] D. Kwart , A. Gregg , C. Scheckel , et al., “A Large Panel of Isogenic APP and PSEN1 Mutant Human iPSC Neurons Reveals Shared Endosomal Abnormalities Mediated by APP β‐CTFs, Not Aβ,” Neuron 104, no. 2 (2019): 256–270.e5, 10.1016/j.neuron.2019.07.010. 31416668

[36] K. Le Guennec , S. Veugelen , O. Quenez , et al., “Deletion of Exons 9 and 10 of the Presenilin 1 Gene in a Patient with Early‐onset Alzheimer Disease Generates Longer Amyloid Seeds,” Neurobiology of Disease 104 (2017): 97–103, 10.1016/j.nbd.2017.04.020. 28461250

[37] J. Liu , Q. Wang , D. Jing , et al., “Diagnostic Approach of Early‐Onset Dementia with Negative Family History: Implications from Two Cases of Early‐Onset Alzheimer's Disease with De Novo PSEN1 Mutation,” Journal of Alzheimer's Disease 68, no. 2 (2019): 551–558, 10.3233/JAD-181108. 30814350

[38] Y. Yang , E. Bagyinszky , and S. S. A. An , “Presenilin‐1 (PSEN1) Mutations: Clinical Phenotypes beyond Alzheimer's Disease,” International Journal of Molecular Sciences 24, no. 9 (2023): 8417. 37176125 10.3390/ijms24098417 PMC10179041

[39] M. Pagnon de la Vega , C. Näslund , R. Brundin , et al., “Mutation Analysis of Disease Causing Genes in Patients with Early Onset or Familial Forms of Alzheimer's Disease and Frontotemporal Dementia,” BMC Genomics 23, no. 1 (2022): 99, 10.1186/s12864-022-08343-9. 35120450 PMC8817590

[40] X. Gu , M. Zhao , X. Han , and L. Liu , “Presenilin‐1 Mutation Is Associated with a Hippocampus Defect in Alzheimer's Disease: Meta‐Analysis for Neuroimaging Research,” Clinical Neurology and Neurosurgery 191 (2020): 105679, 10.1016/j.clineuro.2020.105679. 32004985

[41] M. A. Riudavets , L. Bartoloni , J. C. Troncoso , et al., “Familial Dementia with Frontotemporal Features Associated with M146V Presenilin‐1 Mutation,” Brain Pathology 23, no. 5 (2013): 595–600, 10.1111/bpa.12051. 23489366 PMC4007155

[42] G. Woodruff , J. E. Young , F. J. Martinez , et al., “The Presenilin‐1 ΔE9 Mutation Results in Reduced γ‐Secretase Activity, but Not Total Loss of PS1 Function, in Isogenic Human Stem Cells,” Cell Reports 5, no. 4 (2013): 974–985, 10.1016/j.celrep.2013.10.018. 24239350 PMC3867011

[43] A. Aubert , M.‐G. Mendoza‐Ferri , A. Bramoulle , F. Studer , B. M. Colombo , and M. A. Mendoza‐Parra , “PSEN1^M146V^ and PSEN1^A246E^ Mutations Associated with Alzheimers Disease Impair Proper Microglia Differentiation,” BioRxiv (2023), 2023.10.08.561397.

[44] S. Ghatak , N. Dolatabadi , D. Trudler , et al., “Mechanisms of Hyperexcitability in Alzheimer's Disease hiPSC‐derived Neurons and Cerebral Organoids vs Isogenic Controls,” Elife 8 (2019): 50333, 10.7554/eLife.50333. PMC6905854 31782729

[45] S. Ghatak , N. Dolatabadi , R. Gao , et al., “NitroSynapsin Ameliorates Hypersynchronous Neural Network Activity in Alzheimer hiPSC Models,” Molecular Psychiatry 26, no. 10 (2021): 5751–5765, 10.1038/s41380-020-0776-7. 32467645 PMC7704704

[46] W. Zhang , C. Xu , J. Sun , H.‐M. Shen , J. Wang , and C. Yang , “Impairment of the Autophagy–lysosomal Pathway in Alzheimer's Diseases: Pathogenic Mechanisms and Therapeutic Potential,” Acta Pharmaceutica Sinica B 12, no. 3 (2022): 1019–1040, 10.1016/j.apsb.2022.01.008. 35530153 PMC9069408

[47] A. Piras , L. Collin , F. Grüninger , C. Graff , and A. Rönnbäck , “Autophagic and Lysosomal Defects in human Tauopathies: Analysis of Post‐Mortem Brain from Patients with Familial Alzheimer Disease, Corticobasal Degeneration and Progressive Supranuclear Palsy,” Acta Neuropathologica Communications 4 (2016): 22, 10.1186/s40478-016-0292-9. 26936765 PMC4774096

[48] A. Subramanian , T. Tamilanban , A. Alsayari , et al., “Trilateral Association of Autophagy, mTOR and Alzheimer's Disease: Potential Pathway in the Development for Alzheimer's Disease Therapy,” Frontiers in Pharmacology 13 (2022): 1094351, 10.3389/fphar.2022.1094351. 36618946 PMC9817151

[49] X.‐C. Zhu , J.‐T. Yu , T. Jiang , and L. Tan , “Autophagy Modulation for Alzheimer's Disease Therapy,” Molecular Neurobiology 48 (2013): 702–714, 10.1007/s12035-013-8457-z. 23625314

[50] L. Li , S. Zhang , X. Zhang , et al., “Autophagy Enhancer Carbamazepine Alleviates Memory Deficits and Cerebral Amyloid‐β Pathology in a Mouse Model of Alzheimer's Disease,” Current Alzheimer Research 10, no. 4 (2013): 433–441, 10.2174/1567205011310040008. 23305067

[51] J. Liu and L. Li , “Targeting Autophagy for the Treatment of Alzheimer's Disease: Challenges and Opportunities,” Frontiers in Molecular Neuroscience 12 (2019): 203, 10.3389/fnmol.2019.00203. 31507373 PMC6713911

[52] W. Jang , H. J. Kim , H. Li , K. D. Jo , M. K. Lee , and H. O. Yang , “The Neuroprotective Effect of Erythropoietin on Rotenone‐Induced Neurotoxicity in SH‐SY5Y Cells through the Induction of Autophagy,” Molecular Neurobiology 53, no. 6 (2016): 3812–3821, 10.1007/s12035-015-9316-x. 26156288

[53] C. Giorgi , G. Lombardozzi , F. Ammannito , et al., “Brain Organoids: A Game‐Changer for Drug Testing,” Pharmaceutics 16, no. 4 (2024): 443, 10.3390/pharmaceutics16040443. 38675104 PMC11054008

[54] U.S. Food & Drug Administration , FDA Announces Plan to Phase out Animal Testing Requirement for Monoclonal Antibodies and Other Drugs, (U.S. Food & Drug Administration, 2025).

[55] D. Paquet , D. Kwart , A. Chen , et al., “Efficient Introduction of Specific Homozygous and Heterozygous Mutations Using CRISPR/Cas9,” Nature 533, no. 7601 (2016): 125–129, 10.1038/nature17664. 27120160

[56] R. E. Tanzi , “The Genetics of Alzheimer Disease,” Cold Spring Harbor Perspectives in Medicine 2, no. 10 (2012): a006296–a006296, 10.1101/cshperspect.a006296. 23028126 PMC3475404

[57] M. Citron , T. Oltersdorf , C. Haass , et al., “Mutation of the β‐amyloid Precursor Protein in Familial Alzheimer's Disease Increases β‐protein Production,” Nature 360, no. 6405 (1992): 672–674, 10.1038/360672a0. 1465129

[58] J. Perez‐Tur , S. Froelich , G. Prihar , et al., “A Mutation in Alzheimerʼs Disease Destroying a Splice Acceptor Site in the Presenilin‐1 Gene,” Neuroreport 7, no. 1 (1995): 297–301, 10.1097/00001756-199512000-00071. 8742474

[59] J.‐H. Lee , G. Yoo , J. Choi , et al., “Cell‐line Dependency in Cerebral Organoid Induction: Cautionary Observations in Alzheimer's disease Patient‐derived Induced Pluripotent Stem Cells,” Molecular Brain 15, no. 1 (2022): 46, 10.1186/s13041-022-00928-5. 35578344 PMC9109296

[60] S. S. Kwak , K. J. Washicosky , E. Brand , et al., “Amyloid‐β42/40 Ratio Drives Tau Pathology in 3D human Neural Cell Culture Models of Alzheimer's Disease,” Nature Communications 11, no. 1 (2020): 1377, 10.1038/s41467-020-15120-3. PMC7070004 32170138

[61] M. A. Israel , S. H. Yuan , C. Bardy , et al., “Probing Sporadic and Familial Alzheimer's Disease Using Induced Pluripotent Stem Cells,” Nature 482, no. 7384 (2012): 216–220, 10.1038/nature10821. 22278060 PMC3338985

[62] S. A. Sloan , J. Andersen , A. M. Pașca , F. Birey , and S. P. Pașca , “Generation and Assembly of Human Brain Region–specific Three‐Dimensional Cultures,” Nature Protocols 13, no. 9 (2018): 2062–2085, 10.1038/s41596-018-0032-7. 30202107 PMC6597009

[63] M. A. Lancaster , N. S. Corsini , S. Wolfinger , et al., “Guided Self‐Organization and Cortical Plate Formation in Human Brain Organoids,” Nature Biotechnology 35, no. 7 (2017): 659–666, 10.1038/nbt.3906. PMC5824977 28562594

[64] S. P. Paşca , “Assembling Human Brain Organoids,” Science 363, no. 6423 (2019): 126. 30630918 10.1126/science.aau5729

[65] A. Ianevski , A. K. Giri , and T. Aittokallio , “Fully‐Automated and Ultra‐fast Cell‐type Identification Using Specific Marker Combinations from Single‐cell Transcriptomic Data,” Nature Communications 13, no. 1 (2022): 1246, 10.1038/s41467-022-28803-w. PMC8913782 35273156

[66] O. Franzén , L.‐M. Gan , and J. L. Björkegren , “PanglaoDB: A Web Server for Exploration of Mouse and human Single‐cell RNA Sequencing Data,” Database 2019 (2019): baz046. 30951143 10.1093/database/baz046 PMC6450036

[67] C. Hu , T. Li , Y. Xu , et al., “CellMarker 2.0: An Updated Database of Manually Curated Cell Markers in Human/Mouse and Web Tools Based on scRNA‐seq Data,” Nucleic Acids Research 51, no. D1 (2023): D870–D876. 36300619 10.1093/nar/gkac947 PMC9825416

[68] X. Zhang , Y. Lan , J. Xu , et al., “CellMarker: A Manually Curated Resource of Cell Markers in Human and Mouse,” Nucleic Acids Research 47, no. D1 (2019): D721–D728, 10.1093/nar/gky900. 30289549 PMC6323899

[69] CZI Cell Science Program , S. Abdulla , B. Aevermann , B. Aevermann , et al., “CZ CELL×GENE Discover: A Single‐cell Data Platform for Scalable Exploration, Analysis and Modeling of Aggregated Data,” Nucleic acids research 53, no. D1 (2025): D886–D900. 39607691 10.1093/nar/gkae1142 PMC11701654

[70] A. Jauhiainen , C. Thomsen , L. Strömbom , et al., “Distinct Cytoplasmic and Nuclear Functions of the Stress Induced Protein DDIT3/CHOP/GADD153,” PLoS ONE 7, no. 4 (2012): 33208, 10.1371/journal.pone.0033208. PMC3322118 22496745

[71] C. N. Byrns , A. E. Perlegos , K. N. Miller , et al., “Senescent Glia Link Mitochondrial Dysfunction and Lipid Accumulation,” Nature 630 (2024): 475–483. 38839958 10.1038/s41586-024-07516-8 PMC11168935

[72] T. Hai , C. D. Wolfgang , D. K. Marsee , A. E. Allen , and U. Sivaprasad , “ATF3 and Stress Responses,” Gene Expression 7, no. 4‐5‐6 (2018): 321. PMC6174666 10440233

[73] D. Saul , R. L. Kosinsky , E. J. Atkinson , et al., “A New Gene Set Identifies Senescent Cells and Predicts Senescence‐associated Pathways across Tissues,” Nature Communications 13, no. 1 (2022): 4827, 10.1038/s41467-022-32552-1. PMC9381717 35974106

[74] M. A. Sanborn , X. Wang , S. Gao , Y. Dai , and J. Rehman , “Unveiling the Cell‐type‐specific Landscape of Cellular Senescence through Single‐cell Transcriptomics Using SenePy,” Nature Communications 16, no. 1 (2025): 1884, 10.1038/s41467-025-57047-7. PMC11846890 39987255

[75] J. J. Palop and L. Mucke , “Network Abnormalities and Interneuron Dysfunction in Alzheimer Disease,” Nature Reviews Neuroscience 17, no. 12 (2016): 777–792, 10.1038/nrn.2016.141. 27829687 PMC8162106

[76] J. J. Palop , J. Chin , E. D. Roberson , et al., “Aberrant Excitatory Neuronal Activity and Compensatory Remodeling of Inhibitory Hippocampal Circuits in Mouse Models of Alzheimer's Disease,” Neuron 55, no. 5 (2007): 697–711, 10.1016/j.neuron.2007.07.025. 17785178 PMC8055171

[77] J. Cao , M. Spielmann , X. Qiu , et al., “The Single‐Cell Transcriptional Landscape of Mammalian Organogenesis,” Nature 566, no. 7745 (2019): 496–502, 10.1038/s41586-019-0969-x. 30787437 PMC6434952

[78] J. Cho , S. Bae , J. Jeon , et al., “Enhanced Differentiation of Neural Progenitor Cells in Alzheimer's Disease into Vulnerable Immature Neurons,” Iscience 28 (2025): 112446. 40384927 10.1016/j.isci.2025.112446 PMC12084003

[79] V. Gazestani , T. Kamath , N. M. Nadaf , et al., “Early Alzheimer's Disease Pathology in Human Cortex Involves Transient Cell States,” Cell 186, no. 20 (2023): 4438–4453, 10.1016/j.cell.2023.08.005. 37774681 PMC11107481

[80] J.‐P. Coppé , C. K. Patil , F. Rodier , et al., “Senescence‐Associated Secretory Phenotypes Reveal Cell‐Nonautonomous Functions of Oncogenic RAS and the p53 Tumor Suppressor,” PLoS Biology 6, no. 12 (2008): 301. 10.1371/journal.pbio.0060301 PMC2592359 19053174

[81] J. Li , F. Cao , H.‐L. Yin , et al., “Ferroptosis: Past, Present and Future,” Cell Death & Disease 11, no. 2 (2020): 88, 10.1038/s41419-020-2298-2. 32015325 PMC6997353

[82] N. Majerníková , A. Marmolejo‐Garza , C. S. Salinas , et al., “The Link between Amyloid β and Ferroptosis Pathway in Alzheimer's Disease Progression,” Cell Death & Disease 15, no. 10 (2024): 782, 10.1038/s41419-024-07152-0. 39468028 PMC11519607

[83] E. McDade , G. Wang , B. A. Gordon , et al., “Longitudinal Cognitive and Biomarker Changes in Dominantly Inherited Alzheimer Disease,” Neurology 91, no. 14 (2018): 1295, 10.1212/WNL.0000000000006277. PMC6177272 30217935

[84] S. Thordardottir , A. K. Ståhlbom , D. Ferreira , et al., “Preclinical Cerebrospinal Fluid and Volumetric Magnetic Resonance Imaging Biomarkers in Swedish Familial Alzheimer's Disease,” Journal of Alzheimer's disease 43, no. 4 (2014): 1393–1402, 10.3233/JAD-140339. 25182737

[85] C. Johansson , S. Thordardottir , J. Laffita‐Mesa , et al., “Gene‐variant Specific Effects of Plasma Amyloid‐β Levels in Swedish Autosomal Dominant Alzheimer Disease,” Alzheimer's Research & Therapy 16, no. 1 (2024): 207, 10.1186/s13195-024-01574-w. PMC11423518 39322953

[86] M. O. Quartey , J. N. Nyarko , J. M. Maley , et al., “The Aβ(1–38) Peptide Is a Negative Regulator of the Aβ1–42 Peptide Implicated in Alzheimer Disease Progression,” Scientific Reports 11, no. 1 (2021): 431, 10.1038/s41598-020-80164-w. 33432101 PMC7801637

[87] G. A. Braun , A. J. Dear , K. Sanagavarapu , H. Zetterberg , and S. Linse , “Amyloid‐β Peptide 37, 38 and 40 Individually and Cooperatively Inhibit Amyloid‐β 42 Aggregation,” Chemical Science 13, no. 8 (2022): 2423–2439, 10.1039/D1SC02990H. 35310497 PMC8864715

[88] M. E. Lame , E. E. Chambers , and M. Blatnik , “Quantitation of Amyloid Beta Peptides Aβ_1–38_, Aβ_1–40_, and Aβ_1–42_ in human Cerebrospinal Fluid by Ultra‐performance Liquid Chromatography–tandem Mass Spectrometry,” Analytical Biochemistry 419, no. 2 (2011): 133–139, 10.1016/j.ab.2011.08.010. 21888888

[89] E. Pretorius , M. J. Page , L. Hendricks , N. B. Nkosi , S. R. Benson , and D. B. Kell , “Both Lipopolysaccharide and Lipoteichoic Acids Potently Induce Anomalous Fibrin Amyloid Formation: Assessment with Novel Amytracker Stains,” Journal of The Royal Society Interface 15, no. 139 (2018): 20170941, 10.1098/rsif.2017.0941. 29445039 PMC5832738

[90] A. K. Torres , C. Jara , M. A. Olesen , and C. Tapia‐Rojas , “Pathologically Phosphorylated Tau at S396/404 (PHF‐1) Is Accumulated Inside of Hippocampal Synaptic Mitochondria of Aged Wild‐type Mice,” Scientific Reports 11, no. 1 (2021): 4448, 10.1038/s41598-021-83910-w. 33627790 PMC7904815

[91] H. Hampel , K. Blennow , L. M. Shaw , Y. C. Hoessler , H. Zetterberg , and J. Q. Trojanowski , “Total and Phosphorylated Tau Protein as Biological Markers of Alzheimer's Disease,” Experimental Gerontology 45 (2010): 30–40, 10.1016/j.exger.2009.10.010. 19853650 PMC2815003

[92] N. R. Barthélemy , R. J. Bateman , C. Hirtz , et al., “Cerebrospinal Fluid Phospho‐tau T217 Outperforms T181 as a Biomarker for the Differential Diagnosis of Alzheimer's Disease and PET Amyloid‐positive Patient Identification,” Alzheimer's Research & Therapy 12 (2020): 26. 10.1186/s13195-020-00596-4 PMC7079453 32183883

[93] N. R. Barthélemy , B. Saef , Y. Li , et al., “CSF Tau Phosphorylation Occupancies at T217 and T205 Represent Improved Biomarkers of Amyloid and Tau Pathology in Alzheimer's Disease,” Nature Aging 3, no. 4 (2023): 391–401, 10.1038/s43587-023-00380-7. 37117788 PMC10154225

[94] N. R. Barthélemy , R. J. Bateman , P. Marin , et al., “Tau Hyperphosphorylation on T217 in Cerebrospinal Fluid Is Specifically Associated to Amyloid‐β Pathology,” BioRxiv (2017): 226977.

[95] M. Kurihara , T. Matsubara , S. Morimoto , et al., “Neuropathological Changes Associated with Aberrant Cerebrospinal Fluid p‐tau181 and Aβ42 in Alzheimer's Disease and Other Neurodegenerative Diseases,” Acta Neuropathologica Communications 12, no. 1 (2024): 48, 10.1186/s40478-024-01758-3. 38539238 PMC10976730

[96] M. J. Ellis , C. Lekka , H. Tulmin , et al., “Validation of Tau Antibodies for Use in Western Blotting and Immunohistochemistry,” BioRxiv (2023), 536711, 2023.04.13.536711.

[97] M. J. Ellis , C. Lekka , K. L. Holden , et al., “Identification of High‐Performing Antibodies for the Reliable Detection of Tau Proteoforms by Western Blotting and Immunohistochemistry,” Acta Neuropathologica 147, no. 1 (2024): 87, 10.1007/s00401-024-02729-7. 38761203 PMC11102361

[98] Y. Zhou , J. Shi , D. Chu , et al., “Relevance of Phosphorylation and Truncation of Tau to the Etiopathogenesis of Alzheimer's Disease,” Frontiers in Aging Neuroscience 10 (2018): 27, 10.3389/fnagi.2018.00027. 29472853 PMC5810298

[99] S. Kaniyappan , R. R. Chandupatla , E.‐M. Mandelkow , and E. Mandelkow , “Extracellular Low‐n Oligomers of Tau Cause Selective Synaptotoxicity Without Affecting Cell Viability,” Alzheimer's & Dementia 13, no. 11 (2017): 1270–1291, 10.1016/j.jalz.2017.04.002. 28528849

[100] S. Lim , S. Shin , Y. Sung , et al., “Levosimendan Inhibits Disulfide Tau Oligomerization and Ameliorates Tau Pathology in Tau^P301L^‐BiFC Mice,” Experimental & Molecular Medicine 55, no. 3 (2023): 612–627, 10.1038/s12276-023-00959-5. 36914856 PMC10073126

[101] M. G. Spillantini and M. Goedert , “Tau Pathology and Neurodegeneration,” The Lancet Neurology 12, no. 6 (2013): 609–622, 10.1016/S1474-4422(13)70090-5. 23684085

[102] K. Kyrylkova , S. Kyryachenko , M. Leid , and C. Kioussi , “Detection of Apoptosis by TUNEL Assay,” Odontogenesis: Methods and protocols. (Springer, 2012): 41–47. 10.1007/978-1-61779-860-3_5 22566045

[103] S. Kari , K. Subramanian , I. A. Altomonte , A. Murugesan , O. Yli‐Harja , and M. Kandhavelu , “Programmed Cell Death Detection Methods: A Systematic Review and a Categorical Comparison,” Apoptosis 27, no. 7 (2022): 482–508, 10.1007/s10495-022-01735-y. 35713779 PMC9308588

[104] X.‐M. Hu , Z.‐X. Li , R.‐H. Lin , et al., “Guidelines for Regulated Cell Death Assays: A Systematic Summary, a Categorical Comparison, a Prospective,” Frontiers in Cell and Developmental Biology 9 (2021): 634690, 10.3389/fcell.2021.634690. 33748119 PMC7970050

[105] I. Espuny‐Camacho , A. M. Arranz , M. Fiers , et al., “Hallmarks of Alzheimer's Disease in Stem‐Cell‐Derived Human Neurons Transplanted into Mouse Brain,” Neuron 93, no. 5 (2017): 1066–1081.e8, 10.1016/j.neuron.2017.02.001. 28238547

[106] H. Tanabe , S. Maeda , E. Sano , N. Sakai , S. Endoh‐Yamagami , and H. Okano , “Tau Aggregation Induces Cell Death in iPSC‐Derived Neurons,” Aging Brain 7 (2025): 100136, 10.1016/j.nbas.2025.100136. 40276591 PMC12018045

[107] E. I. Parrotta , V. Lucchino , C. Zannino , et al., “Modeling Sporadic Progressive Supranuclear Palsy in 3D Midbrain Organoids: Recapitulating Disease Features for In Vitro Diagnosis and Drug Discovery,” Annals of Neurology 97, no. 5 (2025): 845–859, 10.1002/ana.27172. 39876539 PMC12010066

[108] H. T. Sakurai , H. Iwashita , S. Arakawa , et al., “Development of Small Fluorescent Probes for the Analysis of Autophagy Kinetics,” Iscience 26, no. 7 (2023): 107218. 37456828 10.1016/j.isci.2023.107218 PMC10339198

[109] L. Yoon , R. C. Botham , A. Verhelle , et al., “An mTOR‐independent Macroautophagy Activator Ameliorates Tauopathy and Prionopathy Neurodegeneration Phenotypes,” BioRxiv (2022), 509997, 2022.09.29.509997.

[110] S.‐J. Yoon , L. S. Elahi , A. M. Pașca , et al., “Reliability of Human Cortical Organoid Generation,” Nature Methods 16, no. 1 (2019): 75–78, 10.1038/s41592-018-0255-0. 30573846 PMC6677388

[111] A. B. Pupyshev , V. M. Belichenko , M. V. Tenditnik , et al., “Combined Induction of mTOR‐dependent and mTOR‐independent Pathways of Autophagy Activation as an Experimental Therapy for Alzheimer's Disease‐Like Pathology in a Mouse Model,” Pharmacology Biochemistry and Behavior 217 (2022): 173406, 10.1016/j.pbb.2022.173406. 35609863

[112] S. R. Stockwell , G. Platt , S. E. Barrie , et al., “Mechanism‐Based Screen for G1/S Checkpoint Activators Identifies a Selective Activator of EIF2AK3/PERK Signalling,” PLoS ONE 7, no. 1 (2012): 28568, 10.1371/journal.pone.0028568. PMC3257223 22253692

[113] X. Li , X. Yu , D. Zhou , et al., “CCT020312 Inhibits Triple‐Negative Breast Cancer through PERK Pathway‐Mediated G1 Phase Cell Cycle Arrest and Apoptosis,” Frontiers in Pharmacology 11 (2020): 737, 10.3389/fphar.2020.00737. 32508655 PMC7250150

[114] Y. Lei , L. He , C. Yan , Y. Wang , and G. Lv , “PERK Activation by CCT020312 Chemosensitizes Colorectal Cancer through Inducing Apoptosis Regulated by ER Stress,” Biochemical and biophysical research communications 557 (2021): 316–322, 10.1016/j.bbrc.2021.03.041. 33894420

[115] B.‐M. Tang , Z.‐W. Li , and Z.‐Y. Wang , “PERK Activator CCT020312 Prevents Inflammation‐mediated Osteoporosis in the Ovariectomized Rats,” Gynecological Endocrinology 37, no. 4 (2021): 342–348, 10.1080/09513590.2021.1874904. 33480297

[116] D. Zhou , M. Yin , B. Kang , et al., “CCT020312 exerts Anti‐prostate Cancer Effect by Inducing G1 Cell Cycle Arrest, Apoptosis and Autophagy through Activation of PERK/eIF2α/ATF4/CHOP Signaling,” Biochemical Pharmacology 221 (2024): 116038, 10.1016/j.bcp.2024.116038. 38286211

[117] X. Li , D. Lu , L. Zou , et al., “Activation of the PERK Branch of the UPR As a Strategy for Improving Outcomes in Acute Ischemic Stroke,” ACS Omega 10, no. 15 (2025): 15461–15470, 10.1021/acsomega.5c00125. 40290914 PMC12019423

[118] M. E. Fusakio , J. A. Willy , Y. Wang , et al., “Transcription Factor ATF4 Directs Basal and Stress‐induced Gene Expression in the Unfolded Protein Response and Cholesterol Metabolism in the Liver,” Molecular Biology of the Cell 27, no. 9 (2016): 1536–1551, 10.1091/mbc.E16-01-0039. 26960794 PMC4850040

[119] D. Wu and J. Liang , “Activating Transcription Factor 4: A Regulator of Stress Response in human Cancers,” Frontiers in Cell and Developmental Biology 12 (2024): 1370012, 10.3389/fcell.2024.1370012. 38601083 PMC11004295

[120] P. Lindner , S. B. Christensen , P. Nissen , J. V. Møller , and N. Engedal , “Cell Death Induced by the ER Stressor Thapsigargin Involves Death Receptor 5, A Non‐autophagic Function of MAP1LC3B, and Distinct Contributions from Unfolded Protein Response Components,” Cell Communication and Signaling 18 (2020): 12, 10.1186/s12964-019-0499-z. 31987044 PMC6986015

[121] L. H. Chen , C. C. Jiang , K. A. Kiejda , et al., “Thapsigargin Sensitizes Human Melanoma Cells to TRAIL‐induced Apoptosis by Up‐Regulation of TRAIL‐R2 through the Unfolded Protein Response,” Carcinogenesis 28, no. 11 (2007): 2328–2336, 10.1093/carcin/bgm173. 17652336

[122] C. Sidrauski , A. M. McGeachy , N. T. Ingolia , and P. Walter , “The Small Molecule ISRIB Reverses the Effects of eIF2α Phosphorylation on Translation and Stress Granule Assembly,” Elife 4 (2015): 05033. 10.7554/eLife.05033 PMC4341466 25719440

[123] S. Ghatak , T. Nakamura , and S. A. Lipton , “Aberrant Protein S‐nitrosylation Contributes to Hyperexcitability‐Induced Synaptic Damage in Alzheimer's Disease: Mechanistic Insights and Potential Therapies,” Frontiers in Neural Circuits 17 (2023): 1099467, 10.3389/fncir.2023.1099467. 36817649 PMC9932935

[124] D. Putcha , M. Brickhouse , K. O'Keefe , et al., “Hippocampal Hyperactivation Associated With Cortical Thinning in Alzheimer's Disease Signature Regions in Non‐Demented Elderly Adults,” The Journal of Neuroscience 31, no. 48 (2011): 17680–17688, 10.1523/JNEUROSCI.4740-11.2011. 22131428 PMC3289551

[125] I. Nishida , K. Yamada , A. Sakamoto , T. Wakabayashi , and T. Iwatsubo , “Chronic Neuronal Hyperexcitation Exacerbates Tau Propagation in a Mouse Model of Tauopathy,” International Journal of Molecular Sciences 25, no. 16 (2024): 9004, 10.3390/ijms25169004. 39201689 PMC11354494

[126] S. A. Toniolo , A. Sen , and M. Husain , “Modulation of Brain Hyperexcitability: Potential New Therapeutic Approaches in Alzheimer's Disease,” International Journal of Molecular Sciences 21, no. 23 (2020): 9318. 33297460 10.3390/ijms21239318 PMC7730926

[127] Y. T. Quiroz , A. E. Budson , K. Celone , et al., “Hippocampal Hyperactivation in Presymptomatic Familial Alzheimer's Disease,” Annals of Neurology 68, no. 6 (2010): 865–875, 10.1002/ana.22105. 21194156 PMC3175143

[128] C. Holmes , “Genotype and Phenotype in Alzheimer's Disease,” British Journal of Psychiatry 180, no. 2 (2002): 131–134, 10.1192/bjp.180.2.131. 11823322

[129] T. Raj , Y. I. Li , G. Wong , et al., “Integrative Transcriptome Analyses of the Aging Brain Implicate Altered Splicing in Alzheimer's Disease Susceptibility,” Nature Genetics 50, no. 11 (2018): 1584–1592, 10.1038/s41588-018-0238-1. 30297968 PMC6354244

[130] P. Spitzer , L.‐M. Mulzer , T. J. Oberstein , et al., “Microvesicles from Cerebrospinal Fluid of Patients with Alzheimer's Disease Display Reduced Concentrations of Tau and APP Protein,” Scientific Reports 9, no. 1 (2019): 7089, 10.1038/s41598-019-43607-7. 31068645 PMC6506501

[131] C. Reitz , “Alzheimer′s Disease and the Amyloid Cascade Hypothesis: A Critical Review,” International Journal of Alzheimer's Disease 2012, no. 1 (2012): 369808. 10.1155/2012/369808 PMC3313573 22506132

[132] C. Arber , J. Toombs , C. Lovejoy , et al., “Familial Alzheimer's Disease Patient‐Derived Neurons Reveal Distinct Mutation‐specific Effects on Amyloid Beta,” Molecular Psychiatry 25, no. 11 (2019): 2919–2931. 30980041 10.1038/s41380-019-0410-8 PMC7577860

[133] A. Singh , D. Allen , A. Fracassi , et al., “Functional Integrity of Synapses in the Central Nervous System of Cognitively Intact Individuals with High Alzheimer's Disease Neuropathology Is Associated with Absence of Synaptic Tau Oligomers,” Journal of Alzheimer's Disease 78, no. 4 (2020): 1661–1678, 10.3233/JAD-200716. PMC7836055 33185603

[134] S. Holper , R. Watson , and N. Yassi , “Tau as a Biomarker of Neurodegeneration,” International Journal of Molecular Sciences 23, no. 13 (2022): 7307, 10.3390/ijms23137307. 35806324 PMC9266883

[135] V. H. Man , X. He , F. Han , et al., “Phosphorylation at Ser289 Enhances the Oligomerization of Tau Repeat R2,” Journal of Chemical Information and Modeling 63, no. 4 (2023): 1351–1361, 10.1021/acs.jcim.2c01597. 36786552 PMC10032562

[136] E. Ercan‐Herbst , J. Ehrig , D. C. Schöndorf , et al., “A Post‐Translational Modification Signature Defines Changes in Soluble Tau Correlating with Oligomerization in Early Stage Alzheimer's disease Brain,” Acta Neuropathologica Communications 7 (2019): 192, 10.1186/s40478-019-0823-2. 31796124 PMC6892178

[137] G. Niewiadomska , W. Niewiadomski , M. Steczkowska , and A. Gasiorowska , “Tau Oligomers Neurotoxicity,” Life 11, no. 1 (2021): 28, 10.3390/life11010028. 33418848 PMC7824853

[138] A. Verheyen , A. Diels , J. Dijkmans , et al., “Using Human iPSC‐Derived Neurons to Model TAU Aggregation,” PLoS ONE 10, no. 12 (2015): 0146127, 10.1371/journal.pone.0146127. PMC4697850 26720731

[139] L. Miguel , A. Rovelet‐Lecrux , M. Feyeux , et al., “Detection of all Adult Tau Isoforms in a 3D Culture Model of iPSC‐derived Neurons,” Stem Cell Research 40 (2019): 101541, 10.1016/j.scr.2019.101541. 31522011

[140] K. M. Schoch , S. L. DeVos , R. L. Miller , et al., “Increased 4R‐Tau Induces Pathological Changes in a Human‐Tau Mouse Model,” Neuron 90, no. 5 (2016): 941–947, 10.1016/j.neuron.2016.04.042. 27210553 PMC5040069

[141] M. Ebashi , S. Toru , A. Nakamura , et al., “Detection of AD‐specific Four Repeat Tau with Deamidated Asparagine Residue 279‐specific Fraction Purified from 4R Tau Polyclonal Antibody,” Acta Neuropathologica 138, no. 1 (2019): 163–166, 10.1007/s00401-019-02012-0. 31006065 PMC6570692

[142] D. Panda , J. C. Samuel , M. Massie , S. C. Feinstein , and L. Wilson , “Differential Regulation of Microtubule Dynamics by Three‐ and Four‐repeat Tau: Implications for the Onset of Neurodegenerative Disease,” Proceedings of the National Academy of Sciences 100, no. 16 (2003): 9548–9553, 10.1073/pnas.1633508100. PMC170955 12886013

[143] S. S. Harris , R. Ellingford , J. Hartmann , et al., “Alzheimer's disease Patient‐Derived High‐Molecular‐Weight Tau Impairs Bursting in Hippocampal Neurons,” Cell 188, no. 14 (2025): 3775–3788. 40300603 10.1016/j.cell.2025.04.006 PMC12255526

[144] A. M. Eteleeb , B. C. Novotny , C. S. Tarraga , et al., “Brain High‐throughput Multi‐Omics Data Reveal Molecular Heterogeneity in Alzheimer's Disease,” PLoS Biology 22, no. 4 (2024): 3002607, 10.1371/journal.pbio.3002607. PMC11086901 38687811

[145] A. Sziraki , Z. Lu , J. Lee , et al., “A Global View of Aging and Alzheimer's Pathogenesis‐Associated Cell Population Dynamics and Molecular Signatures in human and Mouse Brains,” Nature Genetics 55, no. 12 (2023): 2104–2116, 10.1038/s41588-023-01572-y. 38036784 PMC10703679

[146] W. Luo , W. Qu , and L. Gan , “The AD Odyssey 2023: Tales of Single Cell,” Cell 186, no. 20 (2023): 4257–4259, 10.1016/j.cell.2023.09.001. 37774675

[147] M. G. Andrews and A. R. Kriegstein , “Challenges of Organoid Research,” Annual Review of Neuroscience 45, no. 1 (2022): 23–39, 10.1146/annurev-neuro-111020-090812. PMC10559943 34985918

[148] H. Y. Chung , D. H. Kim , E. K. Lee , et al., “Redefining Chronic Inflammation in Aging and Age‐Related Diseases: Proposal of the Senoinflammation Concept,” Aging and Disease 10, no. 2 (2019): 367, 10.14336/AD.2018.0324. 31011483 PMC6457053

[149] E. Sikora , A. Bielak‐Zmijewska , M. Dudkowska , et al., “Cellular Senescence in Brain Aging,” Frontiers in Aging Neuroscience 13 (2021): 646924, 10.3389/fnagi.2021.646924. 33732142 PMC7959760

[150] A. Lasry and Y. Ben‐Neriah , “Senescence‐Associated Inflammatory Responses: Aging and Cancer Perspectives,” Trends in Immunology 36, no. 4 (2015): 217–228, 10.1016/j.it.2015.02.009. 25801910

[151] X. Zhang , X. Chen , Q. Liu , S. Zhang , and W. Hu , “Translation Repression via Modulation of the Cytoplasmic Poly(A)‐binding Protein in the Inflammatory Response,” Elife 6 (2017): 27786. 10.7554/eLife.27786 PMC5507668 28635594

[152] A. S. Das , A. Basu , and R. Mukhopadhyay , “Ribosomal Proteins: the Missing Piece in the Inflammation Puzzle?,” Molecular and Cellular Biochemistry 480, no. 2 (2025): 785–797, 10.1007/s11010-024-05050-9. 38951378

[153] N. Mattsson , J. M. Schott , J. Hardy , M. R. Turner , and H. Zetterberg , “Selective Vulnerability in Neurodegeneration: Insights from Clinical Variants of Alzheimer's Disease,” Journal of Neurology, Neurosurgery & Psychiatry 87, no. 9 (2016): 1000–1004, 10.1136/jnnp-2015-311321. 26746185

[154] K. Leng , E. Li , R. Eser , et al., “Molecular Characterization of Selectively Vulnerable Neurons in Alzheimer's Disease,” Nature Neuroscience 24, no. 2 (2021): 276–287, 10.1038/s41593-020-00764-7. 33432193 PMC7854528

[155] Y. Li , C. P. Serras , J. Blumenfeld , et al., “Cell‐Type‐Directed Network‐Correcting Combination Therapy for Alzheimer's Disease,” Cell 188, no. 20 (2025): 5516–5534. 40695276 10.1016/j.cell.2025.06.035 PMC12313259

[156] A. Papanikolaou , D. Graykowski , B. I. Lee , et al., “Selectively Vulnerable Deep Cortical Layer 5/6 Fast‐spiking Interneurons in Alzheimer's disease Models In Vivo,” Neuron 113 (2025): 2265–2279. 40345184 10.1016/j.neuron.2025.04.010

[157] S.‐M. Chou , Y.‐H. Yen , F. Yuan , S.‐C. Zhang , and C.‐M. Chong , “Neuronal Senescence in the Aged Brain,” Aging and Disease 14, no. 5 (2023): 1618, 10.14336/AD.2023.0214. 37196117 PMC10529744

[158] M. T. Corasaniti , G. Bagetta , P. Nicotera , et al., “Exploitation of Autophagy Inducers in the Management of Dementia: A Systematic Review,” International Journal of Molecular Sciences 25, no. 2 (2024): 1264, 10.3390/ijms25021264. 38279266 PMC10816917

[159] S. Kshirsagar , A. P. Reddy , and P. H. Reddy , “Beneficial Effects of Mitophagy Enhancers on Amyloid Beta‐induced Mitochondrial and Synaptic Toxicities in Alzheimer's Disease,” Mitochondrion 83 (2025): 102038, 10.1016/j.mito.2025.102038. 40157622

[160] C. A. Tristan , H. Hong , Y. Jethmalani , et al., “Efficient and Safe Single‐Cell Cloning of Human Pluripotent Stem Cells Using the CEPT Cocktail,” Nature Protocols 18, no. 1 (2023): 58–80, 10.1038/s41596-022-00753-z. 36261632 PMC11009857

[161] S. Ryu , C. Weber , P.‐H. Chu , et al., “Stress‐Free Cell Aggregation by Using the CEPT Cocktail Enhances Embryoid Body and Organoid Fitness,” Biofabrication 16, no. 1 (2023): 015016, 10.1088/1758-5090/ad0d13. 37972398

[162] Y. Chen , C. A. Tristan , L. Chen , et al., “A Versatile Polypharmacology Platform Promotes Cytoprotection and Viability of human Pluripotent and Differentiated Cells,” Nature Methods 18, no. 5 (2021): 528–541, 10.1038/s41592-021-01126-2. 33941937 PMC8314867

[163] S. Durinck , P. T. Spellman , E. Birney , and W. Huber , “Mapping Identifiers for the Integration of Genomic Datasets with the R/Bioconductor Package biomaRt,” Nature Protocols 4 (2009): 1184–1191, 10.1038/nprot.2009.97. 19617889 PMC3159387

[164] Z. A. Clarke , T. S. Andrews , J. Atif , et al., “Tutorial: Guidelines for Annotating Single‐cell Transcriptomic Maps Using Automated and Manual Methods,” Nature Protocols 16, no. 6 (2021): 2749–2764, 10.1038/s41596-021-00534-0. 34031612

[165] P. Xie , M. Gao , C. Wang , et al., “SuperCT: A Supervised‐Learning Framework for Enhanced Characterization of Single‐cell Transcriptomic Profiles,” Nucleic Acids Research 47, no. 8 (2019): 48, 10.1093/nar/gkz116. PMC6486558 30799483

[166] J. Zhong and W. Lin , “Use of SuperCT for Enhanced Characterization of Single‐Cell Transcriptomic Profiles,” Stem Cell Transcriptional Networks: Methods and Protocols, (Springer, 2020): 169–177. 10.1007/978-1-0716-0301-7_9 31960378

[167] S. C. Taylor , L. K. Rosselli‐Murai , B. Crobeddu , and I. Plante , “A Critical Path to Producing High Quality, Reproducible Data from Quantitative Western Blot Experiments,” Scientific Reports 12, no. 1 (2022): 17599, 10.1038/s41598-022-22294-x. 36266411 PMC9585080

[168] A. Gore , Z. Li , H.‐L. Fung , et al., “Somatic Coding Mutations in Human Induced Pluripotent Stem Cells,” Nature 471, no. 7336 (2011): 63–67, 10.1038/nature09805. 21368825 PMC3074107

