# Supplementary material for: Autophagy Activators Normalize Aberrant Tau Proteostasis and Rescue Synapses in Human Familial Alzheimer's Disease iPSC‐Derived Cortical Organoids
Source: Adv Sci (Weinh). 2026 Jan 27;13(17):e14783. doi: 10.1002/advs.202514783 (PMC13042409; doi:10.1002/advs.202514783)
Supplement: Supplementary file 1 — Supporting File 1: advs73794‐sup‐0001‐SuppMat.docx. [file ADVS-13-e14783-s002.docx]

Supplementary Figures

**Autophagy Activators Normalize Aberrant Tau Proteostasis and Rescue Synapses in Human Familial Alzheimer’s Disease iPSC-Derived Cortical Organoids**

Sergio R. Labra^1,2,3^, Jadon Compher^1,2^, Akhil Prabhavalkar^1,2,3^, Mireya Almaraz^1,2,3^, Claudia Cedeño Kwong^1,3^, Christine Baal^1,2^, Maria Talantova^1,2^, Nima Dolatabadi^1,2^, Julian Piña-Sanz^1,2,3^, Yubo Wang^1,2^, Leonard Yoon^1,3^, Swagata Ghatak^1,2,6^, Zi Gao^1,3^, Yuting Zhang^1,3^, Dorit Trudler^1,2^, Lynee Massey^1,3^, Wei Lin^4^, Anthony Balistreri^1,3^, Michael Bula^1,2^, Nicholas J. Schork^4^, Tony S. Mondala^5^, Steven R. Head^5^, Jeffery W. Kelly^1,3,*^, Stuart A. Lipton^1,2,*^

^1^Neurodegeneration New Medicines Center, The Scripps Research Institute, La Jolla, CA, USA; ^2^Department of Molecular and Cellular Biology, The Scripps Research Institute, La Jolla, CA, USA; ^3^Department of Chemistry, The Scripps Research Institute, La Jolla, CA, USA;

^4^Translational Genomics Research Institute, Phoenix, AZ, USA; ^5^Genomics Core, The Scripps Research Institute, La Jolla, CA, USA; ^6^School of Biological Sciences, National Institute of Science Education and Research, Bhubaneswar, India.


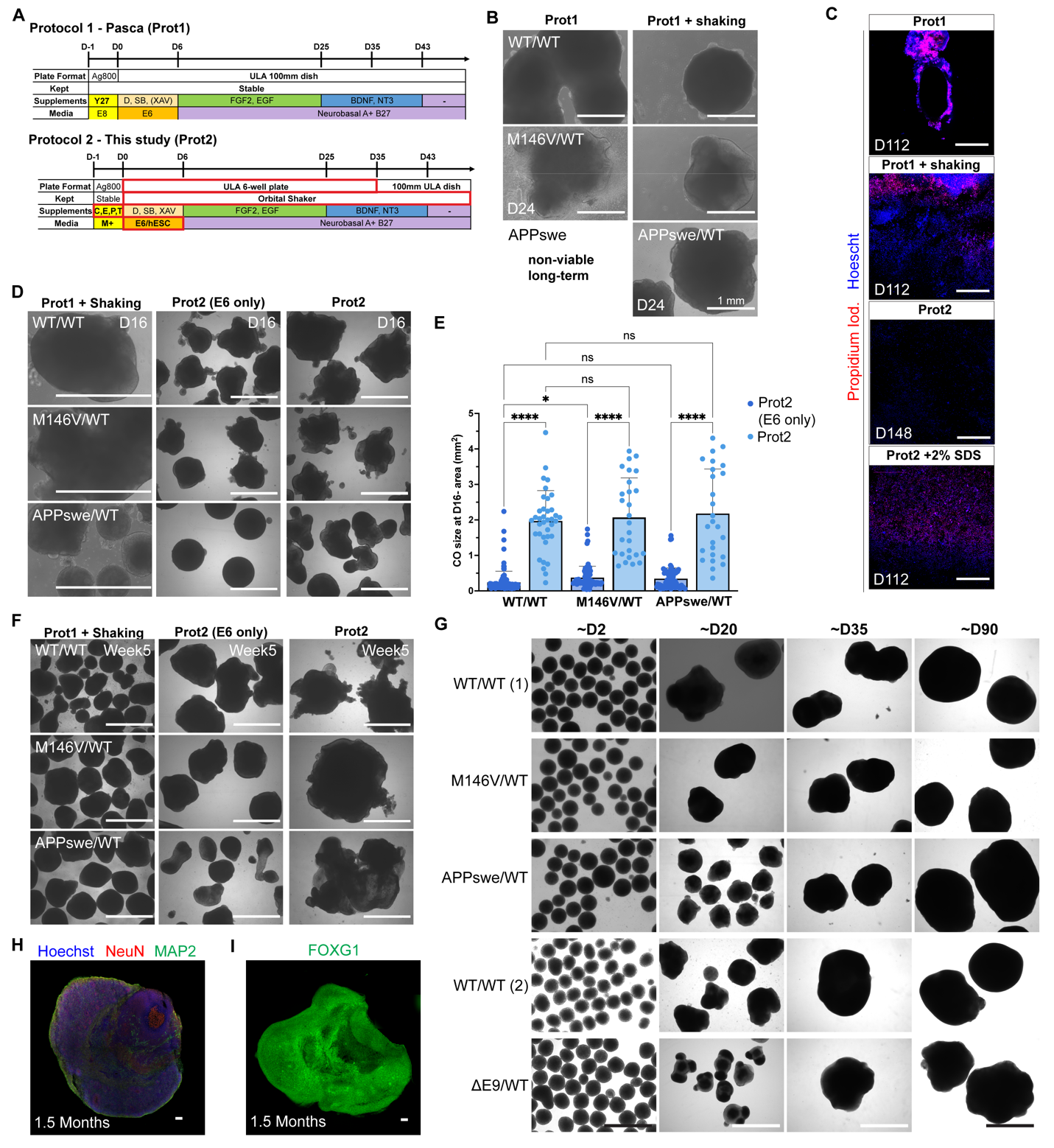
Supplementary Figure S1. Protocol modifications to generate reproducible, healthy COs across all genotypes.

1. Schematic summarizing the key modifications (Protocol 2, highlighted in red) made over the method of Pasca (Protocol 1). M+: mTeSR plus; C,E,P,T: Chroman 1, Emricasan, Polyamines, Trans-ISRIB; E6/hESC: 50/50 mix of Essential 6 and hESC media. See Experimental section for details. Representative bright field images of typical organoids maintained in 10 cm dishes (*left*) vs. in 6-well plates in an orbital shaker at day (D)24 (*right*). Scale bar, 1 mm.
2. APPSwe/WT COs live-stained with propidium iodide and imaged cross-sectionally after cutting in half to show their relative degree of necrosis across different protocol iterations. As positive control, one of the COs was pre-conditioned in media containing 2% SDS. Scale bar, 500 µm.
3. Representative bright field images showing the size and heterogeneity of isogenic sets of COs at D16 depending on the induction method. E6: Essential 6 medium (Gibco); Prot2: 50/50 mix of E6 and hESC medium.
4. Quantification of the mean CO size (maximal cross-sectional area) at D16 comparing the effect of the induction media, n = 77-109 COs per genotype for “E6 only”, n = 26-37 COs per genotype for E6/hESC; each condition from 4 independent CO induction experiments. Data are mean ± SD. Analysis by Welch’s ANOVA with Dunnett’s T3 post-hoc test.
5. Representative bright field images showing the size and heterogeneity of isogenic sets of COs at approximately D35 (Week 5) depending on the induction medium used.
6. Representative brightfield images of COs of each cell line produced with Protocol 2, at ~2, 20, 39, and 90 days in culture. Quantification shown in Figure 1B. Scale bar, 2 mm.
7. Representative immunofluorescent (IF) staining of mature postmitotic neurons (MAP2+, NeuN+) in COs at ~6-week timepoint. Scale bar, 100 µm.
8. Representative IF stain for forebrain (FOXG1+) fate in COs at 1.5-month timepoint. Scale bar, 100 µm.

#
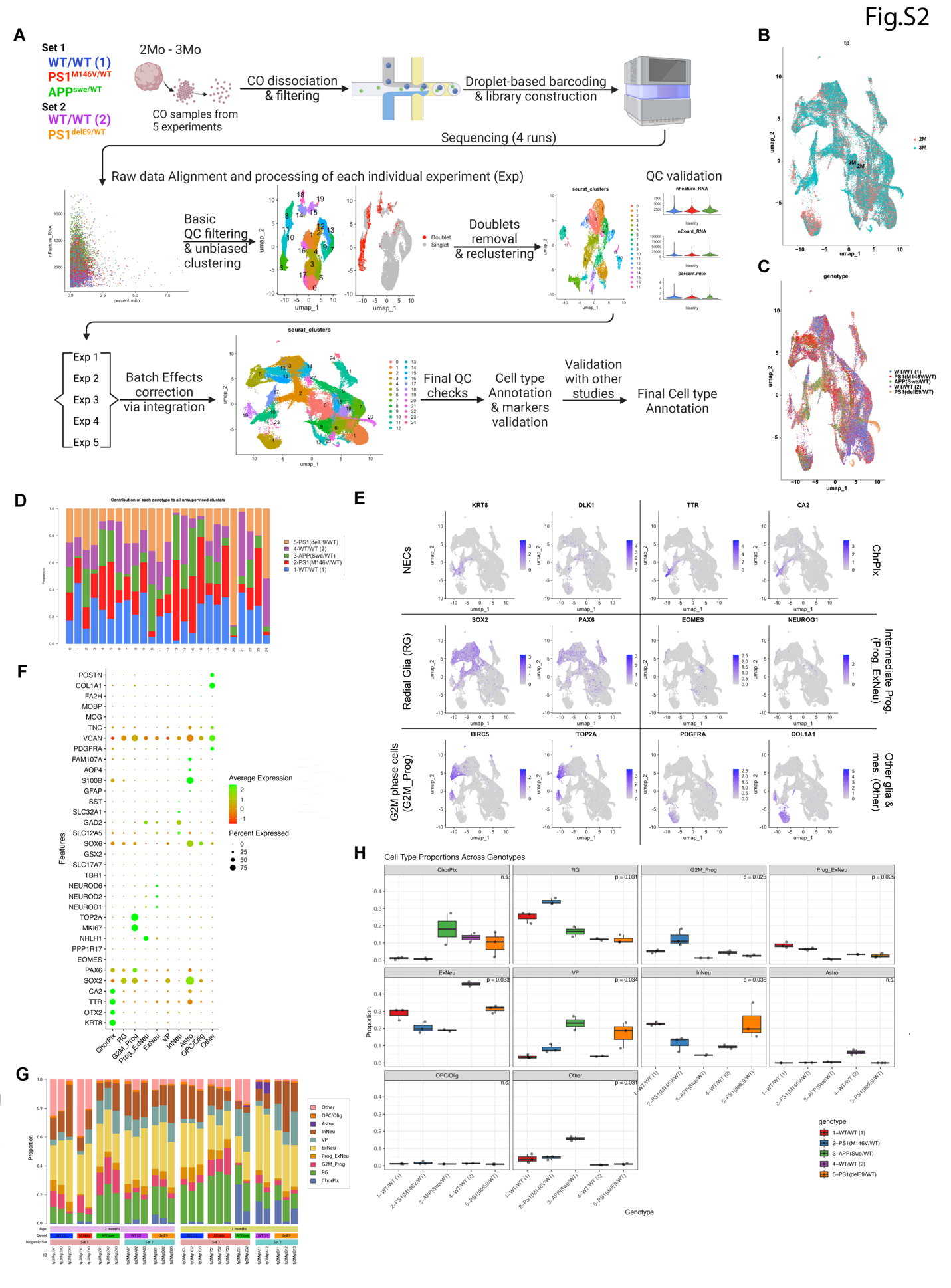


# Supplementary Figure S2. scRNA-seq cluster annotation and validation.

1. Diagram describing the sample and data processing of 5 experiments for the COs’ scRNA-seq along the respective UMAP clustering plots of the data before and after integration. The final set contained 70,854 high quality cells. See Experimental section for details.
2. UMAP cluster indicating the cell type mutant genotypes distribution (showing how they are similarly contributing to most clusters) along a breakdown of each gene mutant by their timepoint. 2-month in red; 3-month in aqua.
3. UMAP cluster indicating the cell type mutant genotypes distribution (showing how they are contributing to all UMAP cell clusters.
4. Bar plot breakdown showing an alternative representation of all CO genotypes contributing to all of the Seurat unbiased clusters.
5. Feature plots displaying the localized expression of hallmark markers for other identified CO cell types not already displayed in Fig.2B. Highlighted here are KRT8 and DLK1 for neuroepithelial cells (NECs); TTR and CA2 for choroid plexus (ChrPlx); SOX2 and PAX6 for Radial glia (RG); EOMES and NEUROG1 for intermediate progenitors (Prog_ExNeu); BIRC5 and TOP2A for cells in G2-M phase undergoing mitosis (G2M_Prog); and PDGFRA and COL1A1 for neural crest Schwann-like glia and mesenchymal progenitors (Other).
6. Dot plot of cell type and cell state hallmark genes across the annotated scRNA-seq groups to supplement Fig. 2C.
7. Proportions of all major cell types for each individual CO sample sequenced, organized by differentiation age stage, genotype, and experimental batch.
8. Box and whisker plots of the proportional representation of the different cell types in the 3-month timepoint COs across all genotypes. Analysis for each cell type by Kruskal- Wallis Rank Sum test.


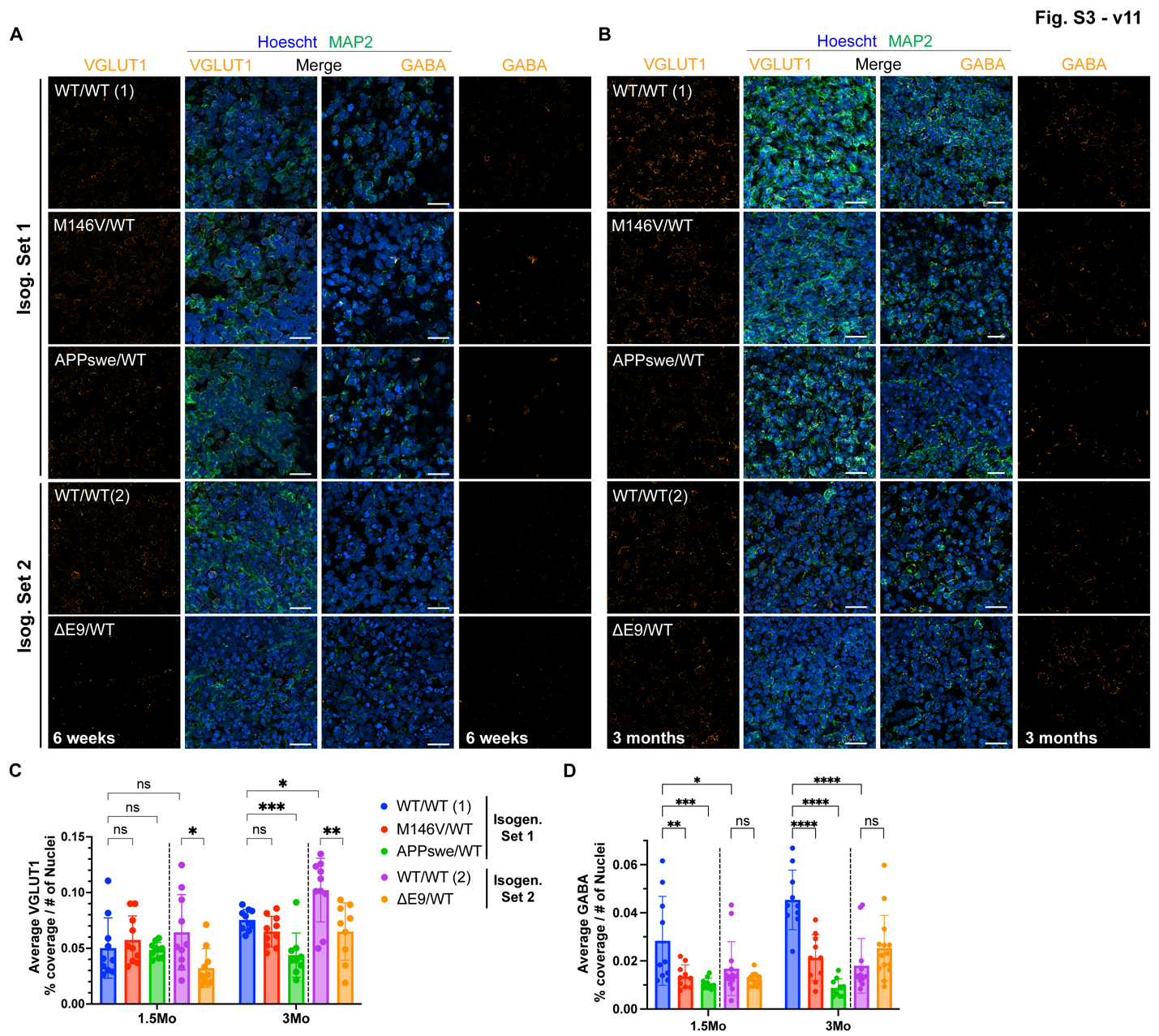


# Supplementary Figure S3. CO neuronal immunofluorescent validation.

(A and B) Representative Immunofluorescence (IF) images of 6-week (A) and 3-month

(B) timepoint COs for VGLUT1 (Excitatory neurons, *left*) and GABA (Inhibitory neurons, *right*) with Hoechst and MAP2 as pan-nuclear and mature postmitotic neuronal counterstains. Scale bar, 20 µm.

(C and D), Quantification of VGLUT1+ (C) and GABA+ (D) relative proportion as percent coverage area normalized to number of nuclei for 1.5- and 3-month timepoint COs, n = 9-10 images from 3-5 COs per condition per timepoint. Data are mean ± SD. Analysis by ANOVA with Dunnett’s post-hoc test.

#
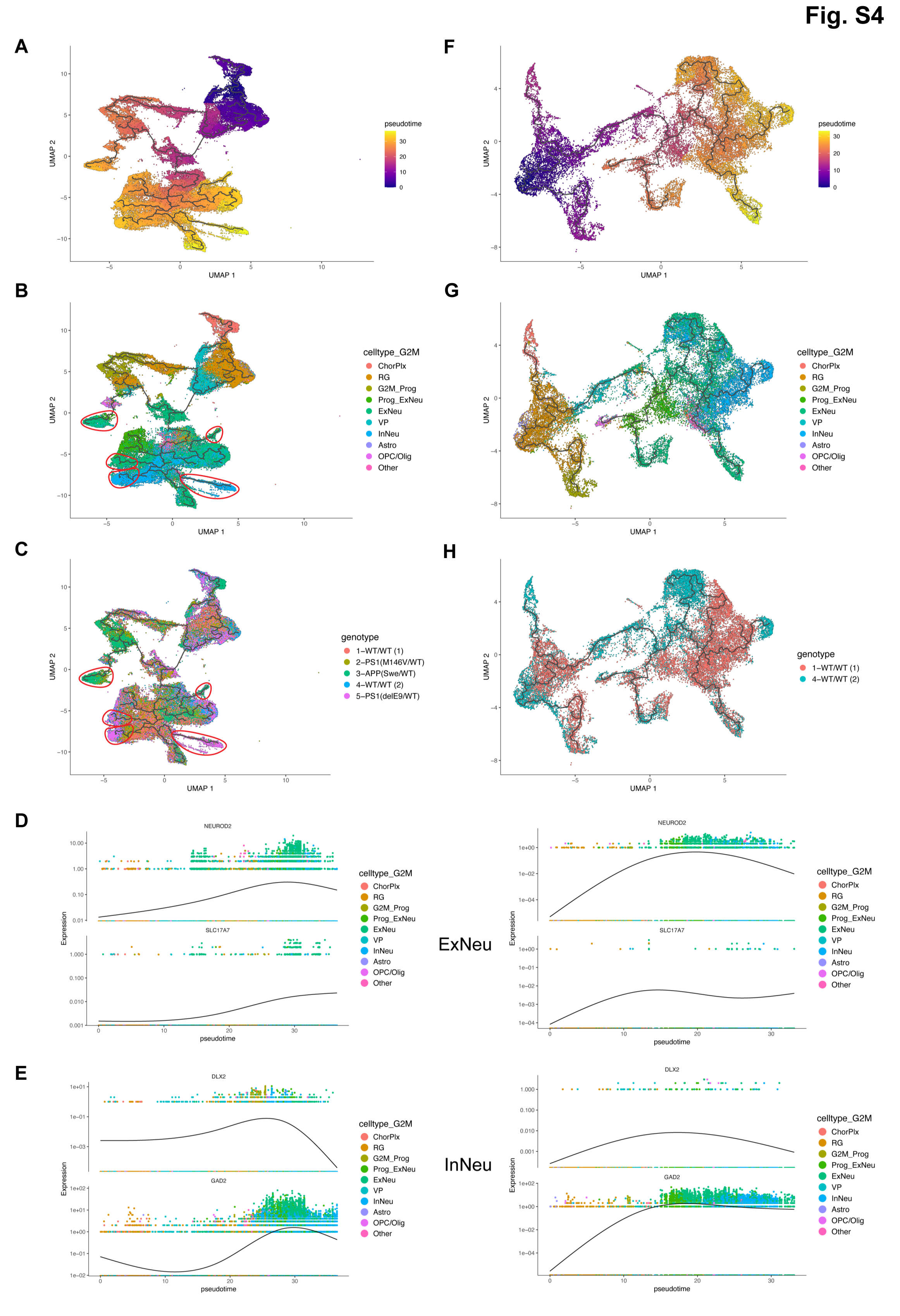
Supplementary Figure S4. Pseudotime analysis reveals mutant- specific maturation trajectories of neural lineages.

(A-C) UMAP visualization showing the inferred developmental progression of all 5 genotypes along CO lineage differentiation. Colored by pseudotime (A); colored by cell type annotation (B); colored by genotype (C).

1. Pseudotime plots of gene expression dynamics for immature ExNeu (NEUROD2) and mature ExNeu (SLC17A7), marking ExNeu lineage progression. All genotypes (*left*); WT/WT (from isogenic set 1) and WT/WT (from isogenic set 2) (*right*).
2. Pseudotime plots of gene expression dynamics for immature InNeu (DLX2) and mature InNeu (GAD2), marking InNeu lineage progression. All genotypes (*left*); only WT/WT (1) & WT/WT (2) genotypes (*right*).

(F-H) UMAP visualization showing the inferred developmental progression of cells from the WT/WT genotypes (1 & 2) along CO lineage differentiation. F: colored by pseudotime; G: colored by cell type annotation; H: colored by genotype.

RG: radial glia; Prog_ExNeu: excitatory neurons progenitors; ExNeu: excitatory neurons; InNeu: inhibitory neurons; Astro: astrocytes; OPC/Olig: oligodendrocyte progenitors/oligodendrocytes; ChorPlx: choroid plexus cells.


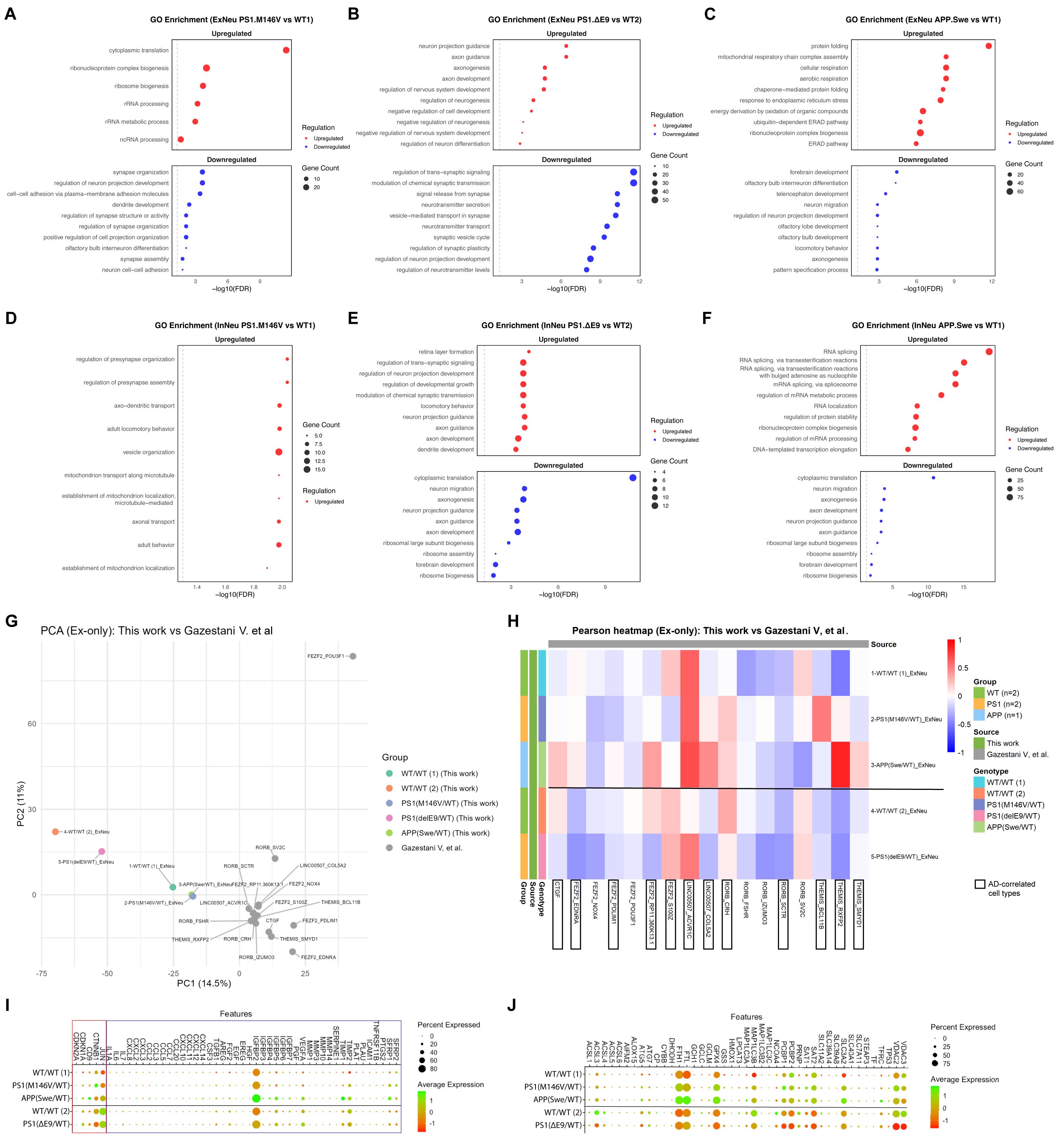


Supplementary Figure S5. Transcriptomic analysis of AD vs WT COs.

(A-C) GO Enrichment in Excitatory neurons (ExNeu) for PSEN1^M146V^/WT (A) PSEN1^ΔE9^/WT (B), and APP^Swe^/WT (C) vs their respective isogenic WT genotypes.

(D-F) GO Enrichment in Inhibitory neurons (InNeu) for PSEN1^M146V^/WT (D) PSEN1^ΔE9^/WT (E), and APP^Swe^/WT (F) vs their respective isogenic WT genotypes.

(G) PCA plot of the ExNeu CO populations of the current study and the excitatory neuron populations identified in the early AD meta-analysis by Gazestani et al.^[54]^ The AD CO transcriptomic signatures are closer to the early AD cell types than their respective isogenic WT genotypes.

(H) Heatmap plot comparing the Pearson correlation scores between the transcriptomic signature of the major CO ExNeu of the current study and that of the excitatory neuronal subtypes identified in the Gazestani et al. meta-analysis.^[54]^ Highlighted are the neuronal subtypes the meta-analysis correlated with increasing histopathological burden.

(I) Dot plot of Senescence markers pseudo-bulk expression across genotypes. Boxed in red are common neuronal senescence markers like p21 (CDKN1A), p16 (CDKN2A), etc.; boxed in blue are the senescence-associated secretory phenotype (SASP) genes.

(J) Dot plot of ferroptosis markers pseudo-bulk expression across genotypes.


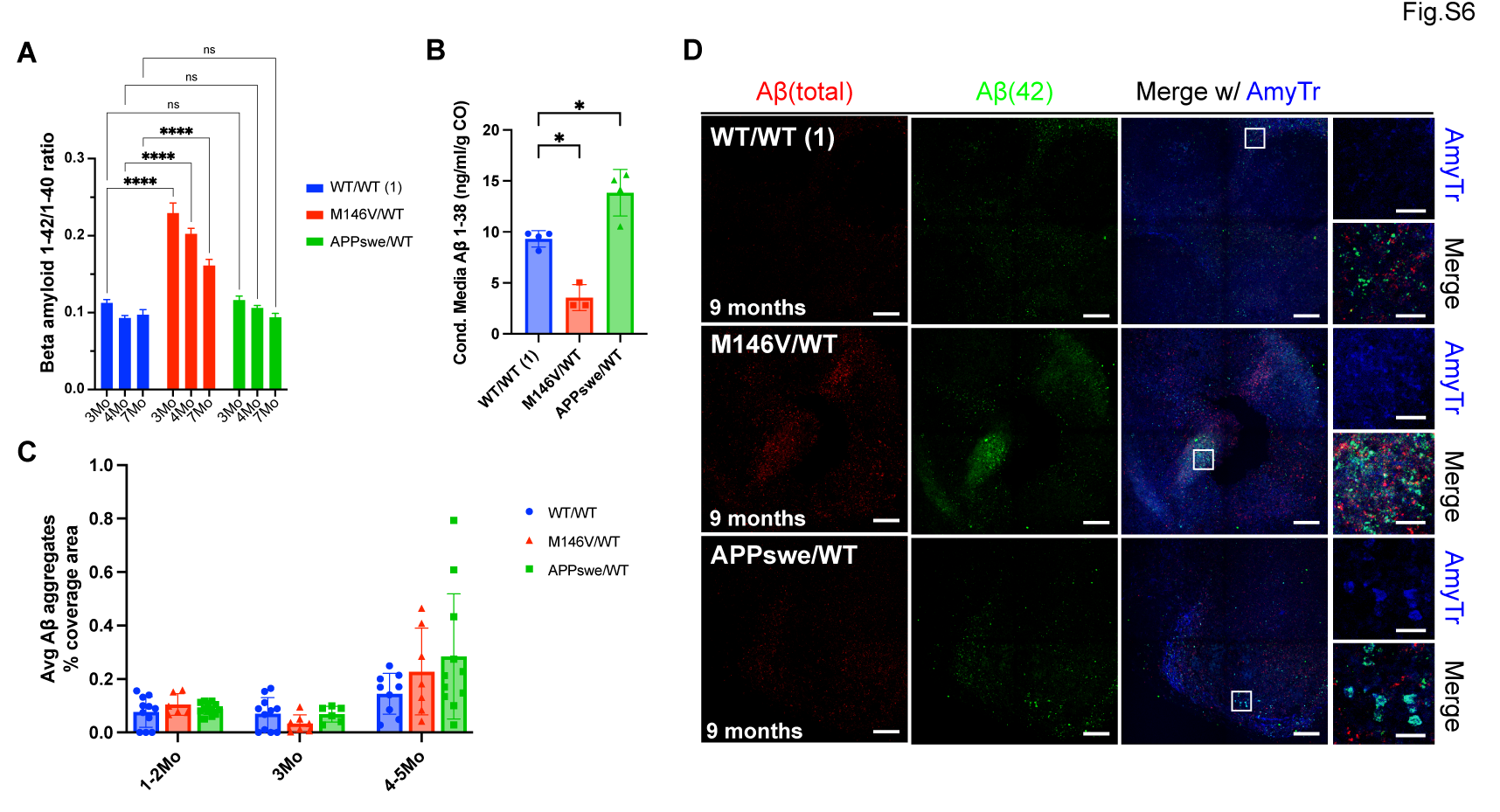


Supplementary Figure S6. AD COs accumulate amyloid+ Aβ aggregates after 5 months of age despite Aβ_1-42_ /Aβ_1-40_ soluble peptide ratios being elevated earlier.

(A) Ratio in CO-conditioned media of Aβ_1-42_ to Aβ_1-40_ at 3, 4, and 7-month timepoints. n = 3-4 COs per condition. Analysis by ANOVA with Dunnett’s post-hoc test.

(B) Aβ_1-38_ peptide concentration in conditioned media from 5-6 months-of-age COs for isogenic set 1. Concentrations were below quantifiable levels for COs from isog. set 2. Analyses by ANOVA with Dunnett’s post-hoc test.

(C) Immunostaining quantification of Aβ aggregates in COs at 1 to ~5 months of age with a total Aβ antibody, represented as total Aβ+ area normalized to the organoid area in field of view. n = 5-11 images from 3-6 COs per condition.

(D) Immunostaining of isogenic COs at 9-month time point with the amyloid stain, AmyTracker (AmyTr), and total and Aβ_1-42_-specific Aβ antibodies. Scale bar, 100 µm.

Data are mean ± SD. Analysis by non-parametric Mann-Whitney test due to non-normal distribution.


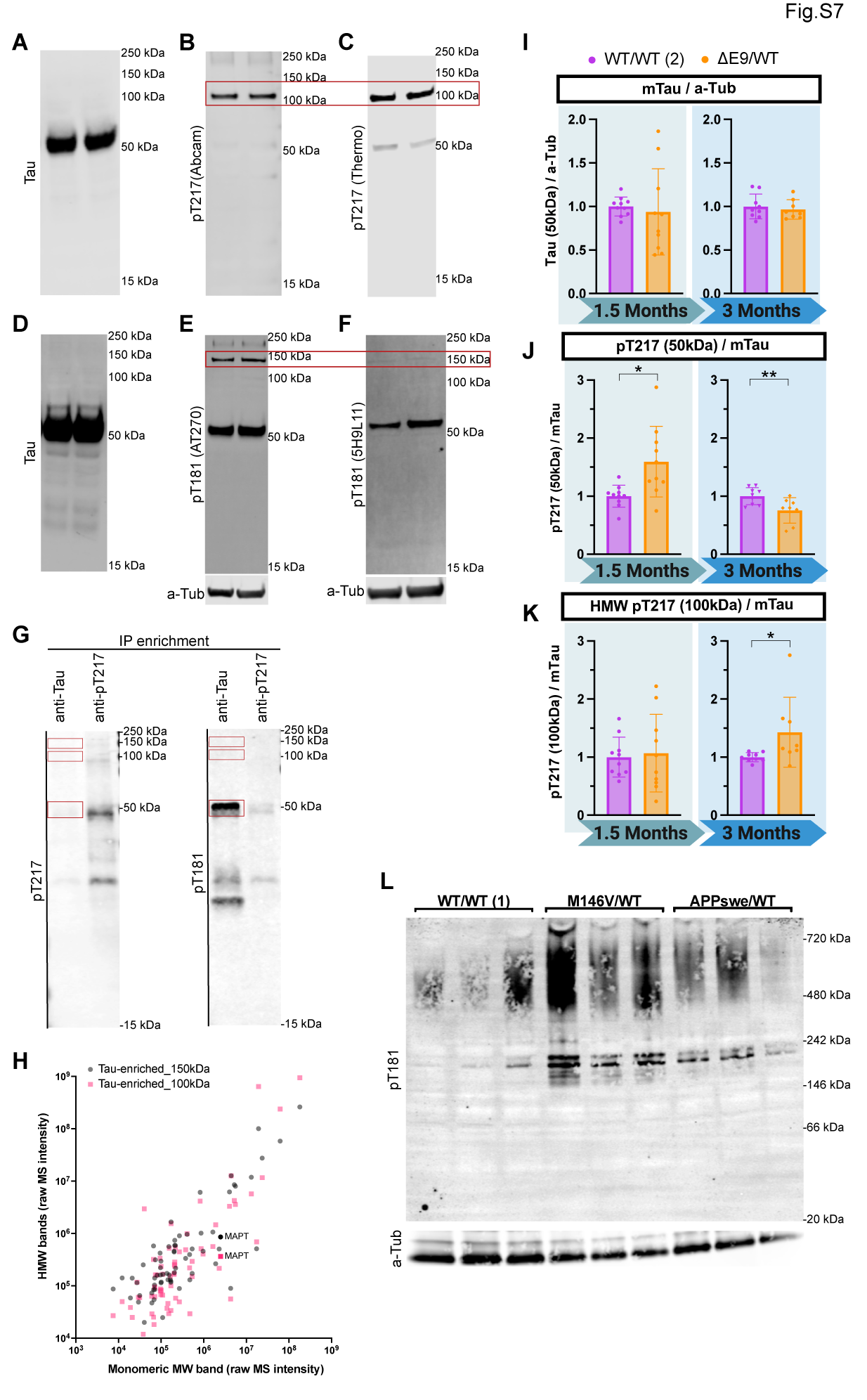


Supplementary Figure S7. Additional validation of Tau and pTau pathology.

(A-C) WB of two PSEN1^ΔE9^/WT 1.5 months-of-age CO lysates probed with the pan-Tau antibody (mTau) (A) or with p217 antibody (Thermo AT270, RRID: AB_223651) after incubation with a saturating concentration of pan-Tau antibody (B). (C) Replicate WB of the same CO lysates probed with a different phospho-Tau T217 antibody, clone 5H9L11 (Thermo, RRID: AB_2532491). Red box highlights the consistent HMW band persisting in the reduced SDS-PAGE.

(D-F) WB of two independent lysates from PSEN1^ΔE9^/WT 1.5-month timepoint COs probed with the pan-Tau antibody (mTau) (D) or with pT181 antibody (Thermo AT270, RRID: AB_223651) after incubation with a saturating concentration of pan-Tau antibody (E). (F) WB replicate of the same CO lysates probed with a different phospho-Tau T181 antibody, clone 5H9L11 (Thermo, RRID: AB_2532491). Red box highlights the consistent HMW band persisting in the reduced SDS-PAGE.

(G) WB of immunoprecipitate (IP) from lysate of 3 month-old PSEN1^M146V^/WT CO to enrich for Tau or pT217, as probed by different pT217 and pT181 antibodies. Red boxes indicate bands directly assessed by MS.

(H) Plot of identified hits in IP-enriched samples (68 unique proteins) by MS intensity. Tau (MAPT) was identified in all both HMW bands.

(I) WB Quantification of monomeric Tau (mTau) in isogenic set 2 CO lysates at the 1.5- (*left*) and 3-month (*right*) timepoints, as normalized to ɑ-Tubulin (a-Tub). n = 8-10 COs per genotype from 3 independent experiments per timepoint.

(J-K) WB Quantification of phospho-Tau pT217 at its monomeric (~50 kDa)(J), and strongest (~100 kDa)(K) bands in isog. set 2 CO lysates at the 1.5 and 3-month timepoints, as normalized to mTau. n = 8-10 COs per genotype from 3 independent experiments per timepoint.

(L) Representative native PAGE of 4-month timepoint CO lysates probed for pT181 and ɑ-Tubulin. Data are mean ± SD. Analyses by ANOVA with Dunnett’s post-hoc test.


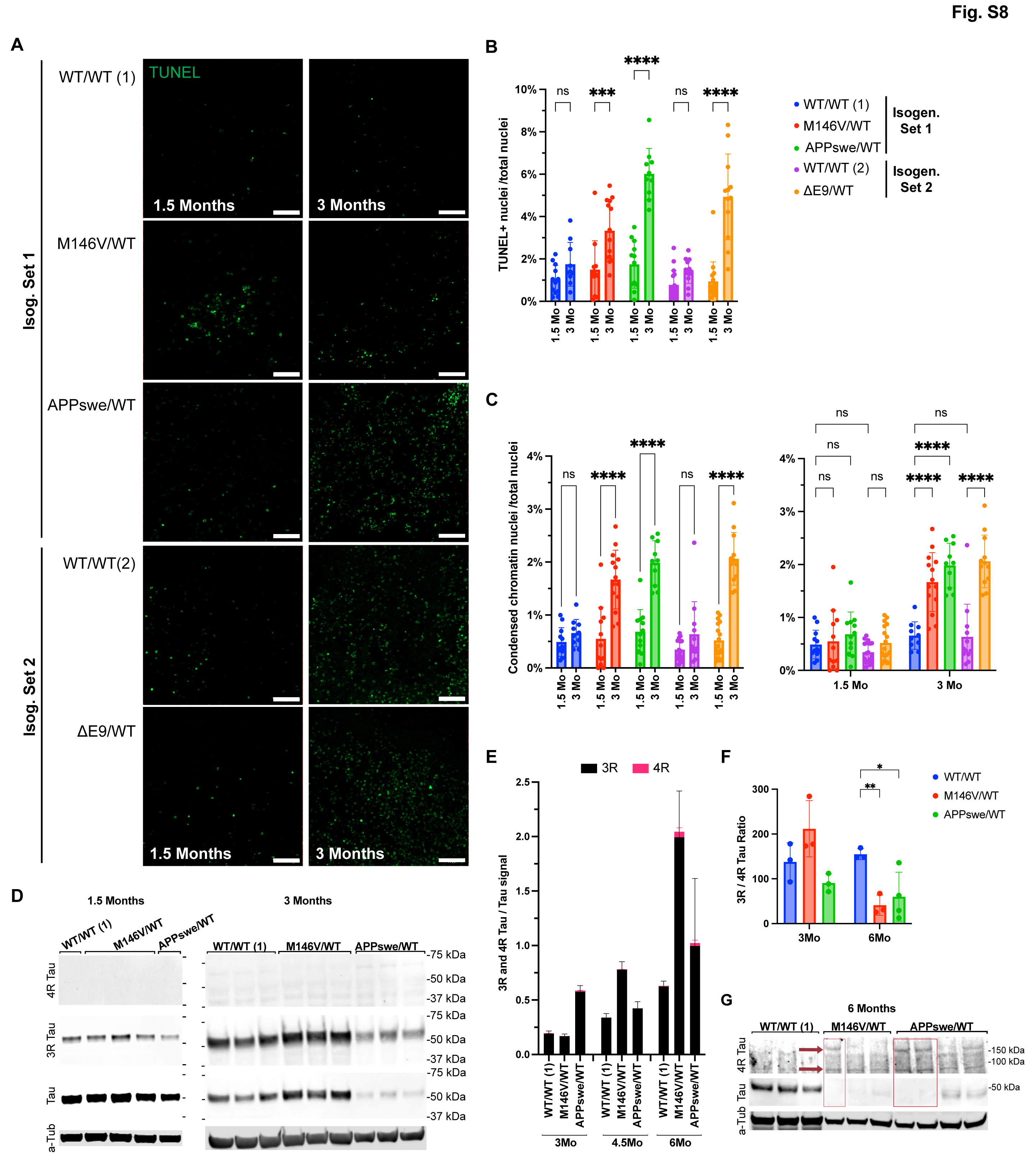


# Supplementary Figure S8. AD COs display signs of neurodegeneration prior to aberrant accumulation of 4R Tau.

(A) Representative images of 6 weeks and 3 months-of-age COs for TUNEL (Terminal deoxynucleotidyl transferase dUTP nick end labeling) staining for detecting apoptotic and/or necroptotic cells. Scale bar, 50 μm.

(B and C) Independent quantification of either the total number of TUNEL+ nuclei (B) or the number of nuclei with condensed chromatin (C), normalized to the total number of nuclei. Total number and chromatin condensation counts were both assessed by overexposed post-fixation staining with Propidium Iodide to visualize all nuclei, n = 12-17 images from at least 3 COs per condition. Data are mean ± SD. Analysis by ANOVA with Sidak’s post-hoc test, including within timepoints (C, *right*).

(D) Representative immunoblots of 1.5- and 3-month-old COs for R4 Tau, R3 Tau, pan-Tau, and alpha-tubulin (a-Tub).

(E) Bar plot of the raw 3R and 4R Tau isoform to pan-Tau ratio signal in COs at 3-, 4.5-, and 6-month timepoints. Data are mean ± SD. Analysis by ANOVA with Dunnett’s post-hoc test.

(F) R3:R4 Tau isoform ratio at the 3- and 6-month timepoints.

(G) Representative immunoblot of 6-month-old COs showcasing HMW bands in the 4R Tau blot. Highlighted in red, those samples with the weakest mTau signal.


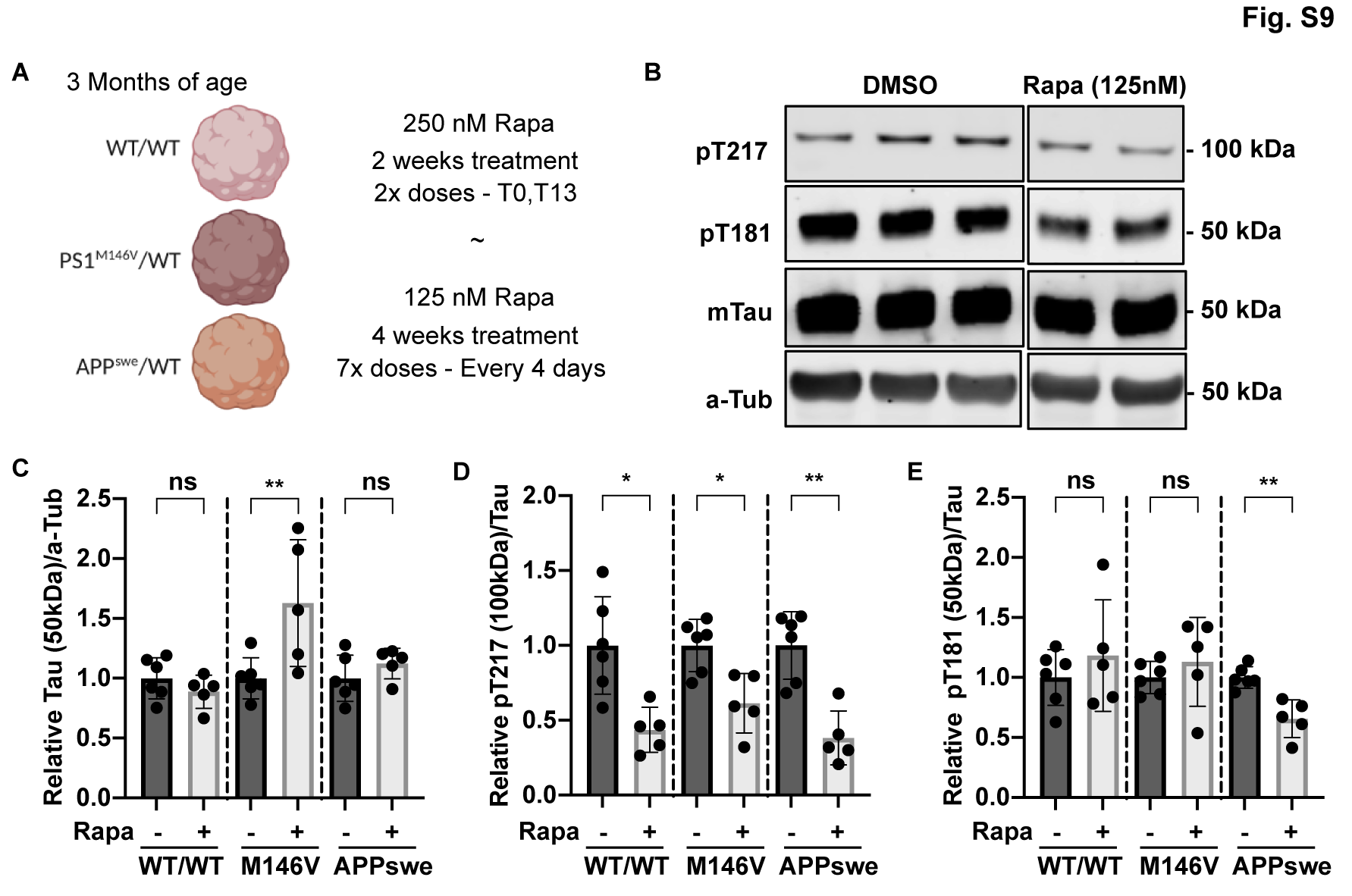


# Supplementary Figure S9. Chronic mTOR-dependent autophagy activation reverses AD-associated pTau pathologies.

(A) Diagram describing the experimental set-up of the 2-week-long and 4-week-long mTOR-mediated Rapamycin (Rapa) treatment of 3-month timepoint (by end of treatment) COs.

(B) Representative WB of CO lysates at the end of the 2-week-long treatment probed for their Tau and relative pTau abundances.

(C-E) WB Quantification for the normalized monomeric Tau (C), pT217 (~100kDa) (D), and pT181 (~50 kDa) (E) abundances after the treatment. Bands at other molecular weights were too low to quantify. n = 5-6 COs per genotype per condition from 2 independent experiments. Data are mean ± SD. Analyses by ANOVA with Dunnett’s post-hoc test.

#
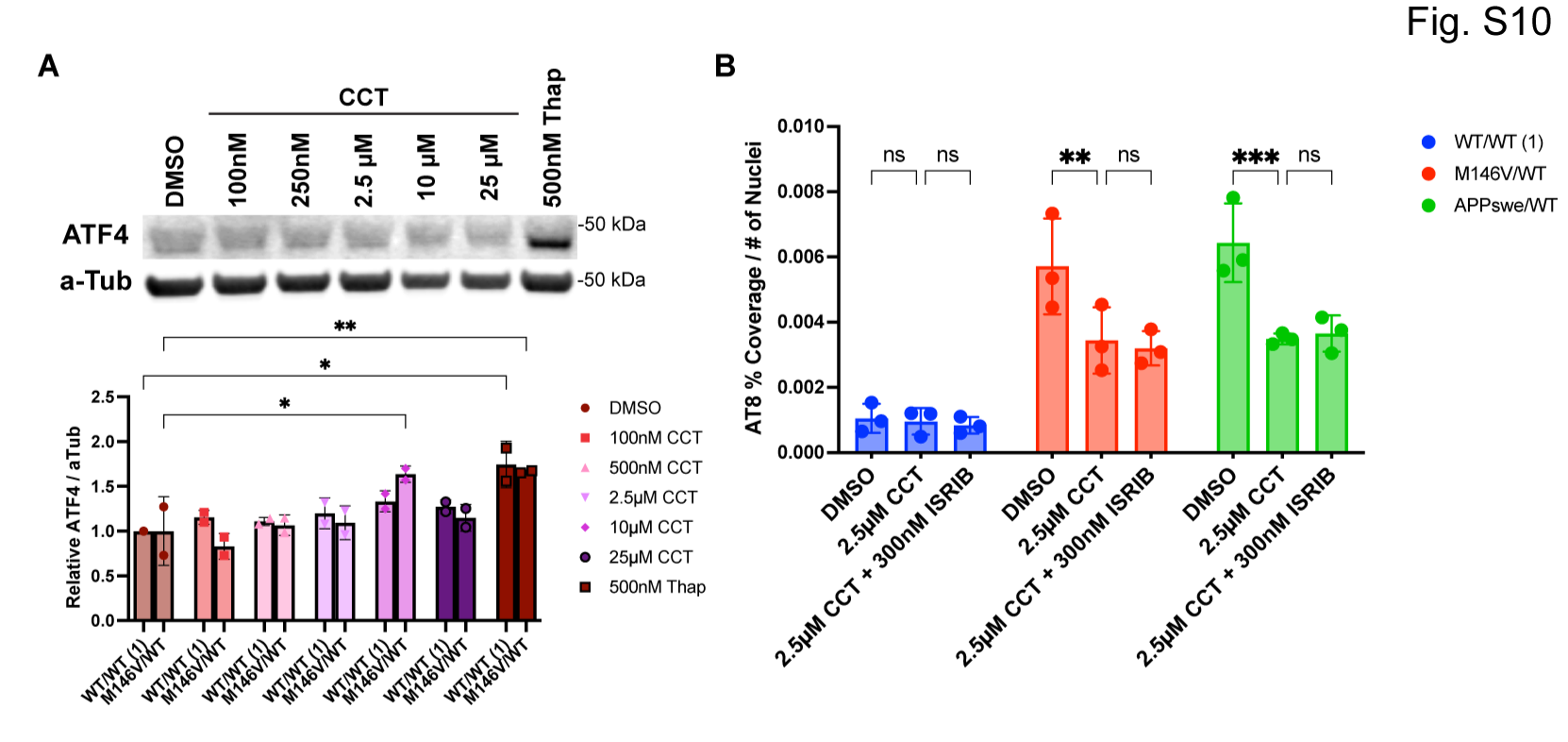
Supplementary Figure S10. CCT treatment does not activate or rely on PERK at concentrations used in the study.

(A) Representative WB of lysed COs after 6-day treatment with DMSO vehicle, 100 nM – 25 µM CCT, or 500 nM Thapsigargin (as a PERK activator positive control) probed for ATF4. COs were treated on day (D)0, and D5, and lysed 24 hours after the second treatment day. Quantification of ATF4 signal normalized to a-tubulin (a-Tub) and relative to DMSO value.

(B) Quantification of the paired helical filament (PHF) aggregated pTau (AT8+) percent coverage normalized to nuclei number after 2-week treatment with 2.5 µM CCT with or without 300 nM ISRIB (integrated stress response inhibitor). n = 3 COs per condition. Data are mean ± SD. Analyses by ANOVA with Dunnett’s post-hoc test.

#
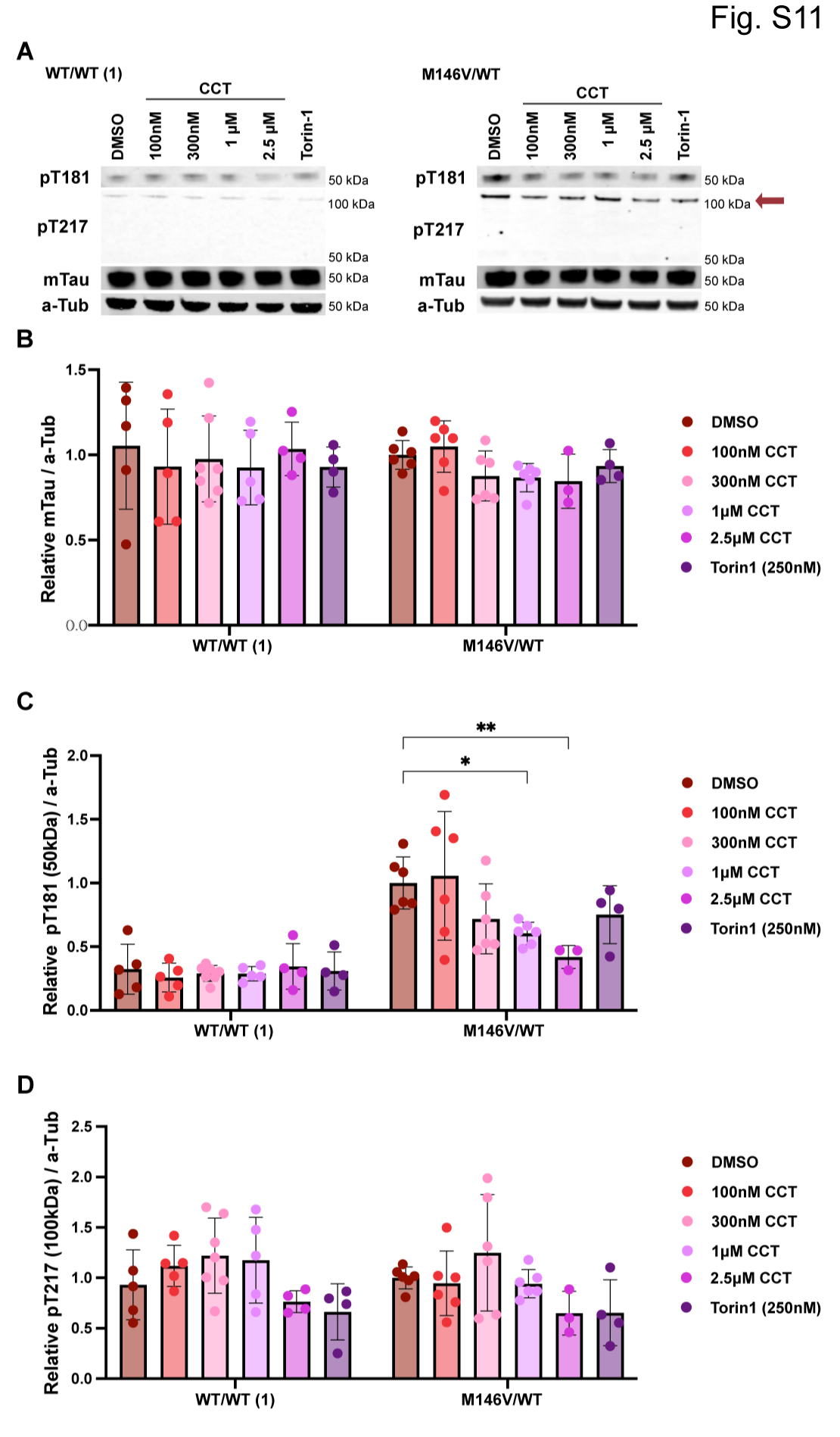


# Supplementary Figure S11. Chronic autophagy activation differentially reduces AD-associated pT217 and pT181.

(A) Example WBs of the WT/WT (1) and PSEN1^M146V^/WT COs probed for monomeric Tau (mTau), phosphorylated Tau 181 (pT181), phosphorylated Tau 217 (pT217), and a- tubulin (a-Tub) at the 3-month timepoint after a 4-week treatment with DMSO vehicle, CCT, or Torin-1.

(B-D) WB Quantification of the WT/WT and PSEN1^M146V^/WT CO lysates for a- Tub-normalized mTau (B), pT181 (C), and pT217 (D) at the end of a 6-week-long treatment. Data from multiple experiments are normalized to mean PSEN1^M146V^/WT DMSO values via inter-blot controls. n = 12-23 COs per genotype from 2 independent experiments. Data are mean ± SD. Analysis by ANOVA with Dunnett’s post-hoc test.

#
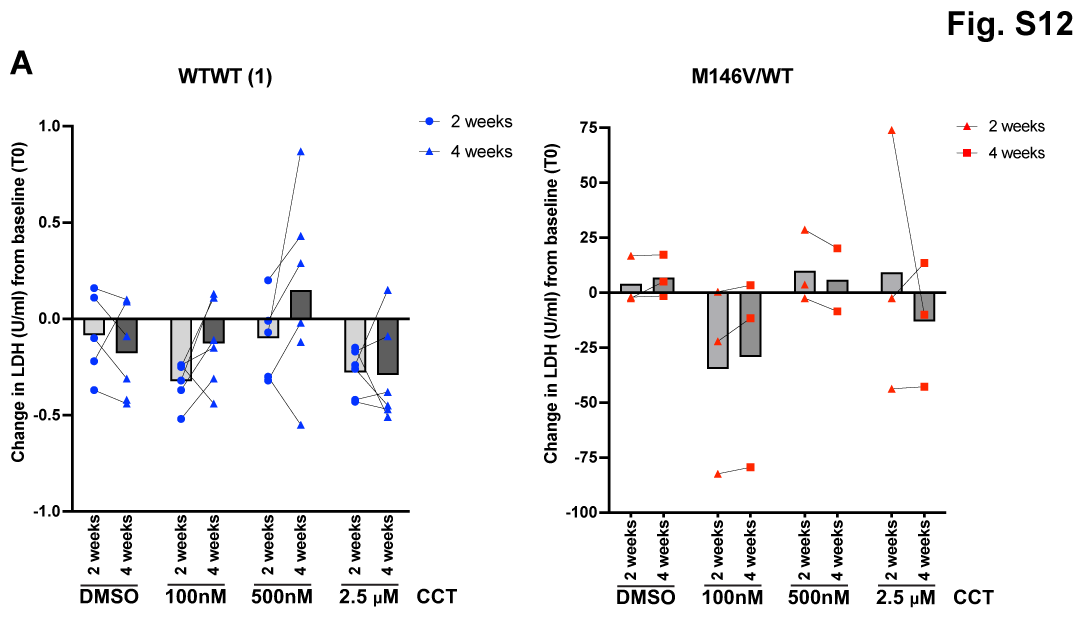
Supplementary Figure S12. Additional validation of Tau and pTau pathology.

(A) Quantification of LDH in conditioned media after 2 and 4 weeks of treatment with DMSO vehicle vs. 100 nM, 500 nM, or 2.5 µM CCT for WT/WT (1) and PSEN1^M146V^/WT COs. n = 5-6 COs per condition for WT/WT (1); n = 3 COs per condition for PSEN1^M146V^/WT.
